# Dynamics of *Co-Transcriptional* Pre-mRNA Folding Influences the Induction of Dystrophin Exon Skipping by Antisense Oligonucleotides

**DOI:** 10.1371/journal.pone.0001844

**Published:** 2008-03-26

**Authors:** Keng Boon Wee, Zacharias Aloysius Dwi Pramono, Jian Li Wang, Karl F. MacDorman, Poh San Lai, Woon Chee Yee

**Affiliations:** 1 Neuromuscular Research Laboratory, National Neuroscience Institute, Singapore, Singapore; 2 Department of Neurology, National Neuroscience Institute, Singapore, Singapore; 3 Bioinformatics Institute, Singapore, Singapore; 4 School of Informatics, Indiana University, Indianapolis, Indiana, United States of America; 5 Department of Paediatrics, Yong Loo Lin School of Medicine, National University of Singapore, Singapore, Singapore; 6 Graduate School for Integrative Sciences and Engineering, Centre for Life Sciences, National University of Singapore, Singapore, Singapore; Ecole Normale Supérieure de Lyon, France

## Abstract

Antisense oligonucleotides (AONs) mediated exon skipping offers potential therapy for Duchenne muscular dystrophy. However, the identification of effective AON target sites remains unsatisfactory for lack of a precise method to predict their binding accessibility. This study demonstrates the importance of *co-transcriptional* pre-mRNA folding in determining the accessibility of AON target sites for AON induction of selective exon skipping in DMD. Because transcription and splicing occur in tandem, AONs must bind to their target sites before splicing factors. Furthermore, *co-transcriptional* pre-mRNA folding forms transient secondary structures, which redistributes accessible binding sites. In our analysis, to approximate transcription elongation, a “window of analysis” that included the entire targeted exon was shifted one nucleotide at a time along the pre-mRNA. Possible c*o-transcriptional* secondary structures were predicted using the sequence in each step of transcriptional analysis. A nucleotide was considered “*engaged*” if it formed a complementary base pairing in all predicted secondary structures of a particular step. Correlation of frequency and localisation of *engaged* nucleotides in AON target sites accounted for the performance (efficacy and efficiency) of 94% of 176 previously reported AONs. Four novel insights are inferred: (1) the lowest frequencies of *engaged* nucleotides are associated with the most efficient AONs; (2) *engaged* nucleotides at 3′ or 5′ ends of the target site attenuate AON performance more than at other sites; (3) the performance of longer AONs is less attenuated by *engaged* nucleotides at 3′ or 5′ ends of the target site compared to shorter AONs; (4) *engaged* nucleotides at 3′ end of a short target site attenuates AON efficiency more than at 5′ end.

## Introduction

Antisense oligonucleotides (AONs) are synthetic single-stranded molecules, typically consisting of 16 to 30 nucleotides that are complementary to a specific sequence in the target RNA. Apart from their well-documented applications to suppress gene expression, AONs have been used to modulate pre-messenger RNA (pre-mRNA) splicing as potential therapeutic strategy for genetic diseases such as Duchenne muscular dystrophy or DMD [1]–[13], thalassemia [14]–[17], ocular albinism [18] and cancer [19]. Studies of AON in DMD (MIM #310200), a fatal X-linked disorder affecting 1 in 3300 newborn males caused by mutations in the dystrophin gene, have progressed to preliminary human trials [7], [8], [20]–[26]. The strategy involves selective exon skipping, either to remove the mutation carried by the exon, for point mutations, or to restore the mRNA reading frame, for frame-shift mutations. Although the resulting protein will be shorter than the wild type, it is expected to reduce the severe symptoms of DMD to the much milder allelic form of the disease, Becker muscular dystrophy (BMD, MIM #300376) [1]–[5]. Restoration of widespread dystrophin expression by AONs has been demonstrated in animal models [21], [23], [25]. Currently, the first phase I/II clinical trials of AON for DMD therapy are being initiated [27].

The design of AONs for exon skipping of dystrophin involves the selection of appropriate AON target sites using mfold [28] and other similar computational tools [29]–[31] for prediction of pre-mRNA secondary structure. However, the conventional applications of mfold for selecting AON target sites are not satisfactory [9], [32], [33]. For example, Aartsma-Rus *et al.* [10] concluded that, using mfold to predict the secondary structure of targeted pre-mRNA, they still had no clear insight into the accessibility of the targeted sequence within the folded pre-mRNA structure. We hypothesize that this outcome may be due to the omission in considering the dynamic localization of accessible sites during the ‘opportune period’ of pre-mRNA transcription. We propose that this omission may underlie the low success rate in the design of effective and efficient AONs.

An AON induces exon skipping by competitive binding at its target site against splicing factors during transcription [2], [12]. Splicing factors form the 60S splicing machinery called the spliceosome that removes the introns while retaining the exons during pre-mRNA processing [34]. These splicing factors bind to important sequences in the pre-mRNA, which include donor and acceptor splice sites, branch points (BP), pyrimidine tracts and exon splicing enhancers (ESEs) [35]. Blocking these sites with AONs prevents the spliceosome from identifying the targeted region as an exon, which will thereby be removed along with the introns. Because of long introns sequences in dystrophin, ESE-dependent exon identification [36]–[41] is particularly important (Figure S1 and figure S2 of the *Online Supporting Information*). Indeed, AONs targeted to ESEs showed specific and effective induction of exon skipping in human tissue or cells [7]–[13] but resulted in unpredictable skipping of adjacent exons when targeted to the splice sites [9], [42], [43] in DMD.

The splicing of introns by the spliceosome [34] is considered *co-transcriptional* [44]–[52], as it happens simultaneously during transcription [53], [54] of the pre-mRNA, at the point when an exon and its flanking introns are defined in the nascent pre-mRNA. *Co-transcriptional* splicing of dystrophin gene was first reported by Tennyson *et al* [45] in which the authors observed that “spliced transcript accumulates first at the 5′ end of the gene and at progressively later times as one moves further downstream from the muscle promoter” over a time period consistent with *co-transcriptional* splicing. The authors argued that given the exceptionally large size of the gene and large numbers of exons, *co-transcriptional* splicing is an effective way to limit the number of possible splice sites and thereby decrease the probabilities of incorrect splicings.

Recent experimental results support the notion that the transcription and splicing machineries are intricately coupled (reviewed by Maniatis T. & Reed R. [49]). Specifically, by being tethered to both the RNA polymerase II and transcription elongation factors, splicing factors are localized directly adjacent to the nascent pre-mRNA emerging from the polymerase. This indicates that *co-transcriptional* exon recognition occurs at the proximity of the emerging nascent transcript, which seems to be supported by identical observations of both Aartsma-Rus *et al.* [10] and Wilton *et al.* [6]. They reported that AONs targeting either acceptor sites or ESE sites in the first half of the exon are generally more efficient in inducing exon skipping than at the other half. This suggests that *co-transcriptional* exon recognition not only occurs as soon as recognition sites are transcribed, it is efficient as well; this implies that competition for binding to exon recognition sites starts as soon as they are transcribed. Together with the fact that *co-transcriptional* exon recognition precedes *co-transcriptional* intron removal (splicing), we conjecture that effective AONs must bind to their target sites during *co-transcriptional* target exon recognition.

To be efficacious, an AON must bind to an effective target site at the right time. An effective target site is a pre-mRNA sequence containing functional ESEs within the exon to be skipped. The right time or ‘opportune period’ is before splicing factors bind to the AON target site. Thus, two major factors defining AON efficiency are (1) binding to functional ESEs within the target site by the AON and (2) accessibility of the target site to binding during the ‘opportune period’, which in turn depends on the secondary structure of the pre-mRNA. The tendency to form complementary base pairings among the nucleotides within the pre-mRNA may cause a target site to be inaccessible, as a nucleotide that is “paired” is not accessible for binding. However, there are certain regions in the pre-mRNA with secondary structure motifs devoid of base pairing, such as loops, bulges, joint sequences and free 3′ or 5′ ends [55]. Hence, for optimal AON binding, the prediction of base pairings and secondary structure motifs of the target site is likely to be crucial. As *co-transcriptional* pre-mRNA folding will lead to dynamic and transient secondary structures [56]–[60], which in turn result in dynamic and transient nucleotide base pairing, the *co-transcriptional* folding of the nascent pre-mRNA during the “opportune period” must also be taken into account in determining optimal AON binding.

**Figure 1:**
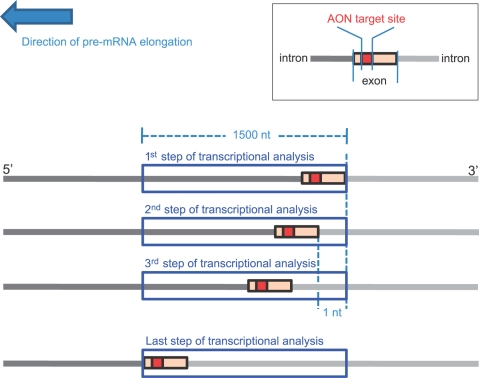
Approximation of the transcriptional elongation process of pre-mRNA. To approximate the transcription elongation process, a “window of analysis” is shifted one nucleotide at a time along the pre-mRNA sequence towards the 3′ end, beginning with the 3′ end of the exon targeted to be skipped at the window's 3′ end and stopping when the 5′ end of the targeted exon reaches the 5′ end of the window. Each window of analysis corresponds to a step of transcriptional analysis at which the possible secondary structures of its sequence were predicted.

To test this hypothesis, we developed novel scoring methodologies to semi-quantify the *co-transcriptional* binding accessibility of AON target sites. The AON target sites of 2 sets of published AONS were scored for c*o-transcriptional* binding accessibility and their scores correlated with the degree of reported AON efficiency and efficacy. The scoring methodologies are based on the application of an established software (i.e. mfold) for secondary structure prediction in conjunction with a method to approximate the dynamics of transcription. A “window of analysis” of pre-determined sequence length of 1500 nucleotides that includes the full length of the targeted exon (see *Methods and Materials*) corresponds to a “step of transcriptional analysis”. To approximate the transcription elongation process, the window of analysis is shifted one nucleotide at a time along the pre-mRNA sequence towards the 3′ end (Figure 1). At each step of transcriptional analysis, the possible secondary structures for the window sequence are predicted with mfold. Subsequently, each nucleotide within the AON target site will be scored for binding accessibility based on whether it is paired in the predicted secondary structures.

## Results

A total of 176 AONs, reported by two independent sources [10] and [6], that target ESEs to induce exon skipping in dystrophin pre-mRNA was analyzed. Although the cell lines and experimental protocols used in these two studies were similar, the AONs from each study were analyzed separately because of the following reasons. The range of AON lengths, which may influence AON performance [61], differed significantly between the studies. The AONs from the two sources [10] and [6] showed median lengths of 19 and 26 nucleotides respectively, and for the purpose of this study, are henceforth denoted as Set A and Set B respectively. Secondly, the respective sources graded their AONs differently. In Set A, AONs were graded as (++), (+) or (−) if their efficiencies were >25%, <25% or 0% (i.e., non-effective) respectively. In Set B, AONs are graded as (++), (+^1^), (+^2^) or (−) if their efficiencies were >30%, 10%–30%, <10% or 0% respectively. For our analysis, grades (+^1^) and (+^2^) of Set B were merged into a single grade (+) while retaining the other grades as used by the respective sources.

Four levels of analysis using scoring methodologies of increasing complexity were used to score the accessibility of AON target sites. Scores at each level of analysis were then correlated with AON efficiency and efficacy for each of the two sets of AONs.

### First level analysis

**Figure 2:**
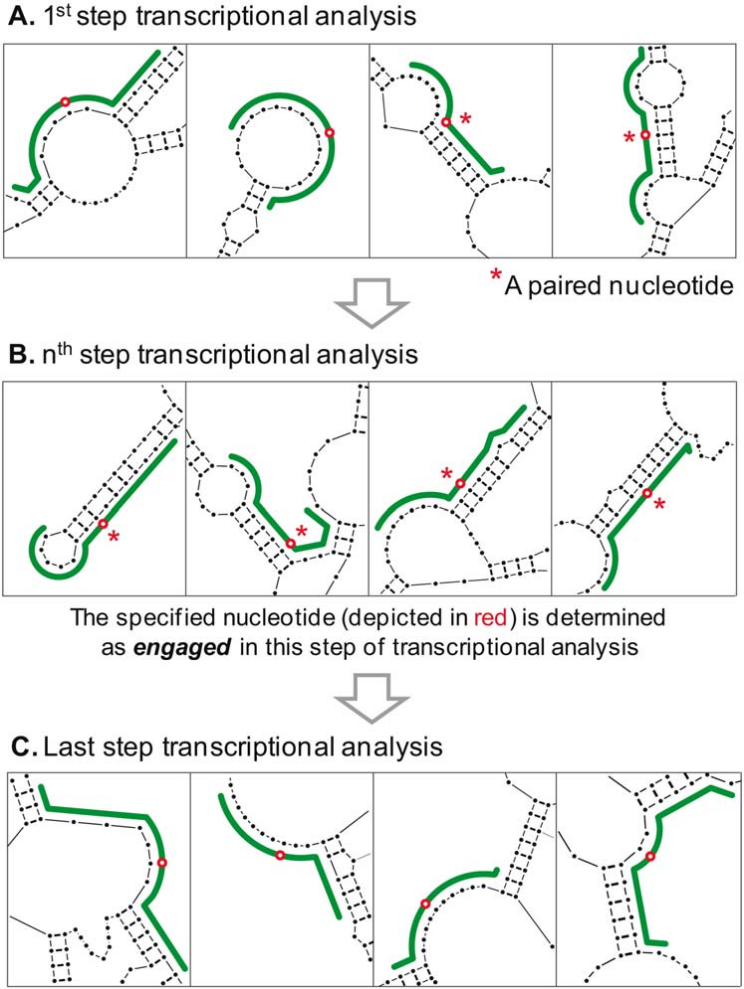
Definition of an *engaged* nucleotide in a particular step of transcriptional analysis. (A) to (C). This is to illustrate that multiple secondary structures of the targeted exon (drawn in green) are predicted in each step of transcriptional analysis, with some of the possible structural motifs shown here. For illustration purpose, a particular nucleotide (marked in red) within an AON target site (green line) is tracked. When this nucleotide is paired (denoted with *), it is not accessible for AON binding. If this nucleotide is paired in all predicted secondary structures, this nucleotide is defined as an *engaged* nucleotide at this particular step of transcriptional analysis (B).

At first level analysis, each nucleotide within the AON target site, a nucleotide accessibility score will be determined by the following ratio:
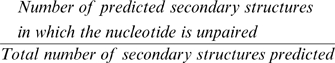
Note that multiple secondary structures will be predicted in each step of transcriptional analysis, see Figure 2. Hence, for each nucleotide, all secondary structures predicted at every step of transcriptional analysis are included. The accessibility score for the AON target site (*L1*) will be:
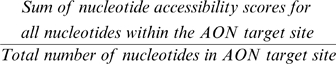
The *L1* scores for the target sites of the analyzed AONs are tabulated in Table S1 of the *Online Supporting Information*. The K-S tests failed to show any statistical difference between *L1* scores for the target sites of Set A AONs of different grades (Table 1A), which agrees with the results reported in refs. [10] and [61]. On the other hand, the *L1* scores for target sites of Set B (++) and (+^1^) AONs were statistically higher that the *L1* scores for target sites of (−) AONs (Table 1B). This result indicates that (++) and (+^1^) AON target sites are more accessible than (−) AON target sites, and therefore, the *L1* score is able to correlate with AON efficacy for Set B AONs. However, as this is not applicable to Set A AONs, we proceeded to the next level of analysis.

**Table 1:** p-values for K-S tests using the first level score (*L1*) and third level score (*L3*) as test variables for differentiating the efficacy and/or efficiency of AONs.

	H_o_:	*L1*		*L3(with outliers)*		*L3(no outliers)*		Test for
		1^st^<2^nd^	1^st^>2^nd^	1^st^<2^nd^	1^st^>2^nd^	1^st^<2^nd^	1^st^>2^nd^	
**A**	**++ vs −**	0.21	0.97	**0.030**	0.81	**0.0044**	1	Efficacy
	**+ vs −**	0.41	0.94	0.92	0.28	0.67	0.51	Efficacy
	**++/+ vs −**	0.42	0.99	0.35	0.49	0.10	0.85	Efficacy
	**++ vs +**	0.44	0.57	**0.0025**	0.82	**0.0014**	0.98	Efficiency
	**++ vs +/−**	0.21	0.85	**0.0035**	0.76	**0.00063**	1	Both
**B**	**++ vs −**	1	**0.032**	**0.032**	0.92	**0.011**	1	Efficacy
	**+ vs −**	0.93	**0.037**	0.060	0.81	**0.035**	0.97	Efficacy
	**++/+ vs −**	0.99	**0.023**	**0.029**	0.87	**0.011**	1	Efficacy
	**+^1^ vs −**	0.92	**0.036**	0.061	0.84	**0.032**	1	Efficacy
	**+^2^ vs −**	0.97	0.076	0.19	0.72	0.17	0.49	Efficacy
	**++ vs +**	0.90	0.44	0.31	0.95	0.14	1	Efficiency
	**++ vs +^1^**	0.90	0.55	0.32	0.96	0.27	0.99	Efficiency
	**++ vs +^2^**	0.68	0.45	0.23	0.77	**0.027**	1	Efficiency
	**+^1^ vs +^2^**	0.61	0.82	0.59	0.61	0.17	0.99	Efficiency
	**++ vs +/−**	0.96	0.38	0.18	0.98	0.057	1	Both

The second column states the two AON grades being tested. ‘/’ means that two grades of AONs are combined into a single grade for the test. In the second row, ‘1^st^’ and ‘2^nd^’ denote the first and second grades being tested (as indicated in the corresponding second column). The last column indicates whether the particular K-S test tests for efficacy and/or efficiency. Significant p-values are highlighted in bold and underlined. Test results for **(A)** AONs in Set A; and **(B)** AONs in Set B.

### Second level analysis

At this level of analysis, the nucleotide accessibility scores of all nucleotides in an AON target site were screened to determine the presence of two or more scores with values below 0.1 occurring consecutively in the nucleotide sequence of the target site (refer to Table S2 of *Online Supporting Information*). Such grouping of below 0.1 nucleotide accessibility scores is termed a “low accessibility cluster”. In Set A, 71% of target sites of (−) AONs had one or more low accessibility cluster(s). While only 17% of target sites of (+) AONs had one or more clusters, they were manifested in 52% of target sites of (++) AONs. Set B also exhibited similar trends: 71%, 70% and 80% of target sites of (−) AONs, (+) AONs and (++) AONs respectively had one or more clusters. Therefore, the presence of these clusters in the AON target sites cannot correlate with AON efficacy and efficiency.

### Third level analysis

**Figure 3:**
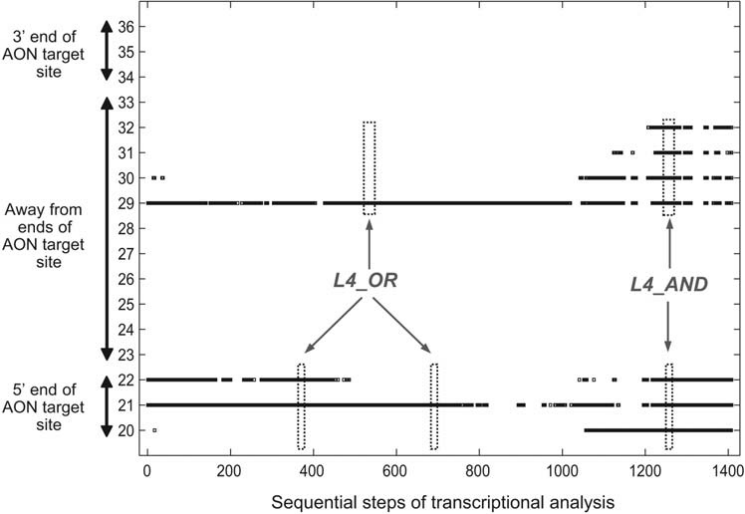
Occurrences of *engaged* nucleotides at each step of transcriptional analysis. In the above illustration, the horizontal axis denotes sequential steps of transcriptional analysis while the vertical axis denotes numbered nucleotides within the AON target site. At each step of transcriptional analysis, nucleotides in the target site that are *engaged* are depicted as a black dot in the plot. The calculations of the fourth level scores, *L4_OR* and *L4_AND*, are illustrated (refer to main text for details).

The nucleotide accessibility scores at the first and second levels of analysis are mean scores. As a result, two nucleotides with identical accessibility scores may have markedly different numbers of unpaired predicted secondary structures at each step of transcriptional analysis. In analyzing accessibility for AON binding, it may be important to take into account steps of transcriptional analysis in which a nucleotide is predicted to have total absence of unpaired secondary structures, i.e. the nucleotide is predicted to be completely inaccessible or “*engaged*” at the particular step of transcriptional analysis (Figure 2B). For the purpose of analysis, at every step of transcriptional analysis, each nucleotide in the AON target site which is *engaged* may then be depicted in a plot as illustrated in Figure 3. Table S3 of the *Online Supporting Information* tabulates these plots for all the AON target sites analyzed.

For each nucleotide in an AON target site, a nucleotide *engaged* score may be derived as follows:
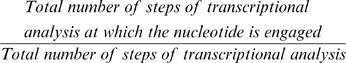
Following this, an AON target site *engaged* score (*L3*) may be derived as follows:
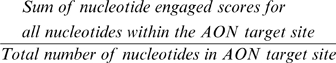
(Table S1 of the *Online Supporting Information* tabulates the *L3* scores for all the AONs analyzed)

For Set A AONs, target sites of (++) AONs had statistically lower *engaged* scores than target sites of both (−) and (+) AONs. Therefore, *L3* score can statistically differentiate both AON efficacy and efficiency (Table 1A). However, seven outlier AONs (6% of the total) were identified. In this context, these were AONs in which the target site *L3* scores contradicted their AON grades. For instance, target sites of h52AON2 and h60AON2 graded as (−) could not induce exon skipping even though their *L3* scores were below the 5^th^-percentile of *L3* scores of (++) AON target sites. On the other hand, target sites of h45AON5 and h46AON4 graded as (+) and target sites of h51AON29, h55AON5 and h77AON2 graded as (++) all had *L3* scores higher than the 95^th^-percentile of *L3* scores of (−) AON target sites but could still induce exon skipping. The omission of these outlier AONs strengthened the correlation of *L3* scores with AON efficacy and efficiency (Table 1A). For Set B AONs, target sites of (++) AONs had statistically lower *engaged* scores than target sites of (−) AONs. Therefore, *L3* scores can statistically differentiate AON efficacy (Table 1B). Similarly, four outlier AONs (6% of the total) were identified, i.e., H30A, H58A, H64A and H34A2. The omission of these outlier AONs enabled *L3* scores to statistically differentiate efficacy between (+^1^) and (−) AONs, and efficiency between (++) and (+^2^) AONs. Remarkably, *L3* scores can differentiate between more AON grades than *L1* scores. Moreover, for all K-S tests in which *L1* scores showed statistical significance, the corresponding K-S tests of *L3* score obtained smaller p-values. Taken together, *L3* scores can differentiate both AON efficacy and efficiency better than *L1* scores.

**Figure 4:**
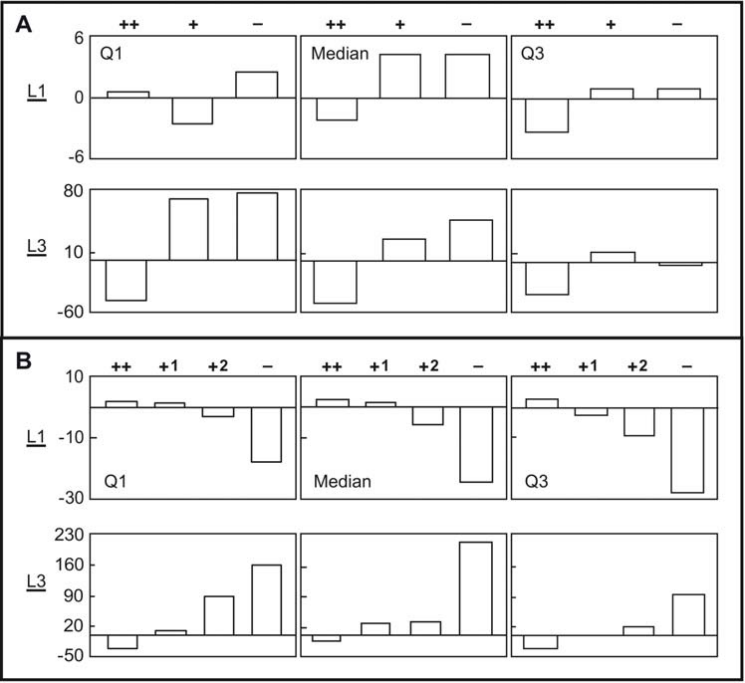
Quartiles of the normalized first and third level scores. The quartiles (Q1, median and Q3) of the normalized first level scores (*L1*), and normalized third level scores (*L3*) for target sites of AONS in every AON grade of (A) Set A and (B) Set B are plotted. The units for all the vertical axes are in percentages.

To appreciate the contrast between K-S test results of the first and third level scores, we plotted the quartiles of the normalized *L1* scores (*L1*) and *L3* scores (*L3*) of AON target sites for AONs in each grade of Sets A and B for comparison (Figure 4A and 4B respectively). For example, the *L1* score of an AON target site from Set A is the relative percentage difference between its *L1* score and the average *L1* score of all AON target sites from Set A. As expected, (++) AON target sites had the lowest *L3* score quartiles in both sets of AONs. Surprisingly, the maximum range for *L3* scores is 7 to 10 times larger than the range for *L1* score. Specifically, for Set A AONs, the range for target site *L3* scores is 140% in contrast to 12% for *L1* scores; for Set B AONs, the range for target site *L3* scores is 280% in contrast to 40% for *L1* score.

Overall, for Set B AONs, the *L3* scores can satisfactorily differentiate efficacies and efficiencies of (++), (+) and (+^1^) AONs. On the other hand, for Set A AONs, while *L3* scores can differentiate (++) AONs from the rest of AON grades, they cannot account for the efficacies of (+) AONs because both (+) and (−) AON target sites had statistically similar *L3* scores. Intriguingly, the p-value for (++) vs. (+) AONs was even smaller than for (++) vs. (−) AONs. This indicates that although (+) AON target sites had high *L3* scores, they can still induce exon skipping albeit not efficiently. Hence, a more detail analysis was needed.

### Fourth level analysis

While third level analysis primarily involves a general measure of frequency of *engaged* nucleotides, fourth level analysis includes consideration of localization of consecutive *engaged* nucleotides in the sequence of steps of transcriptional analysis of an AON target site. Three fourth level scores were developed for this analysis. These scores were applied to groups of 2 to 5 consecutive nucleotides in the AON target site and correlated with AON efficacy and efficiency (see *Methods and Materials* for details).

1. *L4_AVG*–
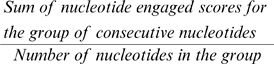

2. *L4_AND*–
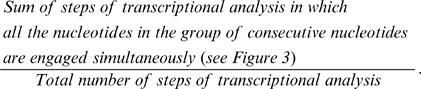

3. *L4_OR*–
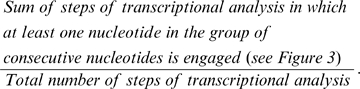

Preliminary analysis showed that the presence of consecutive *engaged* nucleotides at the ends of an AON target site attenuated AON efficacy and efficiency more than at other sites (data not shown). With this insight and to increase the power of the statistical tests, we analysed the localization of consecutive *engaged* nucleotides at the ends and away from the ends of the AON target site separately.

### Engaged nucleotides away from the ends of an AON target site

For the purpose of this analysis, “away from the ends of an AON target site” refers to nucleotides in the target site that are at least four nucleotides away from both 3′ and 5′ ends, as illustrated in Figure 3. We extracted groups of consecutive nucleotides consisting of two to five nucleotides from every AON target site analyzed. The three fourth level scores were calculated only for those groups of consecutive nucleotides meeting the following criterion for analysis: every nucleotide in the group having an *engaged* score of at least 0.1. Subsequently, statistical tests as in Table 1 were applied to the scores. The analyses were stratified according to the number of consecutive nucleotides in the groups scored, as described below.

#### Groups of two consecutive nucleotides

For both Set A and Set B AONs, the K-S tests found no statistical differences in all three scores of AON target sites at the different AON grades (data not shown). Note: inadequate AON sample size in Set B restricted the tests to scores of target sites of (++) vs. (+^1^) AONs and (++) vs. (+/−) AONs.

#### Groups of three consecutive nucleotides

**Figure 5:**
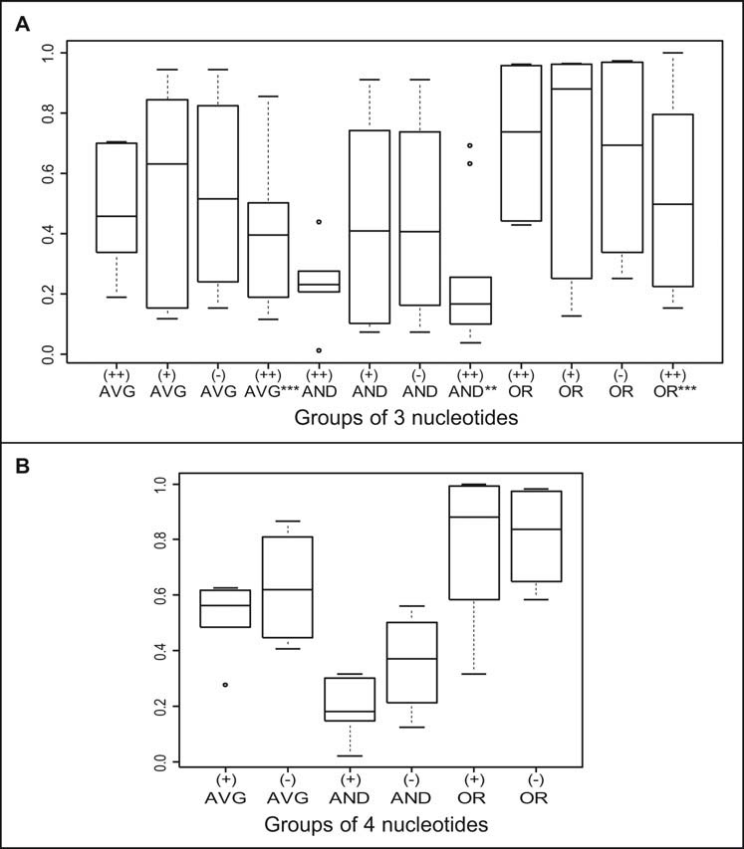
Box-plots for each of the fourth level scores *L4_AVG, L4_AND* and *L4_OR.* (A) Box-plots for scores of groups of 3 nucleotides meeting criterion of analysis in target sites of (++) AONs, (+) AONs and (−) AONs of Set A, and of (++) AONs of Set B (***). (B) Box-plots for scores of groups of 4 nucleotides meeting criterion of analysis in target sites of both (+) and (−) AONs of Set A.

K-S tests could not be performed for both sets, as AON sample sizes of many AON grades were not adequate (<6) to confer statistical confidence. Nevertheless, for Set A AONs, box-plots for each score were constructed in Figure 5A. The *L4_AND* score can differentiate (++) AONs from the other two AON grades comparatively well. While the *L4_AVG* score displayed some ability to differentiate (++) AONs, the *L4_OR* score failed to do so. For Set B AONs, only (++) AONs had adequate sample size to construct the box-plot (Figure 5A). Consistent with the results for Set A AONs, the *L4_AND* score of (++) Set B AON target sites had the smallest median and inter-quartile range compared to the other two scores.

#### Groups of four consecutive nucleotides

For Set A AONs, only (+) and (−) AONs had adequate sample sizes to construct the box-plots (Figure 5B). Again, the *L4_AND* score demonstrated the best ability to differentiate (+) AONs from (−) AONs while the *L4_OR* score failed to do so. The sample sizes of Set B AONs at all grades were too small for analysis.

#### Groups of five consecutive nucleotides

For Set A AONs, while three target sites of (−) AONs were found to have such a group as defined for this analysis, it was not found in all target sites of both (+) and (++) AONs. For Set B AONs, such a group was found in 33%, 11% and 6% of target sites of (−) AONs, (+) AONs and (++) AONs respectively.

Taken together, these results suggested that the presence of at least three consecutive *engaged* nucleotides at simultaneous steps of transcriptional analysis but not the individual nucleotide *engaged* score attenuated AON efficacy and efficiency.

### Engaged nucleotides at the ends of an AON target site

“At the ends of an AON target site” refers to nucleotides in the target site that are within three bases at 3′ or 5′ ends. For every AON target site analyzed, the three fourth level scores were calculated for every group of three consecutive nucleotides at 3′ and 5′ ends of the target site. Groups with zero L4_AVG score, i.e., all the nucleotides were not *engaged* at any step of transcriptional analysis, were excluded from the statistical tests. Statistical tests as in Table 1 were applied to each of the three fourth level scores. Significant p-values of the K-S tests for target site scores of Sets A and B AONs are tabulated in Table 2A.

**Table 2:** p-values for K-S tests using the fourth level scores as test variables.

	Set	H_o_:	*L4_AVG*		*L4_AND*		*L4_OR*		Test for
			1^st^<2^nd^	1^st^>2^nd^	1^st^<2^nd^	1^st^>2^nd^	1^st^<2^nd^	1^st^>2^nd^	
**A**	**A**	**++ vs −**	**0.0089**	0.97	**0.028**	0.97	**0.0019**	0.96	Efficacy
	**A**	**++/+ vs −**	0.055	0.96	**0.039**	0.97	**0.014**	0.96	Efficacy
	**A**	**++ vs +/−**	**0.013**	0.96	0.093	0.96	**0.0035**	0.96	Both
	**B**	**++ vs +^1^**	0.17	0.98	**0.036**	0.98	0.31	0.93	Efficiency
	**B**	**++ vs +/−**	**0.025**	0.95	0.11	0.97	0.082	1	Both
**B**	**A**	**++ vs −**	**0.017**	0.91	0.055	0.91	**0.017**	0.82	Efficacy
	**A**	**++ vs +/−**	**0.012**	0.94	0.17	0.66	**0.020**	0.86	Both
**C**	**A**	**++ vs −**	0.11	0.96	0.088	0.96	**0.026**	0.96	Efficacy
	**A**	**++/+ vs −**	0.16	0.96	0.11	0.99	**0.047**	0.94	Efficacy
	**B**	**++ vs +/−**	**0.018**	0.96	0.25	1	**0.040**	0.96	Both
**D**	**A**	**3′ vs 5′ (++)**	**0.028**	0.79	0.24	0.44	0.060	0.89	Efficiency

Only tests with significant p-values were shown. **(A)** Test data includes both 3′ and 5′ ends of AON target sites. **(B)** Test data consists of only 3′ end of AON target sites. **(C)** Test data consists of only 5′ end of AON target sites. **(D)** 3′ vs. 5′ end of AON target sites.

For Set A AONs, the *L4_OR* scores demonstrated the best ability to differentiate AON efficacy and efficiency, followed by the *L4_AVG* and *L4_AND* scores. In contrast, the *L4_AVG* score demonstrated the best ability to differentiate Set B (++) AONs, followed by the *L4_AND* score but not the *L4_OR* score. As the *L4_AND* score did not show the best ability to correlate AON efficacy and efficiency in both sets, AON efficacy and efficiency is more attenuated by presence of *engaged* nucleotides at the ends of target sites than at other sites. In addition, given that the *L4_OR* score only counted steps of transcriptional analysis in which at least one nucleotide was *engaged*, the test results suggested that efficacy and efficiency of shorter AONs (Set A) was more attenuated by *engaged* nucleotides at the ends of their target sites compared to longer AONs (Set B). To investigate whether *engaged* nucleotides at either 3′ or 5′ end of target sites affected AON efficacy and efficiency differently, we stratified the groups into 3′ and 5′, and repeated the same K-S tests, as discussed below.

#### Engaged nucleotides at 3′ end

(Table 2B) For Set A AONs, the K-S test results were consistent with those obtained from both ends of AON target sites (Table 2A) except that, the *L4_AND* scores now failed to differentiate any AON grade. For Set B AONs, small sample sizes only permitted testing between target sites of (++) vs. (+^1^) AONs and no statistical difference was obtained for all three scores (data not shown).

#### Engaged nucleotides at 5′ end

(Table 2C) For Set A AONs, only the *L4_OR* scores can differentiate AON efficacy. For Set B, small sample sizes only permitted testing between target sites of (++) AONs vs. (+^1^), (+) and (+/−) AONs. Although both the *L4_AVG* and *L4_OR* scores can differentiate (++) AONs from (+/−) AONs, the *L4_AVG* scores had a smaller p-value. This plausibly suggests that although *engaged* nucleotides at 5′ end also attenuated the efficacy and efficiency of Set B AONs, the extent of attenuation was less marked than Set A AONs. Altogether, these test results (Tables 2A–2C) strongly support the conclusion that *engaged* nucleotides at the ends of a shorter target site attenuated AON efficacy and efficiency more markedly than a longer target site.

#### Engaged nucleotides at 3′ end vs. engaged nucleotides at 5′ end

Here, we tested whether there was statistical difference in the localization of *engaged* nucleotides in the sequence of steps of transcriptional analysis between 3′ and 5′ ends of target sites of AONs of the same grade. For instance, in the case of (++) AONs, each of the fourth level scores of the groups of nucleotides at the target site 3′ end was compared with the 5′ end using K-S tests. For Set A AONs, the groups of nucleotides at the 3′ end of (++) AON target sites had statistically smaller *L4_AVG* scores than at the 5′ end (Table 2D). This implied that *engaged* nucleotides at 3′ end of a short target site attenuated AON efficiency more than at 5′ end. For Set B AONs, on the other hand, small sample sizes only permitted testing of target sites of (++) AONs and (+) AONs but no statistical difference was obtained (data not shown).

## Discussion

Previous studies have supported the general principle that mRNA secondary structures influence AON efficacy and efficiency [62]–[64], although these studies did not consider *co-transcriptional* folding in the prediction of the secondary structures. Furthermore, laboratories working this field [10], [61] have reported no correlation with secondary mRNA structure in designing AONs to induce exon skipping of the dystrophin gene. In these reports, *co-transcriptional* dynamic changes in secondary structure were either not considered or were approximated with a relatively unrefined methodology. Our study aims to re-visit this issue by using a more refined method to approximate *co-transcriptional* dynamic changes in pre-mRNA secondary structures and by developing novel methods to take into account the localization of completely inaccessible nucleotides in the *co-transcriptional* process. Applying four levels of analysis with scoring methodologies of increasing complexity, we demonstrate that the frequency and localization of consecutive *engaged* nucleotides in the sequence of steps of transcriptional analysis correlated with efficacy and efficiency of 94% of previously reported AONs.

Four key novel insights pertaining to AON efficacy and efficiency were deduced from this study. Firstly, the lowest frequencies of *engaged* nucleotides manifested at target sites were associated with the most efficient (++) AONs. Secondly, *engaged* nucleotides at 3′ or 5′ ends of the target site attenuated AON efficacy and efficiency more than at other sites. Thirdly, the efficacy and efficiency of longer AONs were less attenuated by *engaged* nucleotides at 3′ or 5′ ends of the target site as compared to shorter AONs. In fact, the frequency and localization of *engaged* nucleotides of short AON (Set A) and long AON (Set B) target sites were statistically similar (see Table S4 of the *Online Supporting Information* for more details). In agreement with reported studies [61], these results indicate that AONs targeting longer target sites can induce exon skipping more effectively and efficiently by possibly binding to the pre-mRNA more stably as compared to AONs targeting shorter target sites. Fourthly, *engaged* nucleotides at 3′ end of a short target site attenuated AON efficiency more than at 5′ end. This might explain why AON efficiency is more sensitive to nucleotide changes at the 3′ end than 5′ end of its target site [65]. Notably, our results provide quantitative statistical proof for these experimental observations.

**Figure 6:**
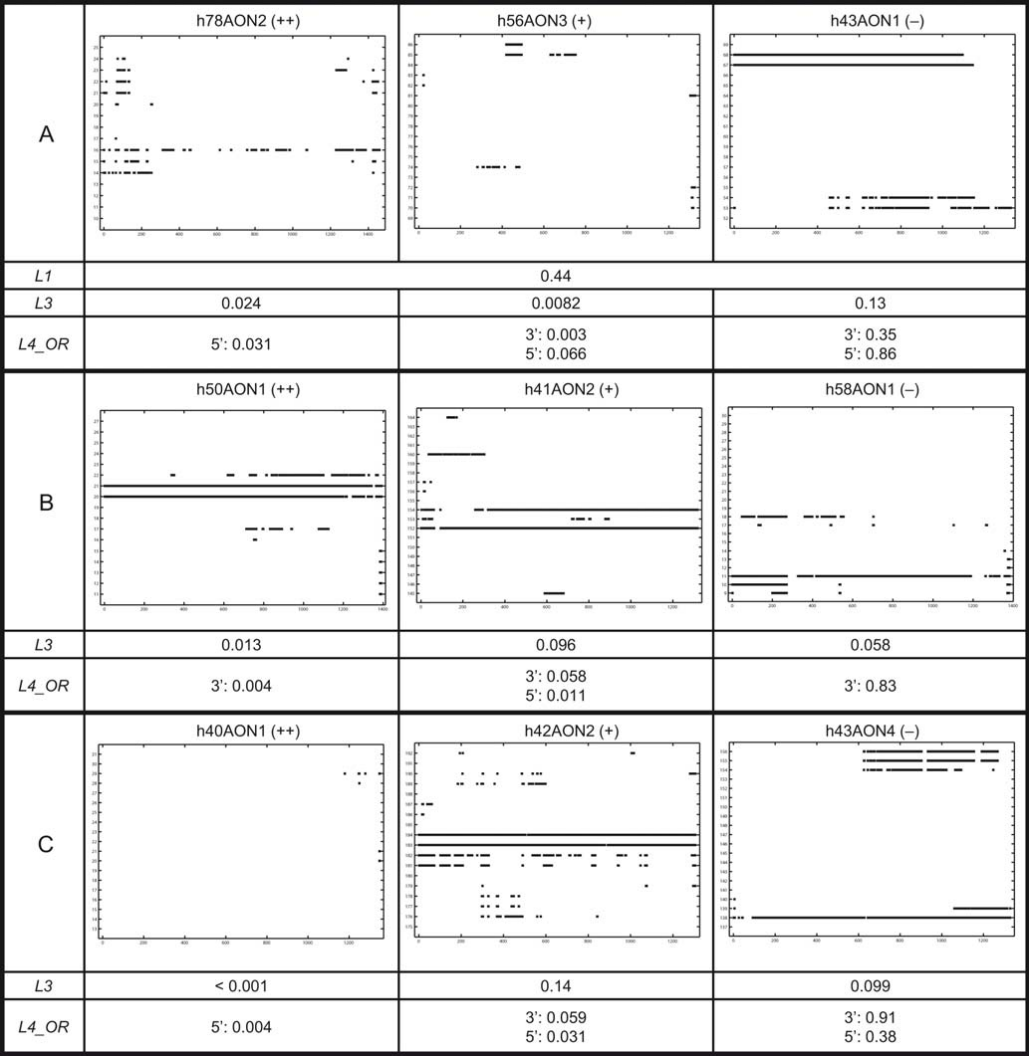
Three examples for where only the fourth level scores can correlate AON efficacy and efficiency compared to third level scores. (A) to (C) For every example, the incidences of *engaged* nucleotides at each step of transcriptional analysis for all nucleotide in target sites of (++), (+) and (−) AONs were depicted.

To demonstrate the correlative power of the fourth level scores, three common examples in which only the fourth level scores can differentiate (++) AONs in Set A (Figure 6) are discussed. Figure 6A illustrated an example wherein AON target sites with identical accessibility scores (*L1*) can have strikingly different *engaged* scores (*L3*). Whereas the (−) AON target site high *engaged* score was expected, the higher *engaged* score of the (++) AON target site compared to the (+) AON target site was confounding. In contrast to the *L3* score, fourth level analysis showed more *engaged* nucleotides at the ends of (+) AON target site than at (++) AON target site, i.e. was able to discriminate between (+) and (++) AON target sites. Figure 6B illustrated an example in which the target site *engaged* scores (*L3*) correlate inversely with AON efficacy and efficiency, i.e., AON target sites with higher *engaged* scores had better ability to induce exon skipping. Again, the fourth level scores resolved this conundrum in a similar manner as the first example. The final example (Figure 6C) illustrated a widespread phenomenon in the data set in which (+) AON target sites had higher *engaged* scores (*L3*) than (−) AON target sites. In fact, this phenomenon caused the p-values of K-S tests of (++) vs. (+) AON target site *L3* scores to be smaller than for (++) vs. (−) target site *L3* scores. In most instances, most of the *engaged* nucleotides manifested in (+) AON target sites were localized away from the ends of the sites. Altogether, these examples showed that localization is as important as the frequency of *engaged* nucleotides.

**Figure 7:**
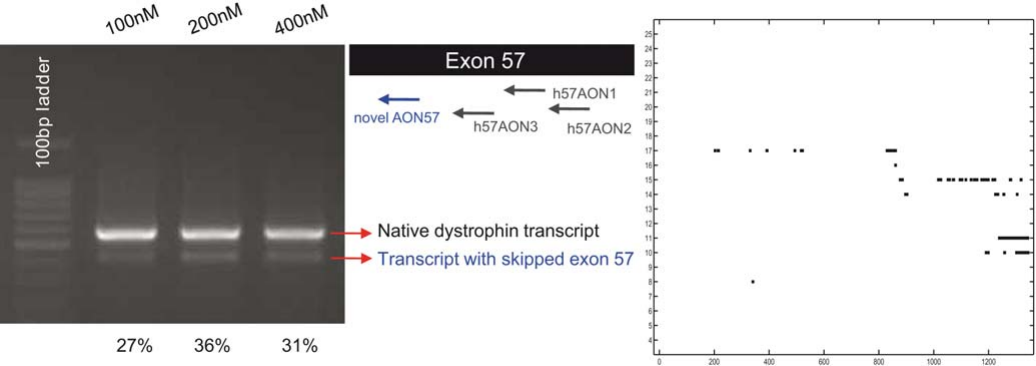
The efficiency of a novel AON targeting exon 57. The left panel shows the RT-PCR analysis of dystrophin mRNA treated with novelAON57 at concentrations of 100nM, 200nM and 400nM of AONs. For every AON concentration, the relative percentage of total transcripts (average value of duplicate transfections) with skipping of the targeted exon is given below the lanes. The middle panel indicates the relative position of the novel AONs with respect to published AONs. The right panel plots the *co-transcriptional* binding accessibility of the novel AON target sites, wherein the horizontal axis denotes sequential steps of transcriptional analysis and the vertical axis denotes numbered nucleotides within the AON target site. At each step of transcriptional analysis, nucleotides in the target site that are *engaged* are depicted as a black dot in the plot.

As an illustration, an AON (novelAON57) target sequence was selected to skip exon 57. All three reported AONs (h57AON1, h57AON2, h57AON3) designed to induce exon 57 skipping by targeting the intra-exonic sequences failed to skip exon 57 [10]. Interestingly, exon 57 manifests an overwhelming occurrence of *engaged* nucleotides (Figure S3 of the *Online Supporting Information*); hence, it is relatively difficult to locate a suitably long sequence that has ESE activity as well as *co-transcriptional* binding accessibility that fulfils the four insights (as described above). For instance, the 3′ ends of the target sites of both h57AON1 and h57AON2 AONs manifest substantial *engaged* nucleotides whereas the first half of the target site of h57AON3 AON manifests extensive *engaged* nucleotides (Table S3 of the *Online Supporting Information*). We designed novelAON57 to have a target site with the following characteristics: negligible occurrence of *engaged* nucleotides, presence of ESE motifs predicted by ESE-Finder [66] and RESCUE-ESE [67], and location at the first half of the exon. Notably, novelAON57 targets a completely different site from the published AONs, as shown in Figure 7. At all AON concentrations tested, i.e. 100nM, 200nM, and 400nM, novelAON57 demonstrates selective skipping of exon 57 with an efficiency of (++) (Figure 7).

The number of secondary structures predicted for each exon was tabulated in Table S5 of the *Online Supporting Information*. There was an average of 44,582 predicted secondary structures per exon and 24 to 47 predicted secondary structures per step of transcriptional analysis (Table S5). As a result, the identification of *engaged* nucleotides at a step of transcriptional analysis had low false-positives, as an *engaged* nucleotide must be paired in all predicted secondary structures. In addition, the use of numerous windows of analysis had the added advantage of spreading out the prediction error of mfold as vast numbers of secondary structures were used in the analyses. Despite the exceptional length of the dystrophin gene, the measured average elongation rate does not differ significantly from other genes [45]. While this seems to suggest that transcription of dystrophin gene is similar to other genes, the possibility of other transcription and/or splicing factors being involved cannot be dismissed. In the event that such factors significantly affect the rate of transcript elongation and/or the mechanism of exon recognition, the results of our analyses might differ substantially.

Besides *co-transcriptional* binding accessibilities of AON targets, AON efficiency depends on other factors such as presence of ESEs, stability of AONs (by chemical modifications), thermodynamic considerations, absolute distance of AON target site from 3′ splice site, etc. For instance, statistical analysis of predicted ESE sites by Aartsma-Rus *et al* [10] showed that target sites of (++) AONs in Set A had marginally more active ESE sites (p-value≈0.05). Therefore, the prediction of efficient AON targets does not simply involve annealing of an AON to a structurally accessible target as the best *co-transcriptionally* accessible target site might not contain ESEs. As these factors are often not mutually exclusive, an AON target site that fares very well in one factor but poorly in others might not be efficient.

The development of scores at four levels of analysis to semi-quantify co-transcriptional binding accessibility of AON target sites allows their correlation with AON efficacy and efficiency using statistical tests. These methodologies are potentially applicable to the development of a systematic approach to identify optimal target sites in the design of AONs to induce exon skipping of dystrophin pre-mRNA. Similarly, the methodologies may also be applicable in analyzing the efficiency of AONs applied in other diseases, such as thalassemia [14]–[17], ocular albinism [18] and cancer [19], in which exon splicing modulation to correct the mRNA reading frame has been proposed as a therapeutic strategy.

## Materials and Methods

### Data Set

Set A is extracted from the list of 114 AONs published by Aartsma-Rus *et al.* [10]. Among them, 41 of them induce exon skipping in >25% of the total dystrophin mRNA transcripts and are graded as (++); 35 of them induce exon skipping in <25% of the total transcripts and are graded as (+); and 38 of them fail to induce exon skipping and are graded as (−). On the other hand, Set B is extracted from the list of AONs published by Wilton *et al.* [6]. Although they published a total of 82 AONs, only 62 of them are applicable for this study. The remaining ones either target non-ESE sites or result in unspecific exon skipping. Among the relevant AONs, 35 of them induce exon skipping in >30% of the total dystrophin mRNA transcripts and are graded as Type 1; 11 of them induce exon skipping in between 10% to 30% of the total transcripts and are graded as Type 2; 9 of them induce exon skipping in <10% of the total transcripts and are graded as Type 3; and 7 of them fail to induce exon skipping (i.e., non-effective) and are graded as Type 4. For naming consistency, Types 1–4 are renamed as grades (++), (+^1^), (+^2^) and (−) respectively. Altogether, the 176 AONs target 67 exons in the dystrophin gene by blocking ESEs. AON efficacy was determined from RT-PCR analysis while AON efficiency was calculated based on densitograph semi-quantification [6], [10].

### Computational prediction of the dynamical pre-mRNA secondary structure

The methodology for quantifying and analyzing the dynamics of the pre-mRNA structures in the progression of transcription did not depend on the choice of the prediction tool as long as *co-transcriptional* structures were obtainable. mfold was eventually chosen because of its relative efficiency for computing long RNAs as well as the advantage of being used in most published experimental results on AONs that target dystrophin gene [9], [10], [68] and therefore, the results in this work can be compared with them on a common basis. mfold version 3.1 [28], [69] was executed on a Dell PowerEdge SC1420 server running Red Hat Enterprise Linux 4.0 OS. Since it was highly probable that the nascent pre-mRNA may not have the chance to assume optimal structures, we accepted sub-optimal structures whose energies lie within 5% of the optimum.

Since long introns are typical in dystrophin gene, only local secondary structures around the targeted exon need to be considered. This was because abundant hnRNPs (heterogeneous nuclear ribonuclear proteins) package long intron regions into compact and manageable secondary structures for pre-mRNA processing that deterred long-distance or global intra-molecular complementary base pairings, which possibly prevented an exon from being entangled in a complex structure that would obstruct the spliceosome from accessing it [70]. On the other hand, sequence length of the window of analysis was estimated from experimental measurements: elongation rate of dystrophin mRNA ranged from 1.7 to 2.5 kb per minute [45]; and RNP formation at 3′ splice sites was observed 48 seconds after 3′ splice sites synthesis [71]. During the time-delay from 3′ splice site synthesis to its recognition, about 1360 to 2000 bases were appended to the nascent transcript. The dynamical secondary structures of exons 2 (62 bp), 29 (150 bp) and 59 (269 bp) were predicted based on 1200, 1500 and 2000 sequence length of the window of analysis. For each exon, there was no statistical difference in the nucleotide accessibility and *engaged* scores computed from secondary structures predicted based on different sequence lengths of window of analysis (data not shown). Therefore, the predicted secondary structures of a target exon were not sensitive to sequence length of the window of analysis.

### Statistical test for differentiating AON efficacy and efficiency

Two-sample Kolmogorov-Smirnov (K-S) test was used to test for statistical differences and significances of the first, third and fourth level scores in their abilities to differentiate AON efficacy and efficiency between any two AON samples. Both two-tailed (H_o_: the two AON samples have different probability distributions) and one-tailed (H_o_: the first AON sample is larger/smaller than the second AON sample) tests were performed to ensure consistency of test results. All statistical tests were performed on the statistical software, R Version 2.0.0 [72]. Note: Wilcoxon rank-sum test was not used because box-plots of two AON samples showed that they had different distributional shapes (data not shown), which violated a key Wilcoxon test assumption.

### Preliminary analysis in the fourth level analysis

In this preliminary analysis, the localization of groups of consecutive *engaged* nucleotides in the sequence of steps of transcriptional analysis of an AON target site was tested for correlation with AON efficacy and efficiency. For each AON analyzed, all possible groups of consecutive nucleotides in the AON target site were obtained. For instance, groups of two consecutive nucleotides were extracted by walking one nucleotide at a time from one end of an AON target site to the other end. Likewise, groups of three to five consecutive nucleotides are obtained similarly. The three fourth level scores (*L4_AVG*, *L4_AND* and *L4_OR*) were next applied on every group of consecutive nucleotides. Their scores were then correlated with AON efficacy and efficiency by K-S tests among various AON grades in Sets A and B. As the number of nucleotides in the groups may influence AON efficacy and efficiency, the K-S tests were stratified according to the numbers of nucleotides in the groups. Note: as the majority of the groups with more than five consecutive nucleotides have zero *L4_AND* scores, inadequate sample size constrained the analysis to a maximum of five consecutive nucleotides.

### Illustration of the efficiency of a novel AON targeting exon 57

A novel AON (novelAON57) was synthesized by Sigma-Prologo (France) with 2′-O-methyl and full length phosphorothioate backbones according to our specifications. Transfections were done on normal human fibroblast cells (Coriell, USA) cultured in 6-wells plates, with the AON concentrations of 100 nM, 200 nM or 400 nM and LipofectAmine 2000, with ratio of concentrations as suggested by the manufacturer (Invitrogen, Carlsbad, Canada). The transfection was done in duplicate. 24 hours after transfection, the cells were harvested and subjected to mRNA analysis to assess the performance of the AONs in inducing exon skipping. Total RNA was isolated using Trizol (Invitrogen, Carlsbad, Canada). Single step RT-PCR was performed on ∼400ng total RNA using a single step RT-PCR analysis kit, Access RT-PCR system (Promega, Madison, USA), according to the manufacturer's instructions for 20 cycles, followed by nested PCR for 22 cycles. Sequences of dystrophin exon-specific primers used for single step RT-PCR and nested PCR are available upon request. Exon skipping efficiency was estimated by densitometry analysis of the gel images comparing the density of amplicons from dystrophin mRNA with exon 57 skipping to the native dystrophin mRNA.

## Supporting Information

Figure S1 Genomic lengths of all exons and introns of dystrophin gene. The sequence lengths for each of the 79 exons in dystrophin are plotted as black bars. For every exon, both of their flanking introns sequence lengths are shown as gray bars. Note that the sequence length is on a logarithmic scale (vertical axis). Total exonic and intronic sequence length are 11,034 bps and 2,209,348 respectively. The exons occupied a mere 0.5% of its full DNA sequence. The lengths of the exons range from 7 to 269 bps; introns range from 107 to 319,058 bps.
(0.06 MB DOC)

Figure S2 Percentage genomic lengths of each exon relative to the total length of its flanking introns. To underscore the fact that locating an exon in dystrophin is akin to finding a needle in a haystack, the percentage of the length of an exon relative to the total length of its 3′ and 5′ intron sequences is computed and is plotted here. The majority of the exons constitute less than 1% of their intronic lengths and even the highest percentage is less than 7%.
(0.06 MB DOC)

Figure S3 Co-transcriptional binding accessibilities of exon 57. The horizontal axis denotes sequential steps of transcriptional analysis whereas the vertical axis denotes numbered nucleotides within the AON target site. At each step of transcriptional analysis, nucleotides in the target site that are engaged are depicted as a black dot in the plot.
(0.08 MB DOC)

Table S1 First level score (*L1*) and third level score (*L3*) of 176 AON target sites analysed. This table tabulates the first level score (*L1*) and the third level score (*L3*). The AONs are sorted in ascending order of their target exon number, where the exon number is indicated in the AON names after the letter ‘h’, for e.g. h2AON1 targets exon 2. The sources of the AONs are indicated as superscripts on their names.
(0.20 MB DOC)

Table S2 The nucleotide accessibility score of all nucleotide in an AON target site is plotted for all the 176 AONs analysed. The horizontal axis represents the nucleotide position in the respective target exon and the nucleotide accessibility score is plotted on the vertical axis.
(0.48 MB DOC)

Table S3 Occurrences of engaged nucleotides at each step of transcriptional analysis for all nucleotide in an AON target site are depicted for each of the 176 AON target sites analysed. The horizontal axis denotes sequential steps of transcriptional analysis while the vertical axis denotes numbered nucleotides within the AON target site. At each step of transcriptional analysis, nucleotides in the target site that are engaged are depicted as a black dot in the plot.
(1.93 MB DOC)

Table S4 p-values for K-S tests using the third level score (*L3*) and fourth level scores (*L4_AVG*, *L4_AND* and *L4_OR*) as test variables between Set A and Set B. (A) (++) and (+) AONs are tested between Set A and Set B for statistical difference using L3 as test variable. (B) to (E) Fourth level scores as test variables for engaged nucleotides localized at (B) both 3′ and 5′ ends, (C) at 3′ end, (D) at 5′ end and (E) away from the ends of the AON target sites Note: (-) AONs between the two sets cannot be tested because the sample size in Set B is too small to confer statistical confidence.
(0.07 MB DOC)

Table S5 Number of predicted secondary structures generated in each exon. For every exon in dystrophin gene, the total number of secondary structures predicted as well as the average number of predicted secondary structures per step of transcriptional analysis is tabulated.
(0.12 MB DOC)

## Acknowledgments

## References

[1] Wilton SD, Fletcher S (2005). Antisense oligonucleotides in the treatment of Duchenne muscular dystrophy: Where are we now?. Neuromuscul Disord.

[2] Wilton SD, Fletcher S (2005). RNA splicing manipulation: strategies to modify gene expression for a variety of therapeutic outcomes.. Curr Gene Ther.

[3] Matsuo M, Takeshima Y (2005). Rescue of dystrophin mRNA of Duchenne muscular dystrophy by inducing exon skipping.. Acta Myol.

[4] van Deutekom JC, van Ommen GJ (2003). Advances in Duchenne muscular dystrophy gene therapy.. Nat Rev Genet.

[5] Matsuo M (2002). Duchenne and Becker muscular dystrophy: from gene diagnosis to molecular therapy.. IUBMB Life.

[6] Wilton SD, Fall AM, Harding PL, McClorey G, Coleman C (2007). Antisense Oligonucleotide-induced Exon Skipping Across the Human Dystrophin Gene Transcript.. Mol Ther.

[7] Aartsma-Rus A, Kaman WE, Weij R, den Dunnen JT, van Ommen GJ (2006). Exploring the frontiers of therapeutic exon skipping for Duchenne muscular dystrophy by double targeting within one or multiple exons.. Mol Ther.

[8] Aartsma-Rus A, Janson AA, Kaman WE, Bremmer-Bout M, van Ommen GJ (2004). Antisense-induced multiexon skipping for Duchenne muscular dystrophy makes more sense.. Am J Hum Genet.

[9] Aartsma-Rus A, Bremmer-Bout M, Janson AA, den Dunnen JT, van Ommen GJ (2002). Targeted exon skipping as a potential gene correction therapy for Duchenne muscular dystrophy.. Neuromuscul Disord.

[10] Aartsma-Rus A, De Winter CL, Janson AA, Kaman WE, van Ommen GJ (2005). Functional analysis of 114 exon-internal AONs for targeted DMD exon skipping: indication for steric hindrance of SR protein binding sites.. Oligonucleotides.

[11] Matsuo M (1996). Duchenne/Becker muscular dystrophy: from molecular diagnosis to gene therapy.. Brain Dev.

[12] Pramono ZA, Takeshima Y, Alimsardjono H, Ishii A, Takeda S (1996). Induction of exon skipping of the dystrophin transcript in lymphoblastoid cells by transfecting an antisense oligodeoxynucleotide complementary to an exon recognition sequence.. Biochem Biophys Res Commun.

[13] Surono A, Van Khanh T, Takeshima Y, Wada H, Yagi M (2004). Chimeric RNA/ethylene-bridged nucleic acids promote dystrophin expression in myocytes of duchenne muscular dystrophy by inducing skipping of the nonsense mutation-encoding exon.. Hum Gene Ther.

[14] Suwanmanee T, Sierakowska H, Fucharoen S, Kole R (2002). Repair of a splicing defect in erythroid cells from patients with beta-thalassemia/HbE disorder.. Mol Ther.

[15] Gorman L, Mercatante DR, Kole R (2000). Restoration of correct splicing of thalassemic beta-globin pre-mRNA by modified U1 snRNAs.. J Biol Chem.

[16] Sazani P, Kole R (2003). Therapeutic potential of antisense oligonucleotides as modulators of alternative splicing.. J Clin Invest.

[17] Dominski Z, Kole R (1993). Restoration of correct splicing in thalassemic pre-mRNA by antisense oligonucleotides.. Proc Natl Acad Sci U S A.

[18] Vetrini F, Tammaro R, Bondanza S, Surace EM, Auricchio A (2006). Aberrant splicing in the ocular albinism type 1 gene (OA1/GPR143) is corrected in vitro by morpholino antisense oligonucleotides.. Hum Mutat.

[19] Mercatante DR, Sazani P, Kole R (2001). Modification of alternative splicing by antisense oligonucleotides as a potential chemotherapy for cancer and other diseases.. Curr Cancer Drug Targets.

[20] McClorey G, Fall AM, Moulton HM, Iversen PL, Rasko JE (2006). Induced dystrophin exon skipping in human muscle explants.. Neuromuscul Disord.

[21] McClorey G, Moulton HM, Iversen PL, Fletcher S, Wilton SD (2006). Antisense oligonucleotide-induced exon skipping restores dystrophin expression in vitro in a canine model of DMD.. Gene Ther.

[22] Wells DJ (2006). Therapeutic restoration of dystrophin expression in Duchenne muscular dystrophy.. J Muscle Res Cell Motil.

[23] Alter J, Lou F, Rabinowitz A, Yin H, Rosenfeld J (2006). Systemic delivery of morpholino oligonucleotide restores dystrophin expression bodywide and improves dystrophic pathology.. Nat Med.

[24] Fall AM, Johnsen R, Honeyman K, Iversen P, Fletcher S (2006). Induction of revertant fibres in the mdx mouse using antisense oligonucleotides.. Genet Vaccines Ther.

[25] Lu QL, Rabinowitz A, Chen YC, Yokota T, Yin H (2005). Systemic delivery of antisense oligoribonucleotide restores dystrophin expression in body-wide skeletal muscles.. Proc Natl Acad Sci U S A.

[26] Takeshima Y, Yagi M, Wada H, Ishibashi K, Nishiyama A (2006). Intravenous infusion of an antisense oligonucleotide results in exon skipping in muscle dystrophin mRNA of Duchenne muscular dystrophy.. Pediatr Res.

[27] Muntoni F, Bushby K, van Ommen G (2005). 128th ENMC International Workshop on ‘Preclinical optimization and Phase I/II Clinical Trials Using Antisense Oligonucleotides in Duchenne Muscular Dystrophy’ 22-24 October 2004, Naarden, The Netherlands.. Neuromuscul Disord.

[28] Zuker M (2003). Mfold web server for nucleic acid folding and hybridization prediction.. Nucleic Acids Res.

[29] Knudsen B, Hein J (2003). Pfold: RNA secondary structure prediction using stochastic context-free grammars.. Nucleic Acids Res.

[30] Ding Y, Lawrence CE (2003). A statistical sampling algorithm for RNA secondary structure prediction.. Nucleic Acids Res.

[31] Flamm C, Fontana W, Hofacker IL, Schuster P (2000). RNA folding at elementary step resolution.. RNA.

[32] Chalk AM, Sonnhammer EL (2002). Computational antisense oligo prediction with a neural network model.. Bioinformatics.

[33] van Deutekom JC, Bremmer-Bout M, Janson AA, Ginjaar IB, Baas F (2001). Antisense-induced exon skipping restores dystrophin expression in DMD patient derived muscle cells.. Hum Mol Genet.

[34] Staley JP, Guthrie C (1998). Mechanical devices of the spliceosome: motors, clocks, springs, and things.. Cell.

[35] Blencowe BJ (2000). Exonic splicing enhancers: mechanism of action, diversity and role in human genetic diseases.. Trends Biochem Sci.

[36] Lam BJ, Hertel KJ (2002). A general role for splicing enhancers in exon definition.. RNA.

[37] Graveley BR (2000). Sorting out the complexity of SR protein functions.. RNA.

[38] Graveley BR, Hertel KJ, Maniatis T (1999). SR proteins are ‘locators’ of the RNA splicing machinery.. Curr Biol.

[39] Wang J, Smith PJ, Krainer AR, Zhang MQ (2005). Distribution of SR protein exonic splicing enhancer motifs in human protein-coding genes.. Nucleic Acids Res.

[40] Cartegni L, Chew SL, Krainer AR (2002). Listening to silence and understanding nonsense: exonic mutations that affect splicing.. Nat Rev Genet.

[41] Blencowe BJ (2000). Exonic splicing enhancers: mechanism of action, diversity and role in human genetic diseases.. Trends Biochem Sci.

[42] Mann CJ, Honeyman K, Cheng AJ, Ly T, Lloyd F (2001). Antisense-induced exon skipping and synthesis of dystrophin in the mdx mouse.. Proc Natl Acad Sci U S A.

[43] Wilton SD, Lloyd F, Carville K, Fletcher S, Honeyman K (1999). Specific removal of the nonsense mutation from the mdx dystrophin mRNA using antisense oligonucleotides.. Neuromuscul Disord.

[44] Neugebauer KM (2002). On the importance of being co-transcriptional.. J Cell Sci.

[45] Tennyson CN, Klamut HJ, Worton RG (1995). The human dystrophin gene requires 16 hours to be transcribed and is cotranscriptionally spliced.. Nat Genet.

[46] Wuarin J, Schibler U (1994). Physical isolation of nascent RNA chains transcribed by RNA polymerase II: evidence for cotranscriptional splicing.. Mol Cell Biol.

[47] Bentley D (2002). The mRNA assembly line: transcription and processing machines in the same factory.. Curr Opin Cell Biol.

[48] Cook PR (1999). The organization of replication and transcription.. Science.

[49] Maniatis T, Reed R (2002). An extensive network of coupling among gene expression machines.. Nature.

[50] Proudfoot NJ, Furger A, Dye MJ (2002). Integrating mRNA processing with transcription.. Cell.

[51] Goldstrohm AC, Greenleaf AL, Garcia-Blanco MA (2001). Co-transcriptional splicing of pre-messenger RNAs: considerations for the mechanism of alternative splicing.. Gene.

[52] Eperon LP, Graham IR, Griffiths AD, Eperon IC (1988). Effects of RNA secondary structure on alternative splicing of pre-mRNA: is folding limited to a region behind the transcribing RNA polymerase?. Cell.

[53] Beyer AL, Osheim YN (1988). Splice site selection, rate of splicing, and alternative splicing on nascent transcripts.. Genes Dev.

[54] Osheim YN, Miller OL, Beyer AL (1985). RNP particles at splice junction sequences on Drosophila chorion transcripts.. Cell.

[55] Nowakowski J, Timoco IF, Neidle S (1999). RNA structure in solution.. Oxford Handbook of Nucleic Acid Structures.

[56] Boyle J, Robillard GT, Kim SH (1980). Sequential folding of transfer RNA. A nuclear magnetic resonance study of successively longer tRNA fragments with a common 5′ end.. J Mol Biol.

[57] Kramer FR, Mills DR (1981). Secondary structure formation during RNA synthesis.. Nucleic Acids Res.

[58] Repsilber D, Wiese S, Rachen M, Schroder AW, Riesner D (1999). Formation of metastable RNA structures by sequential folding during transcription: time-resolved structural analysis of potato spindle tuber viroid (-)-stranded RNA by temperature-gradient gel electrophoresis.. RNA.

[59] Harlepp S, Marchal T, Robert J, Leger JF, Xayaphoummine A (2003). Probing complex RNA structures by mechanical force.. Eur Phys J E Soft Matter.

[60] Meyer IM, Miklos I (2004). Co-transcriptional folding is encoded within RNA genes.. BMC Mol Biol.

[61] Harding PL, Fall AM, Honeyman K, Fletcher S, Wilton SD (2007). The influence of antisense oligonucleotide length on dystrophin exon skipping.. Mol Ther.

[62] Vickers TA, Wyatt JR, Freier SM (2000). Effects of RNA secondary structure on cellular antisense activity.. Nucleic Acids Res.

[63] Lehmann MJ, Patzel V, Sczakiel G (2000). Theoretical design of antisense genes with statistically increased efficacy.. Nucleic Acids Res.

[64] Kretschmer-Kazemi FR, Sczakiel G (2003). The activity of siRNA in mammalian cells is related to structural target accessibility: a comparison with antisense oligonucleotides.. Nucleic Acids Res.

[65] Sczakiel G (2000). Theoretical and experimental approaches to design effective antisense oligonucleotides.. Front Biosci.

[66] Cartegni L, Wang J, Zhu Z, Zhang MQ, Krainer AR (2003). ESEfinder: A web resource to identify exonic splicing enhancers.. Nucleic Acids Res.

[67] Fairbrother WG, Yeh RF, Sharp PA, Burge CB (2002). Predictive identification of exonic splicing enhancers in human genes.. Science.

[68] Errington SJ, Mann CJ, Fletcher S, Wilton SD (2003). Target selection for antisense oligonucleotide induced exon skipping in the dystrophin gene.. J Gene Med.

[69] Mathews DH, Sabina J, Zuker M, Turner DH (1999). Expanded sequence dependence of thermodynamic parameters improves prediction of RNA secondary structure.. J Mol Biol.

[70] Alberts B, Johnson A, Lewis J, Raff M, Roberts K (2002). Molecular Biology of the Cell..

[71] Beyer AL, Osheim YN (1988). Splice site selection, rate of splicing, and alternative splicing on nascent transcripts.. Genes Dev.

[72] R Development Core Team (2004). R: A language and environment for statistical computing. R Foundation for Statistical Computing, Viena, Austria. ISBN 3-900051-07-0.. http://www.R-projectorg.

