# Supplementary material for: Dynamics of Co-Transcriptional Pre-mRNA Folding Influences the Induction of Dystrophin Exon Skipping by Antisense Oligonucleotides
Source: PLoS One. 2008 Mar 26;3(3):e1844. doi: 10.1371/journal.pone.0001844 (PMC2267000; doi:10.1371/journal.pone.0001844)
Supplement: Figure S1 — Genomic lengths of all exons and introns of dystrophin gene. The sequence lengths for each of the 79 exons in dystrophin are plotted as black bars. For every exon, both of their flanking introns sequence lengths are shown as gray bars. Note that the sequence length is on a logarithmic scale (vertical axis). Total exonic and intronic sequence length are 11,034 bps and 2,209,348 respectively. The exons occupied a mere 0.5% of its full DNA sequence. The lengths of the exons range from 7 to 269 bps; introns range from 107 to 319,058 bps. (0.06 MB DOC) [file pone.0001844.s001.doc]

**Figure S1**. **Genomic lengths of all exons and introns of dystrophin gene.** The sequence lengths for each of the 79 exons in dystrophin are plotted as black bars. For every exon, both of their flanking introns sequence lengths are shown as gray bars. Note that the sequence length is on a logarithmic scale (vertical axis). Total exonic and intronic sequence length are 11,034 bps and 2,209,348 respectively. The exons occupied a mere 0.5% of its full DNA sequence. The lengths of the exons range from 7 to 269 bps; introns range from 107 to 319,058 bps.
