# Supplementary material for: Dynamics of Co-Transcriptional Pre-mRNA Folding Influences the Induction of Dystrophin Exon Skipping by Antisense Oligonucleotides
Source: PLoS One. 2008 Mar 26;3(3):e1844. doi: 10.1371/journal.pone.0001844 (PMC2267000; doi:10.1371/journal.pone.0001844)
Supplement: Figure S2 — Percentage genomic lengths of each exon relative to the total length of its flanking introns. To underscore the fact that locating an exon in dystrophin is akin to finding a needle in a haystack, the percentage of the length of an exon relative to the total length of its 3′ and 5′ intron sequences is computed and is plotted here. The majority of the exons constitute less than 1% of their intronic lengths and even the highest percentage is less than 7%. (0.06 MB DOC) [file pone.0001844.s002.doc]

**Figure S2**. **Percentage genomic lengths of each exon relative to the total length of its flanking introns.** To underscore the fact that locating an exon in dystrophin is akin to finding a needle in a haystack, the percentage of the length of an exon relative to the total length of its 3’ and 5’ intron sequences is computed and is plotted here. The majority of the exons constitute less than 1% of their intronic lengths and even the highest percentage is less than 7%.
