# Supplementary material for: Dynamics of Co-Transcriptional Pre-mRNA Folding Influences the Induction of Dystrophin Exon Skipping by Antisense Oligonucleotides
Source: PLoS One. 2008 Mar 26;3(3):e1844. doi: 10.1371/journal.pone.0001844 (PMC2267000; doi:10.1371/journal.pone.0001844)

**Figure S3.** ***Co-transcriptional* binding accessibilities of exon 57.** The horizontal axis denotes sequential steps of transcriptional analysis whereas the vertical axis denotes numbered nucleotides within the AON target site. At each step of transcriptional analysis, nucleotides in the target site that are *engaged* are depicted as a black dot in the plot.


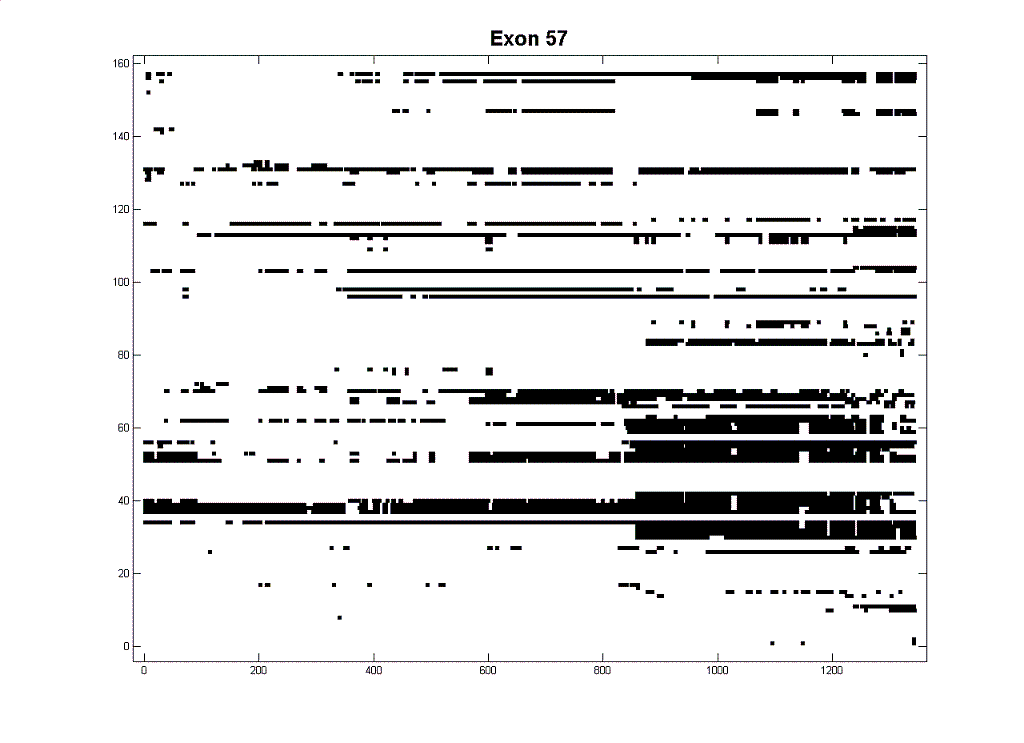

Supplement: Figure S3 — Co-transcriptional binding accessibilities of exon 57. The horizontal axis denotes sequential steps of transcriptional analysis whereas the vertical axis denotes numbered nucleotides within the AON target site. At each step of transcriptional analysis, nucleotides in the target site that are engaged are depicted as a black dot in the plot. (0.08 MB DOC) [file pone.0001844.s003.doc]
