# Supplementary material for: Dynamics of Co-Transcriptional Pre-mRNA Folding Influences the Induction of Dystrophin Exon Skipping by Antisense Oligonucleotides
Source: PLoS One. 2008 Mar 26;3(3):e1844. doi: 10.1371/journal.pone.0001844 (PMC2267000; doi:10.1371/journal.pone.0001844)
Supplement: Table S1 — First level score (L1) and third level score (L3) of 176 AON target sites analysed. This table tabulates the first level score (L1) and the third level score (L3). The AONs are sorted in ascending order of their target exon number, where the exon number is indicated in the AON names after the letter ‘h’, for e.g. h2AON1 targets exon 2. The sources of the AONs are indicated as superscripts on their names. (0.20 MB DOC) [file pone.0001844.s004.doc]

**Table S1.** **First level score (*L1*) and third level score (*L3*) of 176 AON target sites analysed.** This table tabulates the first level score (*L1*) and the third level score (*L3*). The AONs are sorted in ascending order of their target exon number, where the exon number is indicated in the AON names after the letter ‘h’, for e.g. h2AON1 targets exon 2. The sources of the AONs are indicated as superscripts on their names.

| **No.** | **AON** | **Grade** | ***L1*** | ***L3*** | **No.** | **AON** | **Grade** | ***L1*** | ***L3*** |
| --- | --- | --- | --- | --- | --- | --- | --- | --- | --- |
| **1** | H2A **^(6)^** | ++ | 0.39 | 0.084 | **89** | h47AON6 **^(10)^** | – | 0.38 | 0.109 |
| **2** | h2AON1 **^(10)^** | + + | 0.42 | 0.079 | **90** | h48AON1 **^(10)^** | – | 0.42 | 0.180 |
| **3** | h2AON2 **^(10)^** | – | 0.40 | 0.052 | **91** | h48AON2 **^(10)^** | – | 0.68 | 0.090 |
| **4** | h2AON3 **^(10)^** | – | 0.42 | 0.044 | **92** | h48AON3 **^(10)^** | – | 0.64 | 0.103 |
| **5** | H3A **^(6)^** | ++ | 0.35 | 0.075 | **93** | h48AON4 **^(10)^** | – | 0.54 | 0.245 |
| **6** | H4A **^(6)^** | ++ | 0.38 | 0.068 | **94** | h48AON6 **^(10)^** | + | 0.51 | 0.206 |
| **7** | H5A **^(6)^** | ++ | 0.44 | 0.014 | **95** | h48AON7 **^(10)^** | + | 0.62 | 0.104 |
| **8** | H6A **^(6)^** | ++ | 0.50 | 0.026 | **96** | h48AON8 **^(10)^** | – | 0.51 | 0.211 |
| **9** | H7A **^(6)^** | ++ | 0.34 | 0.189 | **97** | h48AON9 **^(10)^** | + | 0.51 | 0.164 |
| **10** | h8AON1 **^(10)^** | + + | 0.30 | 0.007 | **98** | h48AON10 **^(10)^** | + | 0.47 | 0.143 |
| **11** | h8AON3 **^(10)^** | + + | 0.54 | 0.042 | **99** | h49AON1 **^(10)^** | + + | 0.42 | 0.014 |
| **12** | H10A2 **^(6)^** | – | 0.32 | 0.283 | **100** | h49AON2 **^(10)^** | + + | 0.48 | 0.086 |
| **13** | H11A **^(6)^** | + ^1^ | 0.23 | 0.064 | **101** | H50A **^(6)^** | + ^2^ | 0.36 | 0.178 |
| **14** | H12A **^(6)^** | ++ | 0.57 | 0.039 | **102** | h50AON1 **^(10)^** | + + | 0.42 | 0.129 |
| **15** | H13A **^(6)^** | ++ | 0.37 | 0.131 | **103** | h50AON2 **^(10)^** | + | 0.40 | 0.208 |
| **16** | H14A **^(6)^** | ++ | 0.31 | 0.183 | **104** | H51A **^(6)^** | ++ | 0.58 | 0.048 |
| **17** | H15A **^(6)^** | ++ | 0.29 | 0.025 | **105** | h51AON1 **^(10)^** | + + | 0.62 | 0.032 |
| **18** | h17AON1 **^(10)^** | + + | 0.43 | 0.169 | **106** | h51AON24 **^(10)^** | – | 0.44 | 0.057 |
| **19** | h17AON2 **^(10)^** | + | 0.61 | 0.046 | **107** | h51AON27 **^(10)^** | – | 0.36 | 0.251 |
| **20** | H18A **^(6)^** | ++ | 0.42 | 0.025 | **108** | h51AON2 **^(10)^** | + + | 0.40 | 0.008 |
| **21** | H19A **^(6)^** | + + | 0.42 | 0.044 | **109** | h51AON29 **^(10)^** | + + | 0.26 | 0.380 |
| **22** | h19AON **^(10)^** | + | 0.38 | 0.002 | **110** | H52A **^(6)^** | ++ | 0.35 | 0.044 |
| **23** | H20A1 **^(6)^** | – | 0.29 | 0.226 | **111** | h52AON1 **^(10)^** | + | 0.48 | 0.029 |
| **24** | H20A2 **^(6)^** | – | 0.25 | 0.255 | **112** | h52AON2 **^(10)^** | – | 0.54 | 0.001 |
| **25** | H21A **^(6)^** | + ^1^ | 0.48 | 0.027 | **113** | H53A **^(6)^** | ++ | 0.43 | 0.101 |
| **26** | H22A **^(6)^** | ++ | 0.29 | 0.068 | **114** | h53AON1 **^(10)^** | + | 0.53 | 0.092 |
| **27** | H23A **^(6)^** | ++ | 0.44 | 0.035 | **115** | h53AON2 **^(10)^** | – | 0.42 | 0.191 |
| **28** | H24A **^(6)^** | ++ | 0.34 | 0.078 | **116** | H54A **^(6)^** | – | 0.28 | 0.373 |
| **29** | H25A **^(6)^** | ++ | 0.39 | 0.191 | **117** | h54AON1 **^(10)^** | + + | 0.32 | 0.140 |
| **30** | H27A **^(6)^** | ++ | 0.40 | 0.085 | **118** | h54AON2 **^(10)^** | + + | 0.32 | 0.163 |
| **31** | H28A **^(6)^** | ++ | 0.28 | 0.141 | **119** | H55A **^(6)^** | + ^2^ | 0.39 | 0.236 |
| **32** | H29A **^(6)^** | ++ | 0.48 | 0.011 | **120** | h55AON1 **^(10)^** | + | 0.48 | 0.055 |
| **33** | h29AON1 **^(10)^** | + + | 0.35 | 0.028 | **121** | h55AON2 **^(10)^** | + | 0.60 | 0.080 |
| **34** | h29AON2 **^(10)^** | + + | 0.42 | 0.021 | **122** | h55AON3 **^(10)^** | + | 0.50 | 0.020 |
| **35** | h29AON4 **^(10)^** | + + | 0.56 | 0.007 | **123** | h55AON5 **^(10)^** | + + | 0.40 | 0.287 |
| **36** | h29AON6 **^(10)^** | + + | 0.43 | 0.061 | **124** | h55AON6 **^(10)^** | + + | 0.39 | 0.226 |
| **37** | h29AON9 **^(10)^** | + | 0.37 | 0.162 | **125** | h56AON1 **^(10)^** | + | 0.57 | 0.044 |
| **38** | h29AON10 **^(10)^** | – | 0.47 | 0.083 | **126** | h56AON2 **^(10)^** | – | 0.46 | 0.187 |
| **39** | h29AON11 **^(10)^** | + | 0.46 | 0.052 | **127** | h56AON3 **^(10)^** | + | 0.44 | 0.008 |
| **40** | H30A **^(6)^** | ++ | 0.52 | 0.295 | **128** | H56A **^(6)^** | + ^2^ | 0.40 | 0.082 |
| **41** | H32A **^(6)^** | ++ | 0.35 | 0.085 | **129** | h57AON1 **^(10)^** | – | 0.39 | 0.109 |
| **42** | H33A **^(6)^** | ++ | 0.31 | 0.024 | **130** | h57AON2 **^(10)^** | – | 0.40 | 0.086 |
| **43** | H34A1 **^(6)^** | – | 0.45 | 0.030 | **131** | h57AON3 **^(10)^** | – | 0.30 | 0.106 |
| **44** | H34A2 **^(6)^** | – | 0.47 | 0.011 | **132** | H58A **^(6)^** | + ^2^ | 0.61 | 0.0126 |
| **45** | H35A **^(6)^** | ++ | 0.45 | 0.075 | **133** | h58AON1 **^(10)^** | – | 0.57 | 0.058 |
| **46** | H36A **^(6)^** | + ^1^ | 0.43 | 0.049 | **134** | h58AON2 **^(10)^** | + | 0.38 | 0.136 |
| **47** | H37A **^(6)^** | ++ | 0.36 | 0.060 | **135** | h59AON1 **^(10)^** | – | 0.51 | 0.050 |
| **48** | H38A **^(6)^** | ++ | 0.38 | 0.016 | **136** | h59AON2 **^(10)^** | + + | 0.49 | 0.054 |
| **49** | H39A **^(6)^** | ++ | 0.39 | 0.004 | **137** | H60A **^(6)^** | + ^1^ | 0.35 | 0.050 |
| **50** | h40AON1 **^(10)^** | + + | 0.55 | 3.12E-4 | **138** | h60AON1 **^(10)^** | + | 0.34 | 0.074 |
| **51** | h40AON2 **^(10)^** | + + | 0.35 | 0.016 | **139** | h60AON2 **^(10)^** | – | 0.70 | 0.002 |
| **52** | H41A **^(6)^** | ++ | 0.26 | 0.195 | **140** | H61A **^(6)^** | + ^1^ | 0.39 | 0.098 |
| **53** | h41AON1 **^(10)^** | + + | 0.41 | 0.031 | **141** | h61AON1 **^(10)^** | – | 0.51 | 0.067 |
| **54** | h41AON2 **^(10)^** | + | 0.48 | 0.096 | **142** | h61AON2 **^(10)^** | + | 0.39 | 0.034 |
| **55** | h42AON1 **^(10)^** | + | 0.47 | 0.034 | **143** | H62A **^(6)^** | + ^2^ | 0.31 | 0.167 |
| **56** | h42AON2 **^(10)^** | + | 0.29 | 0.141 | **144** | h62AON1 **^(10)^** | + + | 0.35 | 0.201 |
| **57** | H43A **^(6)^** | + ^1^ | 0.36 | 0.140 | **145** | h62AON2 **^(10)^** | – | 0.46 | 0.022 |
| **58** | h43AON1 **^(10)^** | – | 0.44 | 0.125 | **146** | H63A **^(6)^** | + ^2^ | 0.53 | 0.073 |
| **59** | h43AON2 **^(10)^** | + | 0.53 | 0.056 | **147** | h63AON1 **^(10)^** | + | 0.59 | 0.050 |
| **60** | h43AON3 **^(10)^** | – | 0.50 | 0.054 | **148** | h63AON2 **^(10)^** | + | 0.41 | 0.066 |
| **61** | h43AON4 **^(10)^** | – | 0.43 | 0.099 | **149** | H64A **^(6)^** | + ^2^ | 0.40 | 0.015 |
| **62** | h43AON5 **^(10)^** | + + | 0.44 | 0.175 | **150** | H65A **^(6)^** | – | 0.28 | 0.071 |
| **63** | H44A **^(6)^** | + ^1^ | 0.34 | 0.175 | **151** | H67A **^(6)^** | + ^2^ | 0.36 | 0.077 |
| **64** | h44AON1 **^(10)^** | + + | 0.30 | 0.077 | **152** | H68A **^(6)^** | + ^1^ | 0.35 | 0.214 |
| **65** | h44AON2 **^(10)^** | + + | 0.47 | 0.002 | **153** | H70A **^(6)^** | + ^1^ | 0.39 | 0.148 |
| **66** | h45AON1 **^(10)^** | – | 0.46 | 0.025 | **154** | h71AON1 **^(10)^** | + + | 0.41 | 0.004 |
| **67** | h45AON2 **^(10)^** | – | 0.54 | 0.031 | **155** | h71AON2 **^(10)^** | + + | 0.34 | 0.056 |
| **68** | h45AON3 **^(10)^** | – | 0.54 | 0.022 | **156** | H72A **^(6)^** | ++ | 0.51 | 0.019 |
| **69** | h45AON4 **^(10)^** | – | 0.35 | 0.026 | **157** | h72AON1 **^(10)^** | + + | 0.50 | 0.009 |
| **70** | h45AON5 **^(10)^** | + | 0.27 | 0.264 | **158** | h72AON2 **^(10)^** | + | 0.43 | 0.173 |
| **71** | h45AON9 **^(10)^** | – | 0.30 | 0.097 | **159** | H73A **^(6)^** | + ^1^ | 0.49 | 0.022 |
| **72** | H46A **^(6)^** | + ^1^ | 0.42 | 0.042 | **160** | h73AON1 **^(10)^** | + + | 0.47 | 0.017 |
| **73** | h46AON4 **^(10)^** | + | 0.11 | 0.271 | **161** | h73AON2 **^(10)^** | + | 0.29 | 0.221 |
| **74** | h46AON6 **^(10)^** | + | 0.24 | 0.203 | **162** | H74A **^(6)^** | ++ | 0.45 | 0.106 |
| **75** | h46AON8 **^(10)^** | + + | 0.42 | 0.037 | **163** | h74AON1 **^(10)^** | + + | 0.54 | 0.028 |
| **76** | h46AON9 **^(10)^** | – | 0.43 | 0.049 | **164** | h74AON2 **^(10)^** | + | 0.22 | 0.135 |
| **77** | h46AON20 **^(10)^** | + | 0.45 | 0.093 | **165** | H75A **^(6)^** | ++ | 0.42 | 0.046 |
| **78** | h46AON21 **^(10)^** | – | 0.46 | 0.115 | **166** | h75AON1 **^(10)^** | + + | 0.46 | 0.061 |
| **79** | h46AON22 **^(10)^** | + + | 0.45 | 0.013 | **167** | h75AON2 **^(10)^** | + + | 0.48 | 0.025 |
| **80** | h46AON23 **^(10)^** | + + | 0.37 | 0.017 | **168** | H76A **^(6)^** | + ^2^ | 0.30 | 0.101 |
| **81** | h46AON24 **^(10)^** | + | 0.43 | 0.033 | **169** | h76AON1 **^(10)^** | – | 0.19 | 0.280 |
| **82** | h46AON25 **^(10)^** | + | 0.58 | 0.059 | **170** | h76AON2 **^(10)^** | + | 0.38 | 0.067 |
| **83** | h46AON26 **^(10)^** | + + | 0.49 | 8.90E-6 | **171** | H77A **^(6)^** | ++ | 0.47 | 0.129 |
| **84** | h47AON1 **^(10)^** | – | 0.39 | 0.026 | **172** | h77AON1 **^(10)^** | + + | 0.49 | 0.151 |
| **85** | h47AON2 **^(10)^** | – | 0.47 | 0.132 | **173** | h77AON2 **^(10)^** | + + | 0.26 | 0.312 |
| **86** | h47AON3 **^(10)^** | – | 0.60 | 0.014 | **174** | H78A **^(6)^** | ++ | 0.46 | 0.064 |
| **87** | h47AON4 **^(10)^** | – | 0.49 | 0.121 | **175** | h78AON1 **^(10)^** | + + | 0.53 | 0.018 |
| **88** | h47AON5 **^(10)^** | – | 0.55 | 0.008 | **176** | h78AON2 **^(10)^** | + + | 0.44 | 0.024 |
