# Supplementary material for: Dynamics of Co-Transcriptional Pre-mRNA Folding Influences the Induction of Dystrophin Exon Skipping by Antisense Oligonucleotides
Source: PLoS One. 2008 Mar 26;3(3):e1844. doi: 10.1371/journal.pone.0001844 (PMC2267000; doi:10.1371/journal.pone.0001844)
Supplement: Table S2 — The nucleotide accessibility score of all nucleotide in an AON target site is plotted for all the 176 AONs analysed. The horizontal axis represents the nucleotide position in the respective target exon and the nucleotide accessibility score is plotted on the vertical axis. (0.48 MB DOC) [file pone.0001844.s005.doc]

**Table S2.** **The nucleotide accessibility score of all nucleotide in an AON target site is plotted for all the 176 AONs analysed.** The horizontal axis represents the nucleotide position in the respective target exon and the nucleotide accessibility score is plotted on the vertical axis.

| h2AON1 (++) | h2AON2 (–) | h2AON3 (–) |
| --- | --- | --- |
| h8AON1 (++) | h8AON3 (++) | h17AON1 (++) |
| h17AON2 (+) | h19AON (+) | h29AON1 (++) |
| h29AON2 (++) | h29AON4 (++) | h29AON6 (++) |
| h29AON9 (+) | h29AON10 (–) | h29AON11 (+) |
| h40AON1 (++) | h40AON2 (++) | h41AON1 (++) |
| h41AON2 (+) | h42AON1 (+) | h42AON2 (+) |
| h43AON1 (–) | h43AON2 (+) | h43AON3 (–) |
| h43AON4 (–) | h43AON5 (++) | h44AON1 (++) |
| h44AON2 (++) | h45AON1 (–) | h45AON2 (–) |
| h45AON3 (–) | h45AON4 (–) | h45AON5 (+) |
| h45AON9 (–) | h46AON4 (+) | h46AON6 (+) |
| h46AON8 (++) | h46AON9 (–) | h46AON20 (+) |
| h46AON21 (–) | h46AON22 (++) | h46AON23 (++) |
| h46AON24 (+) | h46AON25 (+) | h46AON26 (++) |
| h47AON1 (–) | h47AON2 (–) | h47AON3 (–) |
| h47AON4 (–) | h47AON5 (–) | h47AON6 (–) |
| h48AON1 (–) | h48AON2 (–) | h48AON3 (–) |
| h48AON4 (–) | h48AON6 (+) | h48AON7 (+) |
| h48AON8 (–) | h48AON9 (+) | h48AON10 (+) |
| h49AON1 (++) | h49AON2 (++) | h50AON1 (++) |
| h50AON2 (+) | h51AON1 (++) | h51AON24 (–) |
| h51AON27 (–) | h51AON2 (++) | h51AON29 (++) |
| h52AON1 (+) | h52AON2 (–) | h53AON1 (+) |
| h53AON2 (–) | h54AON1 (++) | h54AON2 (++) |
| h55AON1 (+) | h55AON2 (+) | h55AON3 (+) |
| h55AON5 (++) | h55AON6 (++) | h56AON1 (+) |
| h56AON2 (–) | h56AON3 (+) | h57AON1 (–) |
| h57AON2 (–) | h57AON3 (–) | h58AON1 (–) |
| h58AON2 (+) | h59AON1 (–) | h59AON2 (++) |
| h60AON1 (+) | h60AON2 (–) | h61AON1 (–) |
| h61AON2 (+) | h62AON1 (++) | h62AON2 (–) |
| h63AON1 (+) | h63AON2 (+) | h71AON1 (++) |
| h71AON2 (++) | h72AON1 (++) | h72AON2 (+) |
| h73AON1 (++) | h73AON2 (+) | h74AON1 (++) |
| h74AON2 (+) | h75AON1 (++) | h75AON2 (++) |
| h76AON1 (–) | h76AON2 (+) | h77AON1 (++) |
| h77AON2 (++) | h78AON1 (++) | h78AON2 (++) |
|  | | |
| H2A (++) | H3A (++) | H4A (++) |
| H5A (++) | H6A (++) | H7A (++) |
| H10A2 (–) | H11A (+ ^1^) | H12A (++) |
| H13A (++) | H14A (++) | H15A (++) |
| H18A (++) | H19A (++) | H20A1 (–) |
| H20A2 (–) | H21A (+ ^1^) | H22A (++) |
| H23A (++) | H24A (++) | H25A (++) |
| H27A (++) | H28A (++) | H29A (++) |
| H30A (++) | H32A (++) | H33A (++) |
| H34A1 (–) | H34A2 (–) | H35A (++) |
| H36A (+ ^1^) | H37A (++) | H38A (++) |
| H39A (++) | H41A (++) | H43A (+ ^1^) |
| H44A (+ ^1^) | H46A (+ ^1^) | H50A (+ ^2^) |
| H51A (++) | H52A (++) | H53A (++) |
| H54A (–) | H55A (+ ^2^) | H56A (+ ^2^) |
| H58A (+ ^2^) | H60A (+ ^1^) | H61A (+ ^1^) |
| H62A (+ ^2^) | H63A (+ ^2^) | H64A (+ ^2^) |
| H65A2 (–) | H67A (+ ^2^) | H68A (+ ^1^) |
| H70A (+ ^1^) | H72A (++) | H73A (+ ^1^) |
| H74A (++) | H75A (++) | H76A (+ ^2^) |
| H77A (++) | H78A (++) |  |
