# Supplementary material for: Dynamics of Co-Transcriptional Pre-mRNA Folding Influences the Induction of Dystrophin Exon Skipping by Antisense Oligonucleotides
Source: PLoS One. 2008 Mar 26;3(3):e1844. doi: 10.1371/journal.pone.0001844 (PMC2267000; doi:10.1371/journal.pone.0001844)
Supplement: Table S3 — Occurrences of engaged nucleotides at each step of transcriptional analysis for all nucleotide in an AON target site are depicted for each of the 176 AON target sites analysed. The horizontal axis denotes sequential steps of transcriptional analysis while the vertical axis denotes numbered nucleotides within the AON target site. At each step of transcriptional analysis, nucleotides in the target site that are engaged are depicted as a black dot in the plot. (1.93 MB DOC) [file pone.0001844.s006.doc]

**Table S3.** **Occurrences of *engaged* nucleotides at each step of transcriptional analysis for all nucleotide in an AON target site are depicted for each of the 176 AON target sites analysed.** The horizontal axis denotes sequential steps of transcriptional analysis while the vertical axis denotes numbered nucleotides within the AON target site. At each step of transcriptional analysis, nucleotides in the target site that are *engaged* are depicted as a black dot in the plot.

| h2AON1 (++)  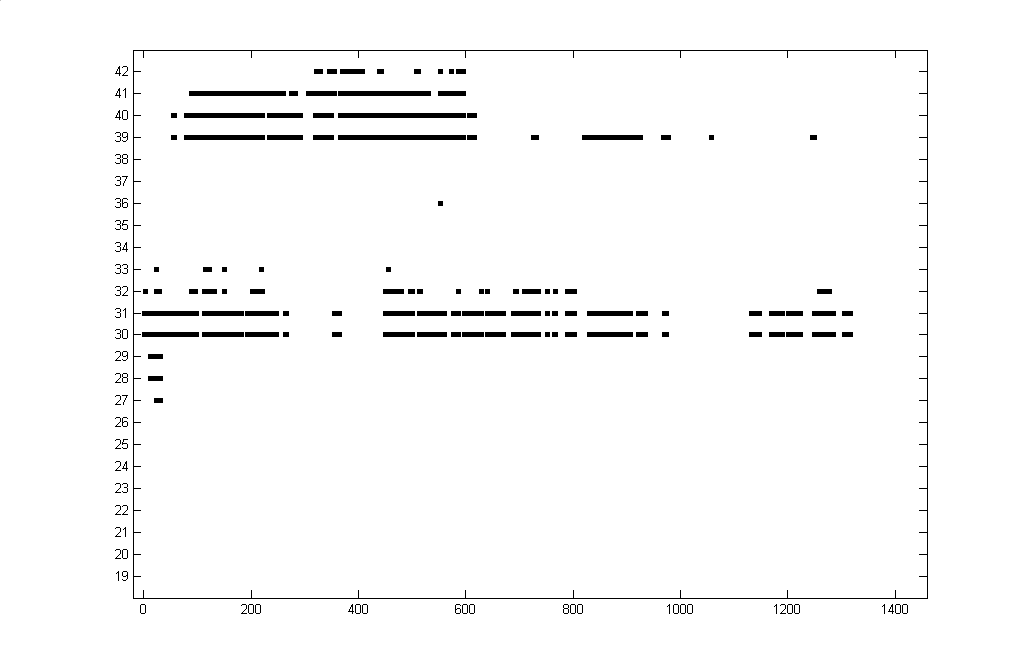 | h8AON1 (++)  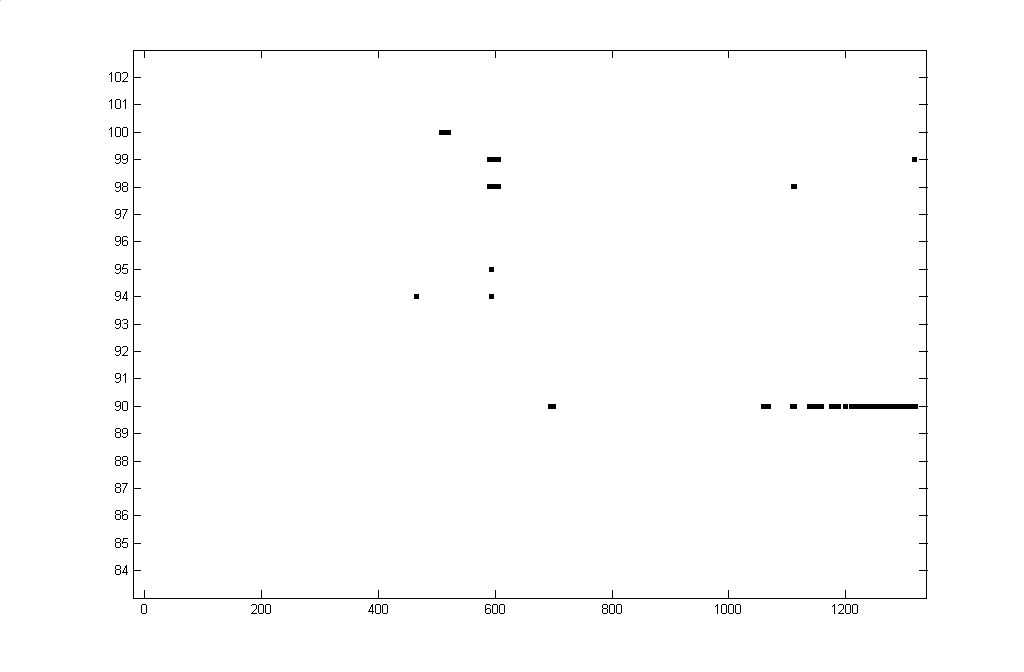 | h8AON3 (++)  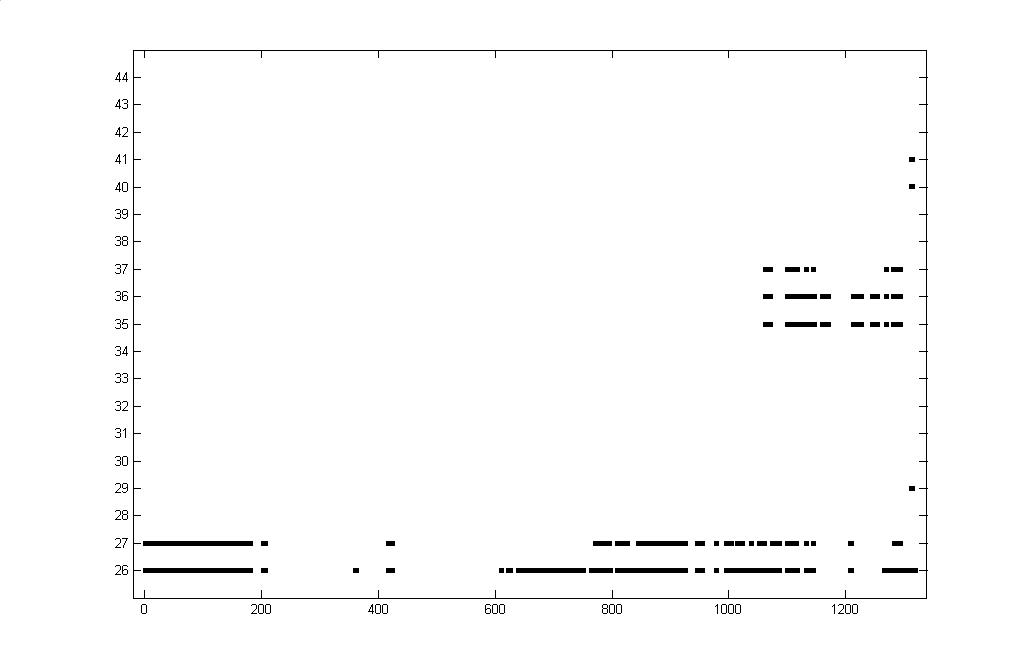 |
| --- | --- | --- |
| h17AON1 (++)  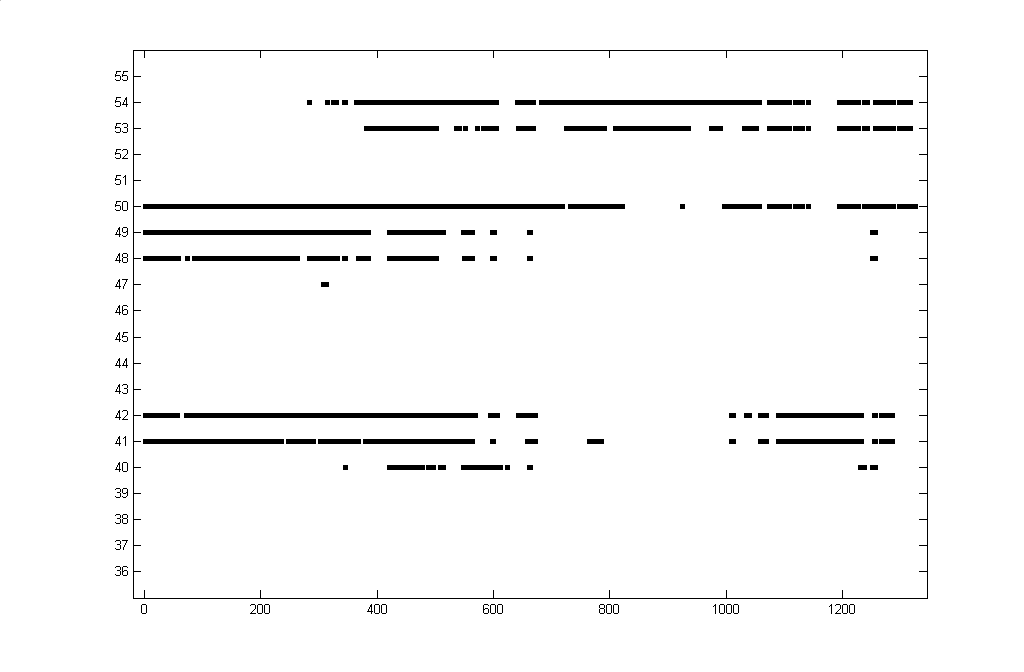 | h29AON1 (++)  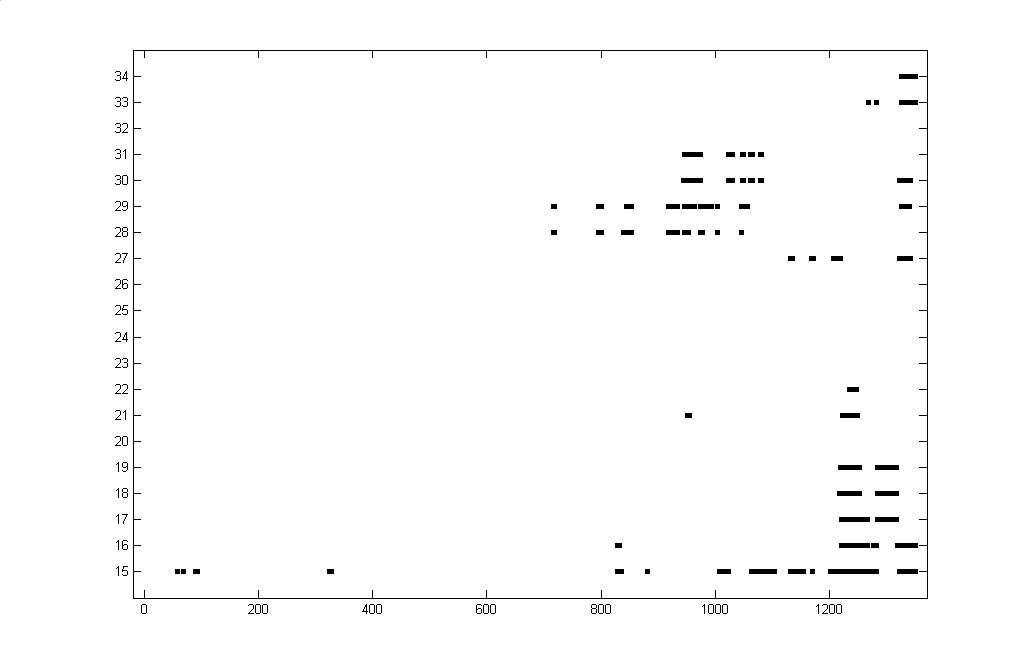 | h29AON2 (++)  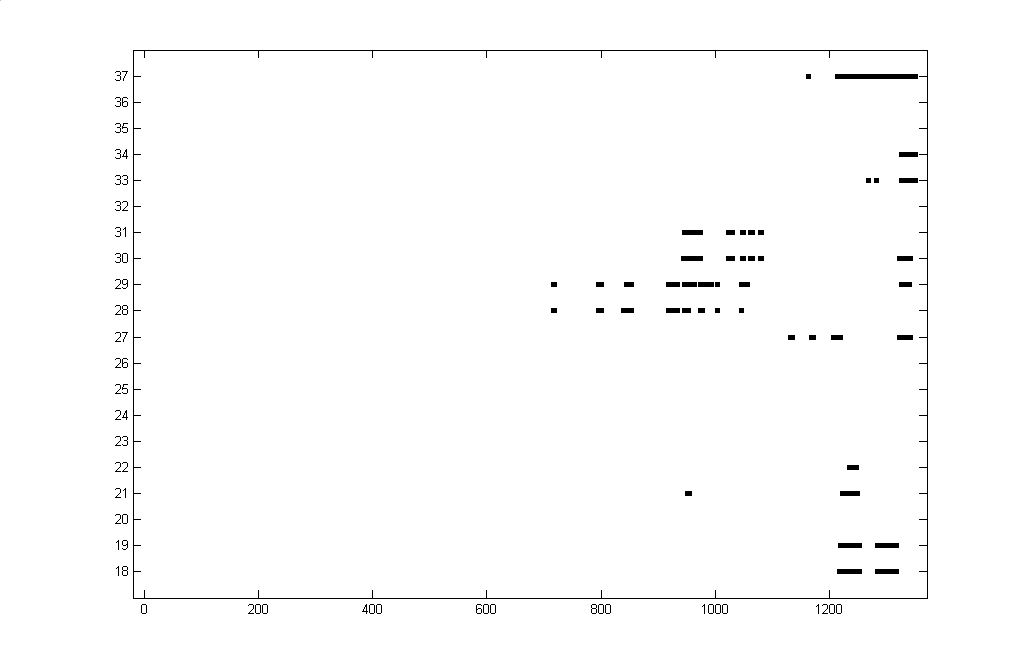 |
| h29AON4 (++)  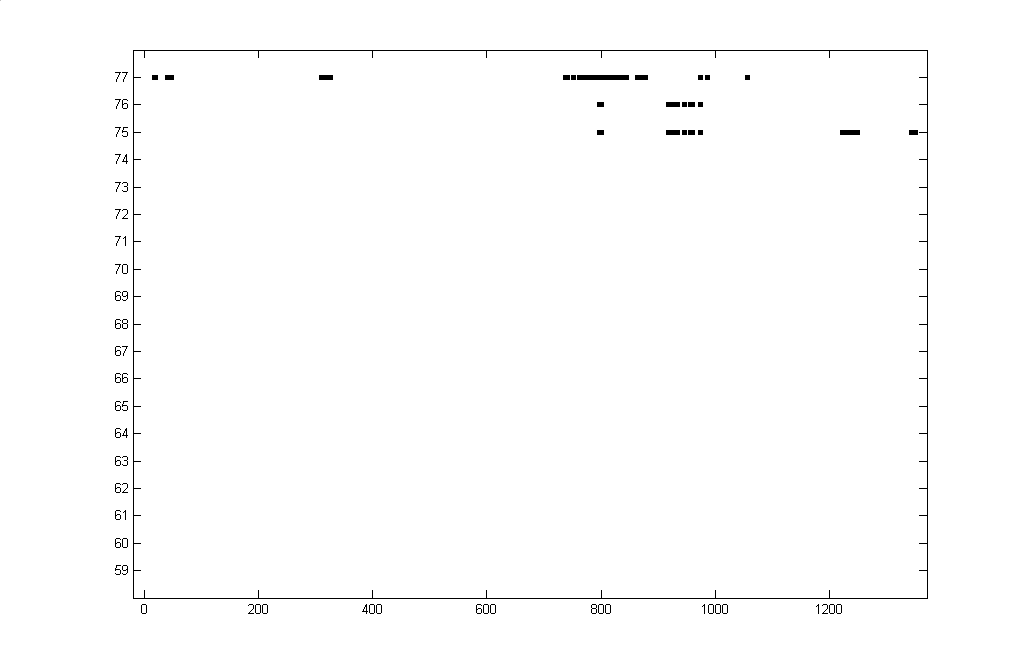 | h29AON6 (++)  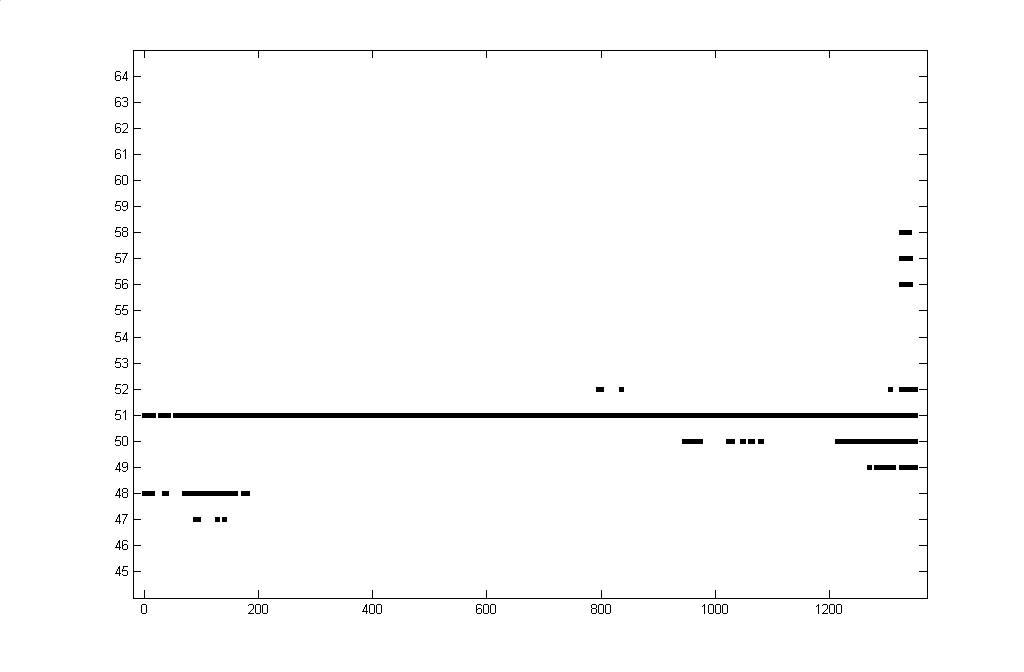 | h40AON1 (++)  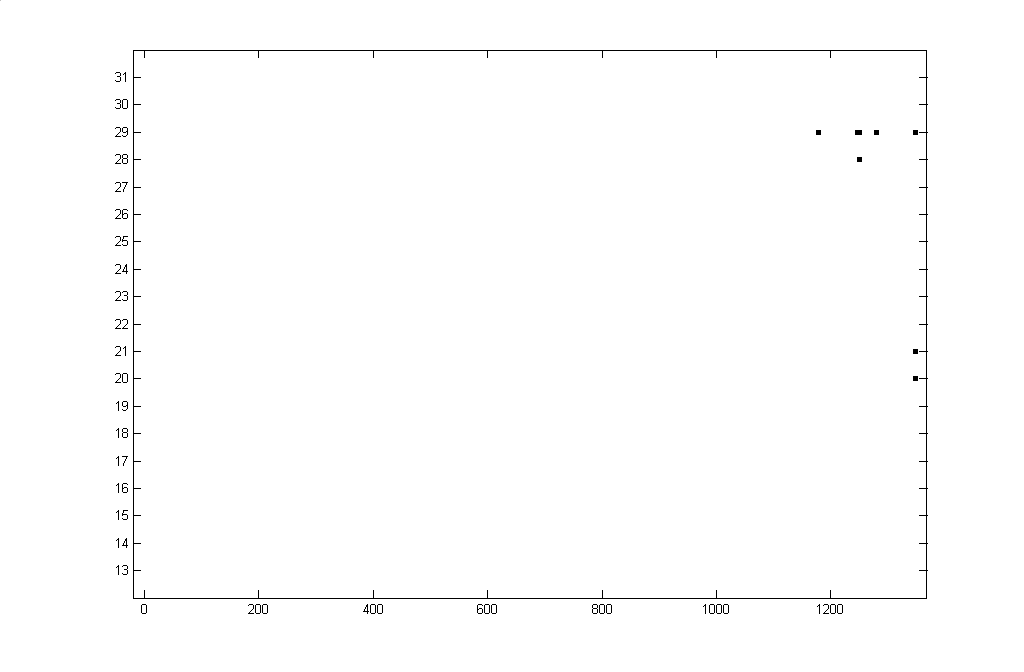 |
| h40AON2 (++)  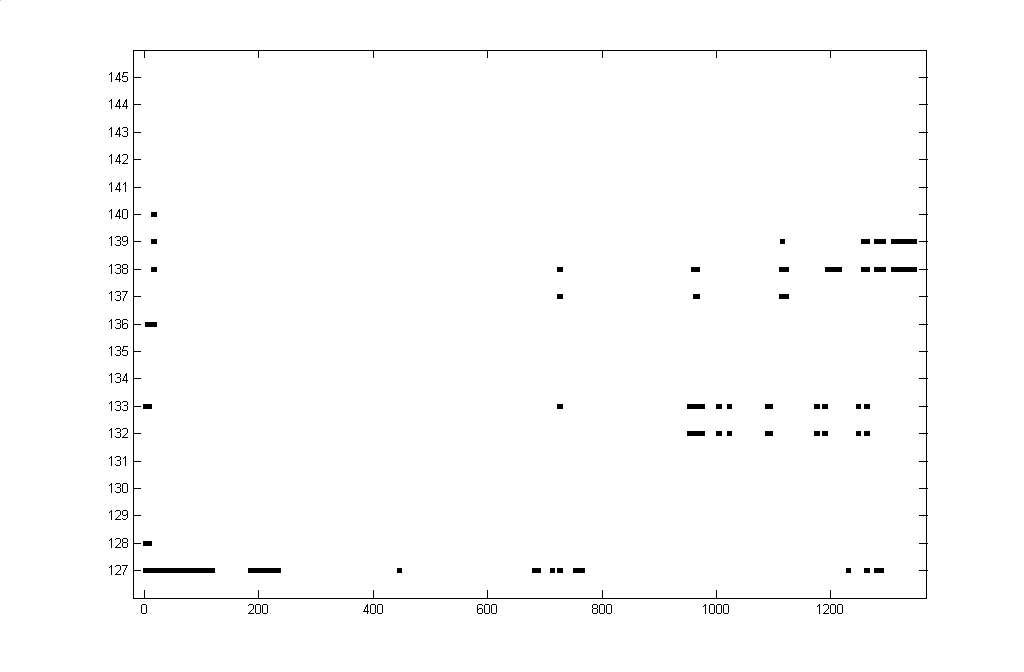 | h41AON1 (++)  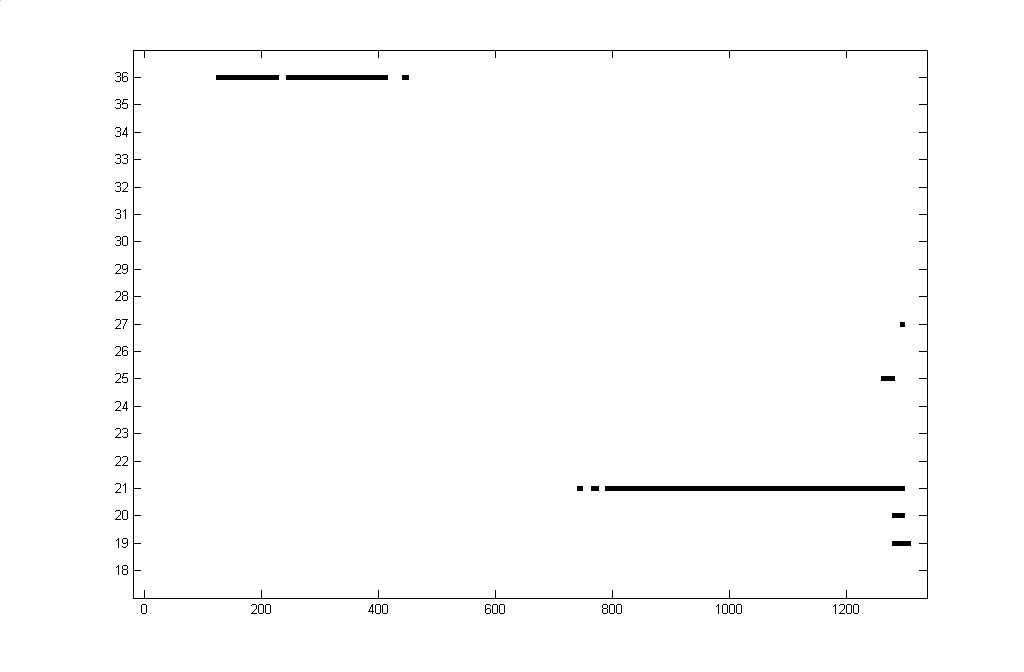 | h43AON5 (++)  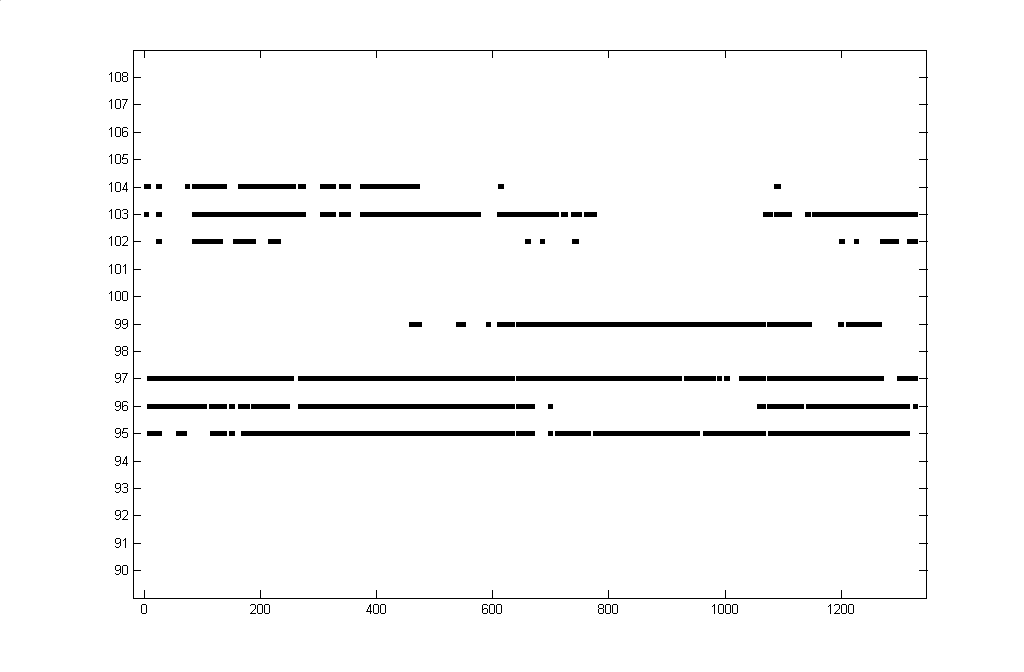 |
| h44AON1 (++)  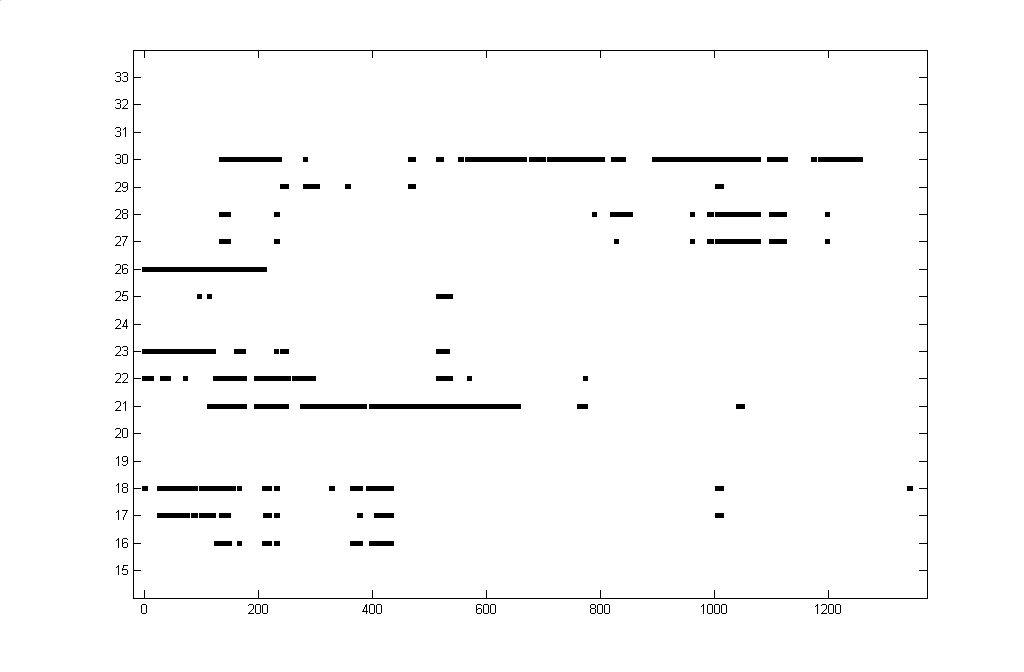 | h44AON2 (++)  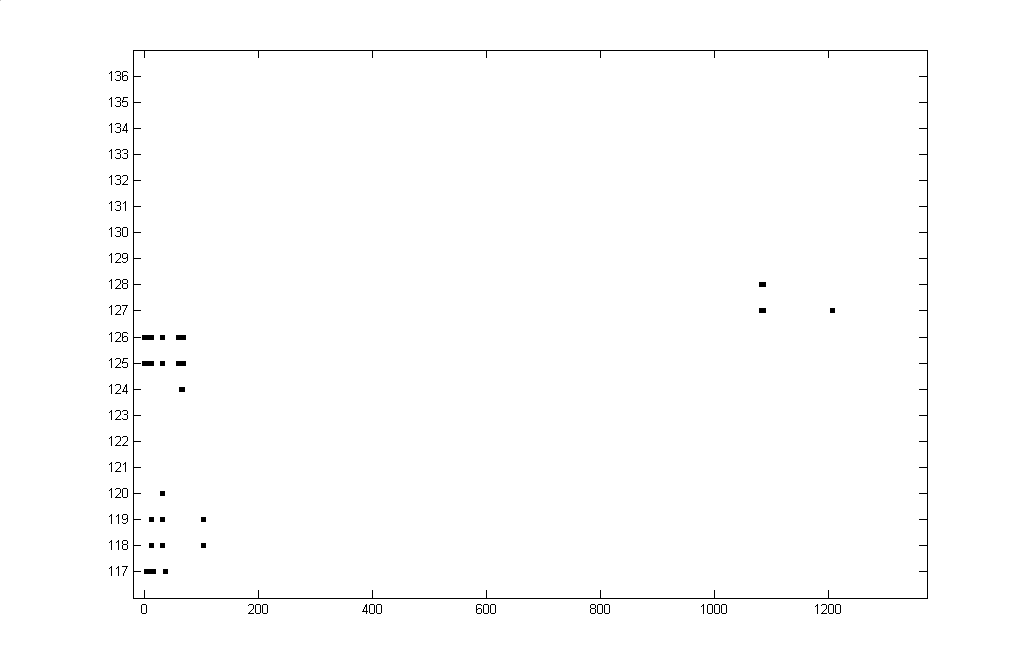 | h46AON8 (++)  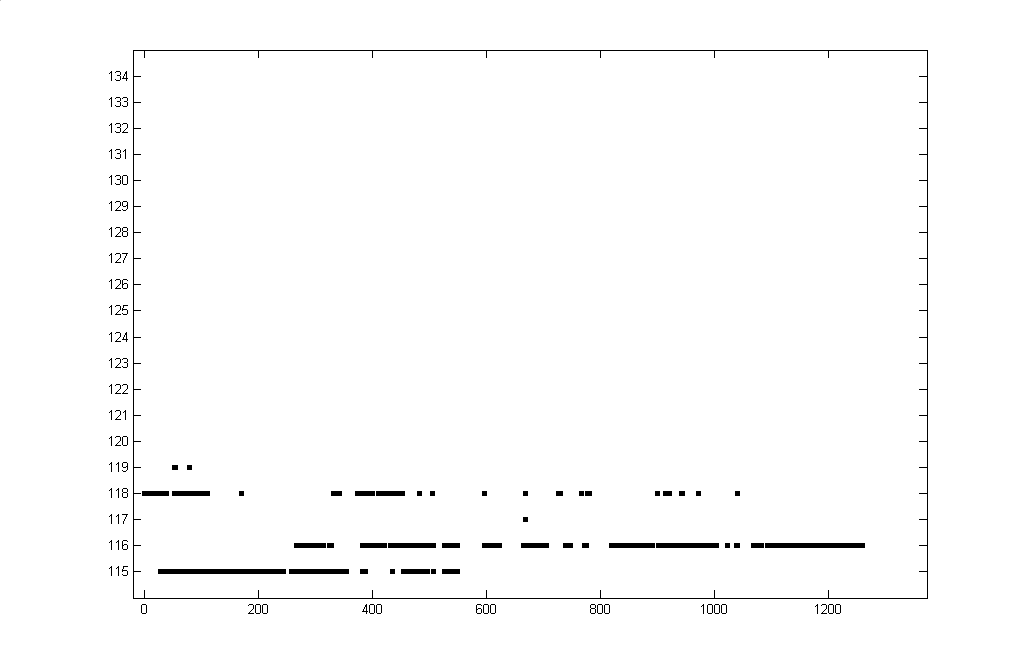 |
| h46AON22 (++)  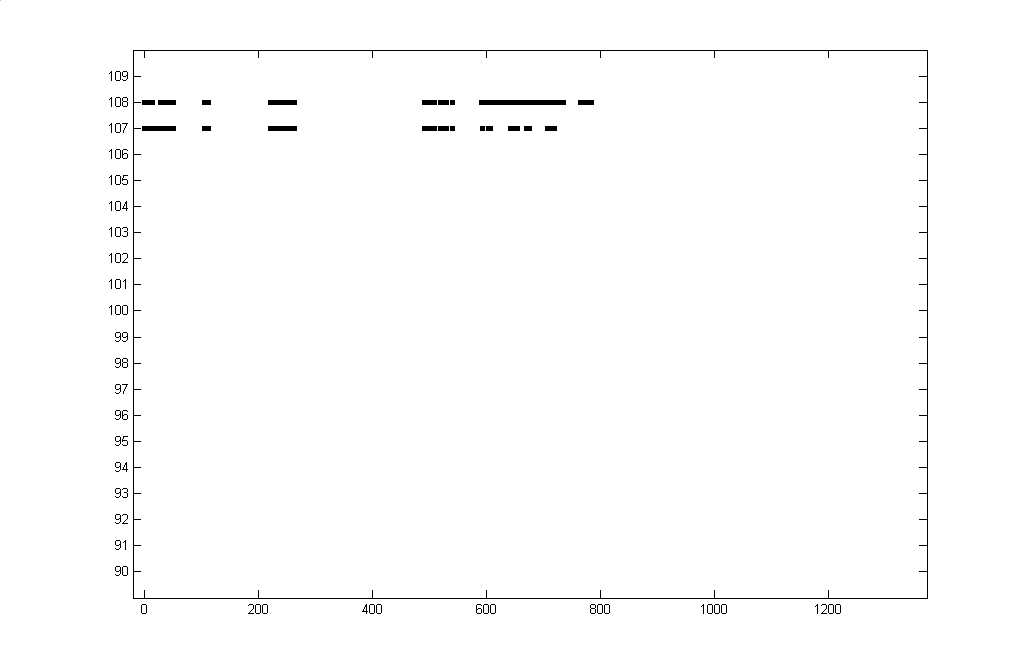 | h46AON23 (++)  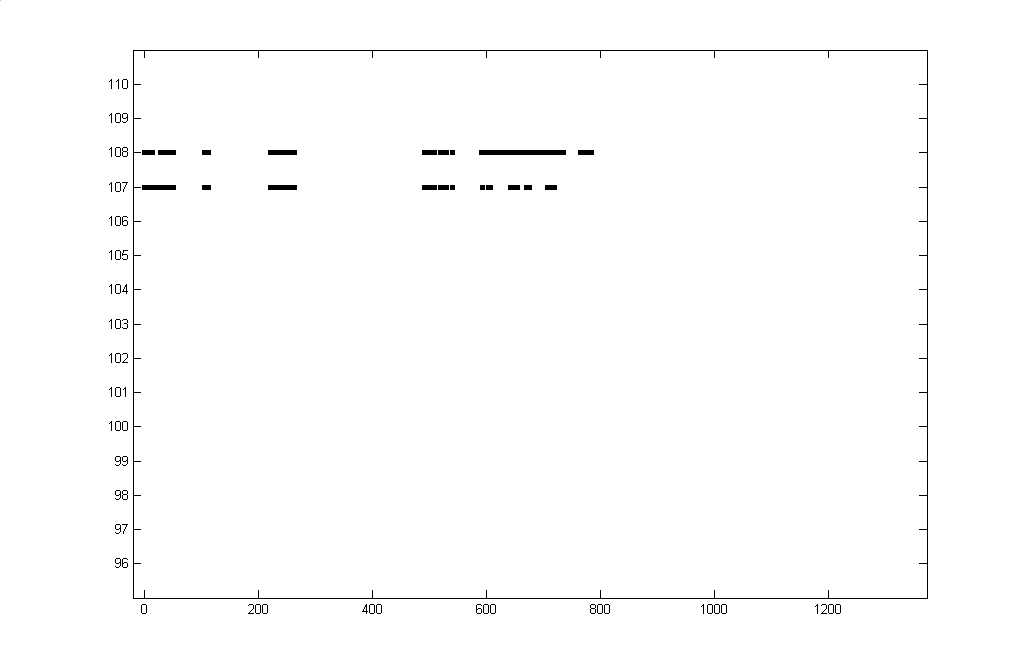 | h46AON26 (++)  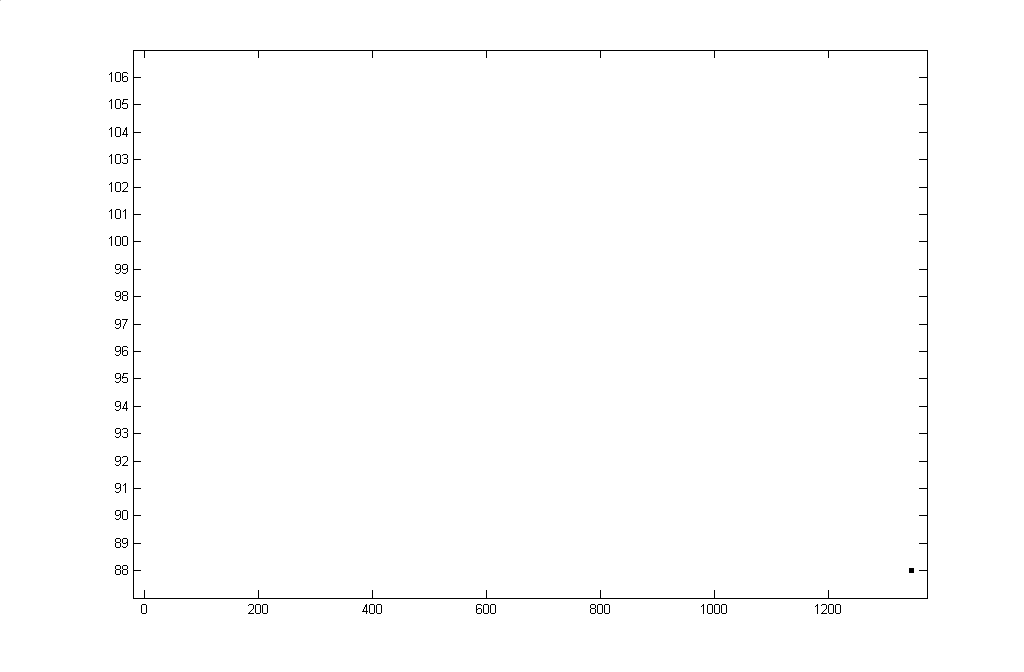 |
| h49AON1 (++)  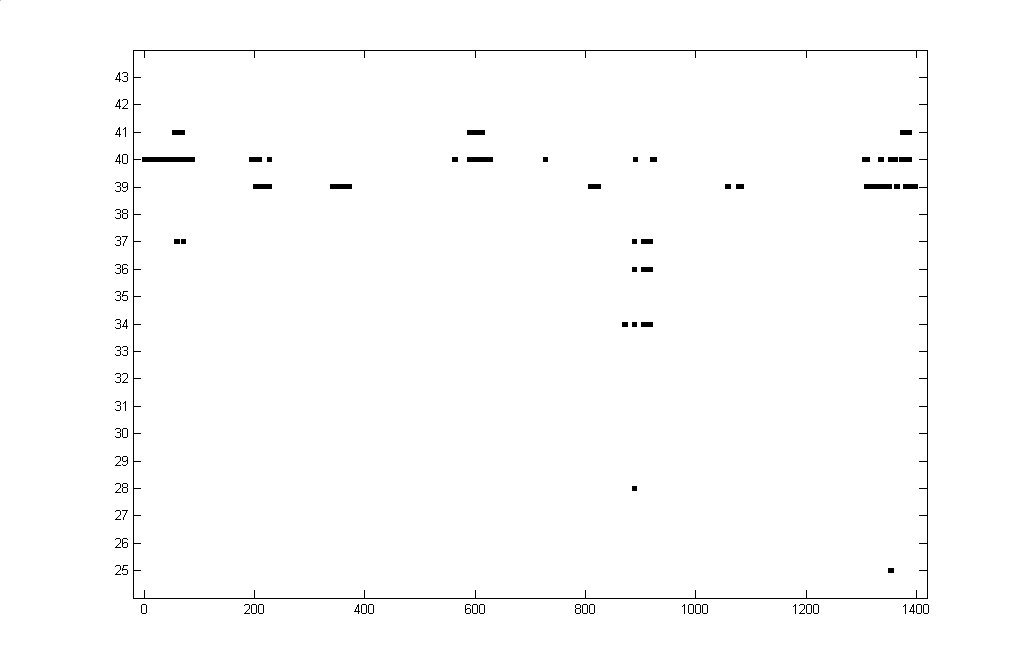 | h49AON2 (++)  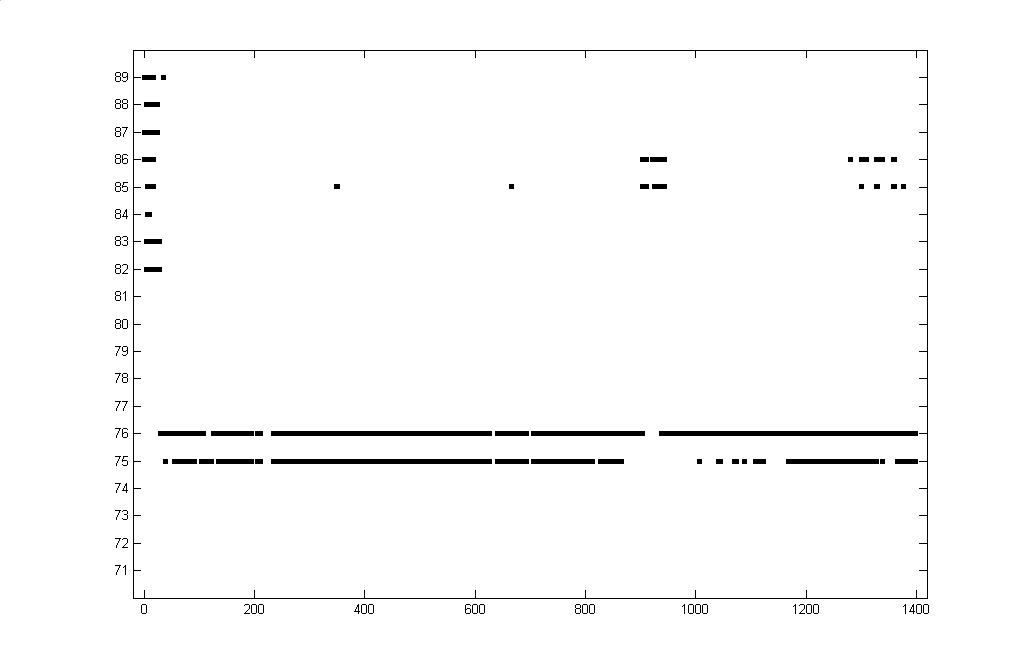 | h50AON1 (++)  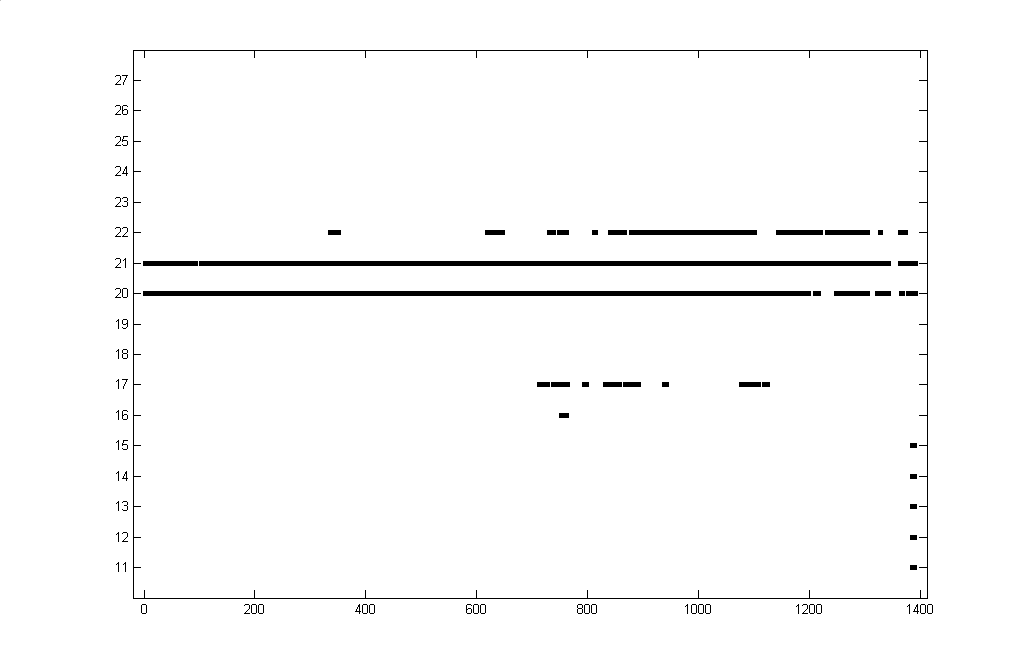 |
| h51AON1 (++)  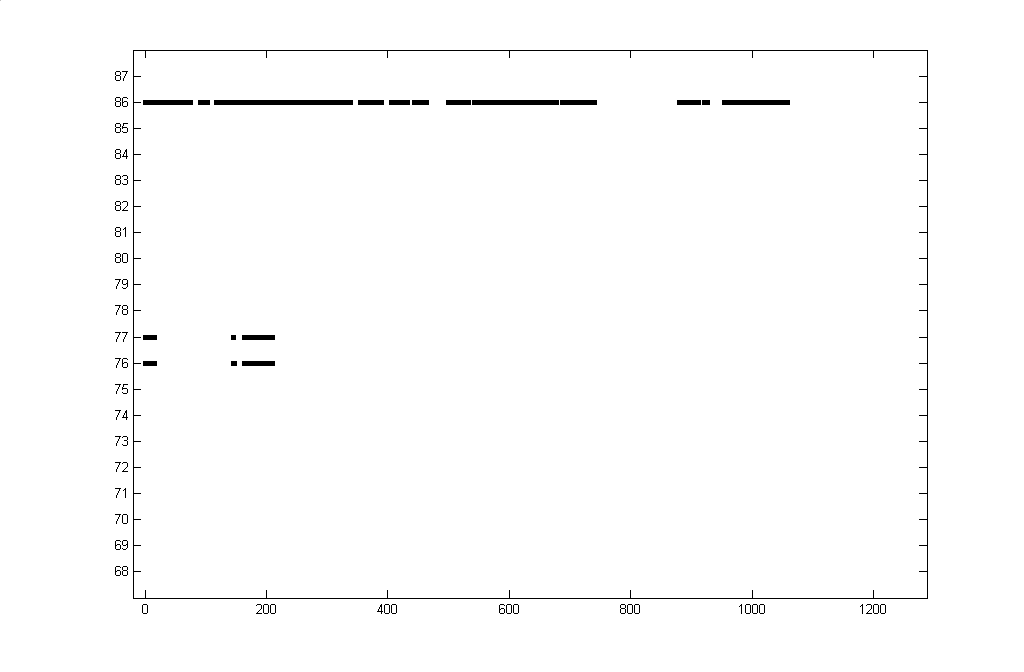 | h51AON2 (++)  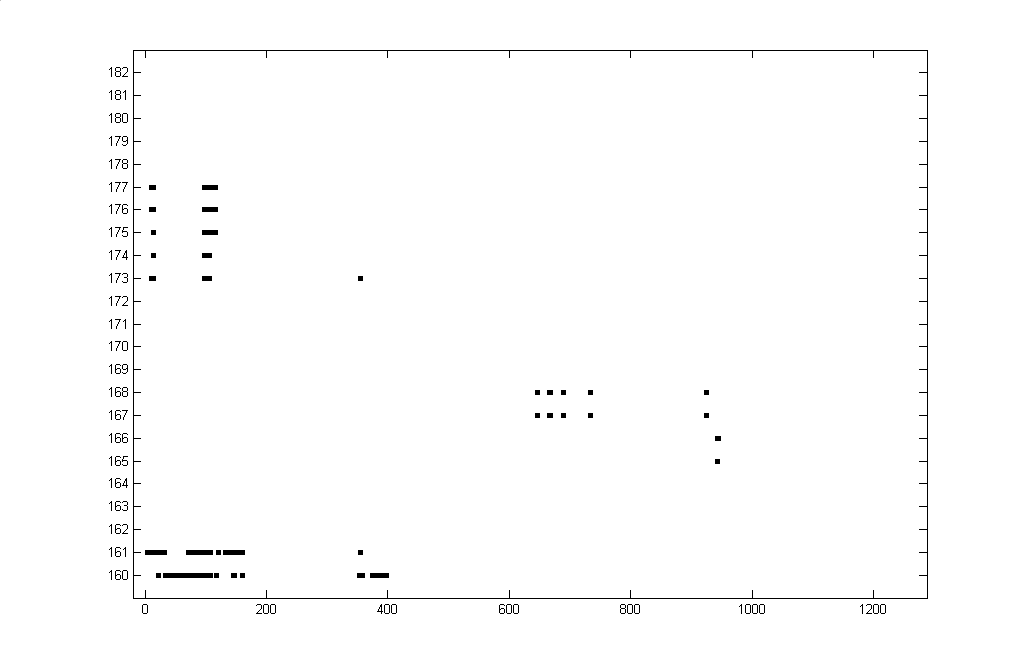 | h51AON29 (++)  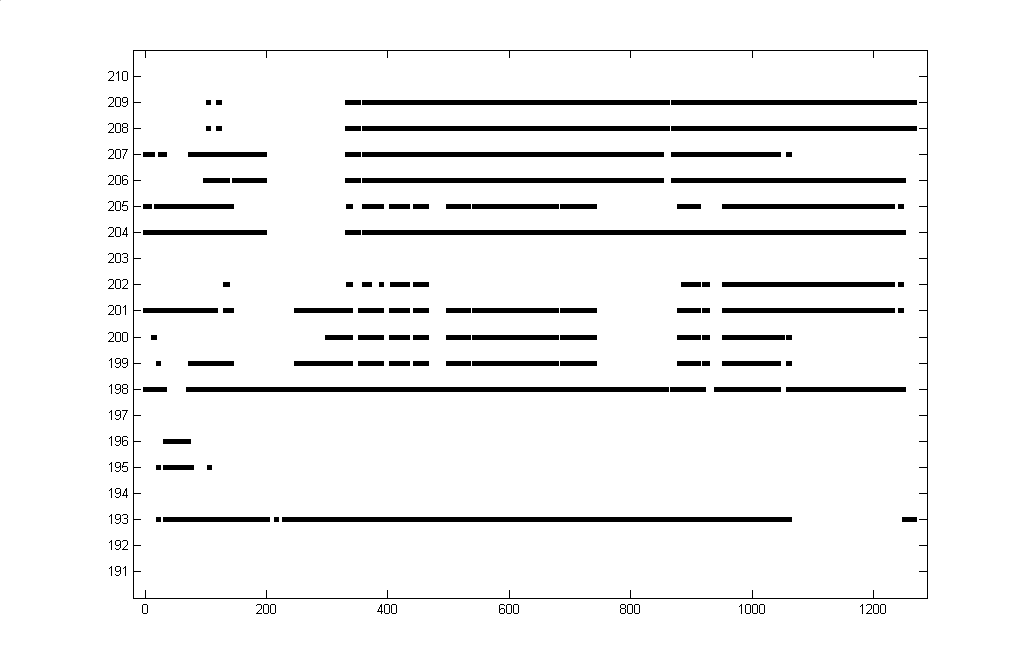 |
| h54AON1 (++)  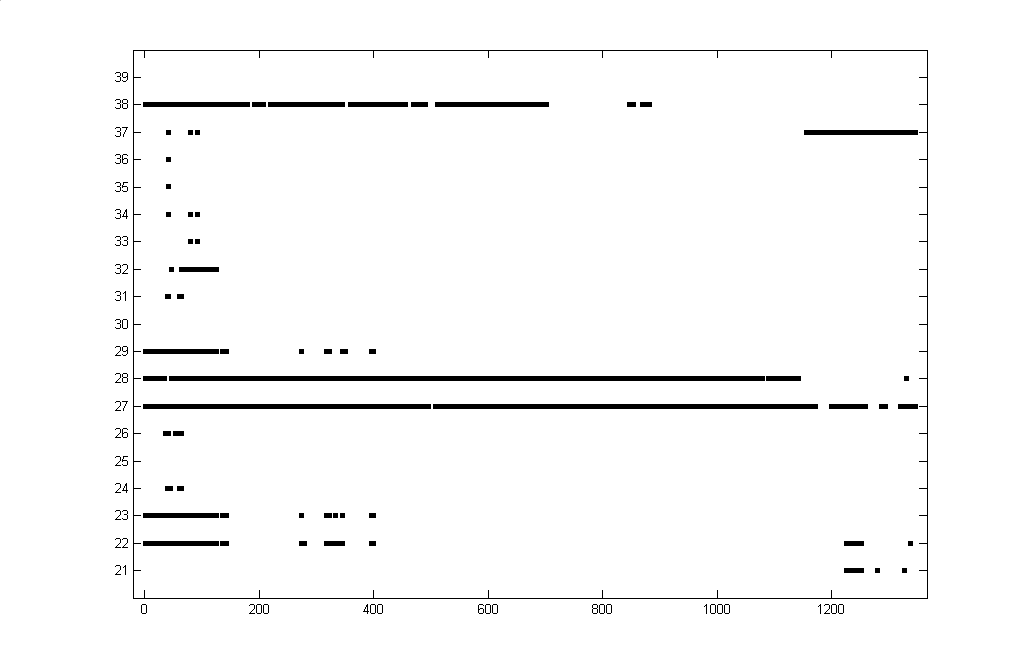 | h54AON2 (++)  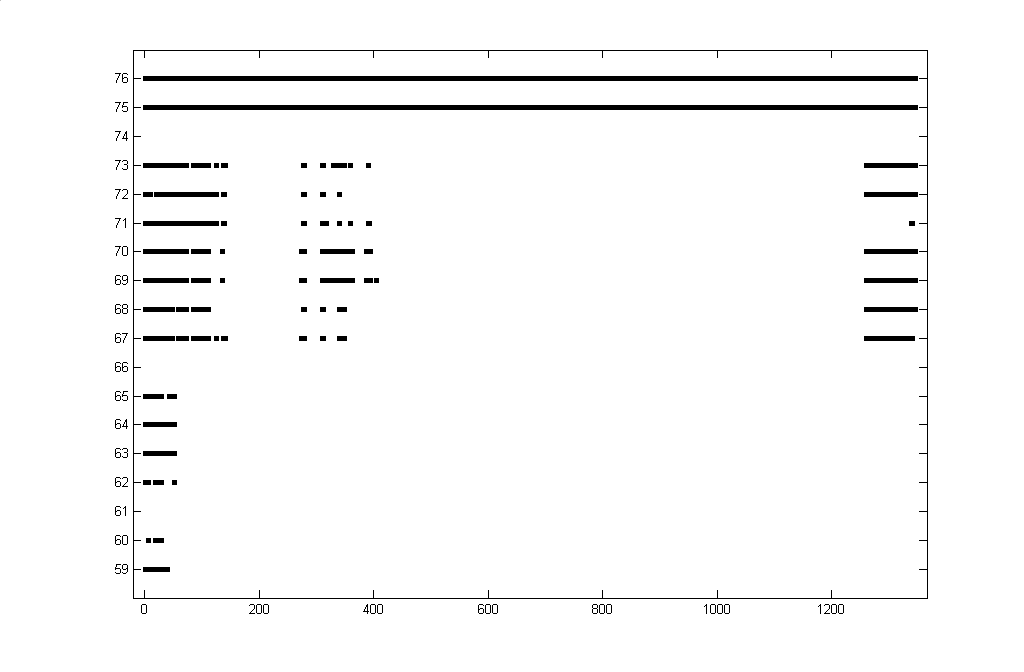 | h55AON5 (++)  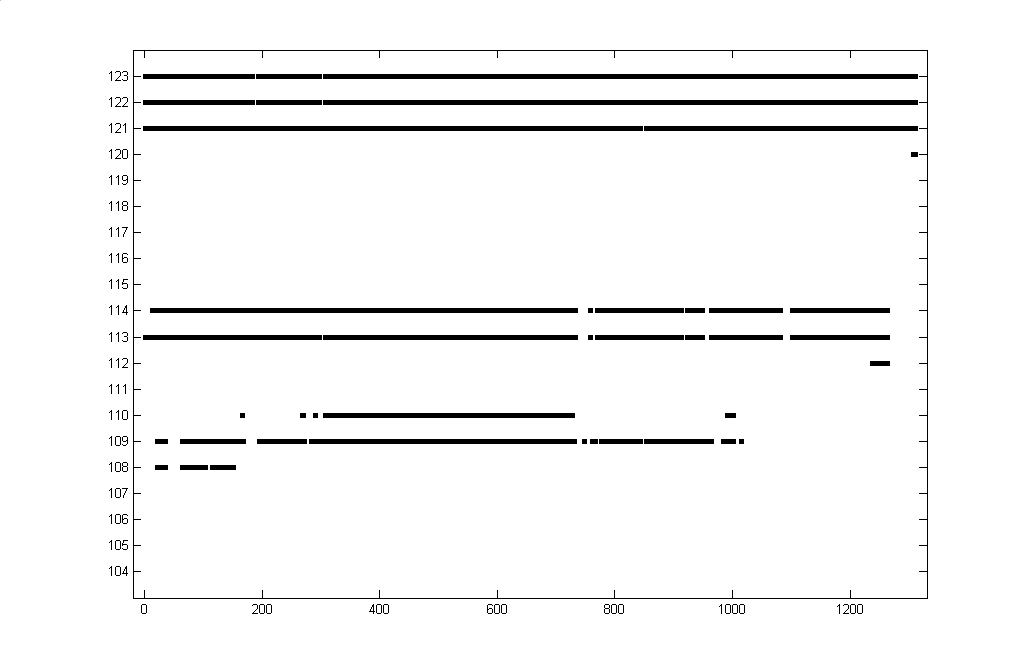 |
| h55AON6 (++)  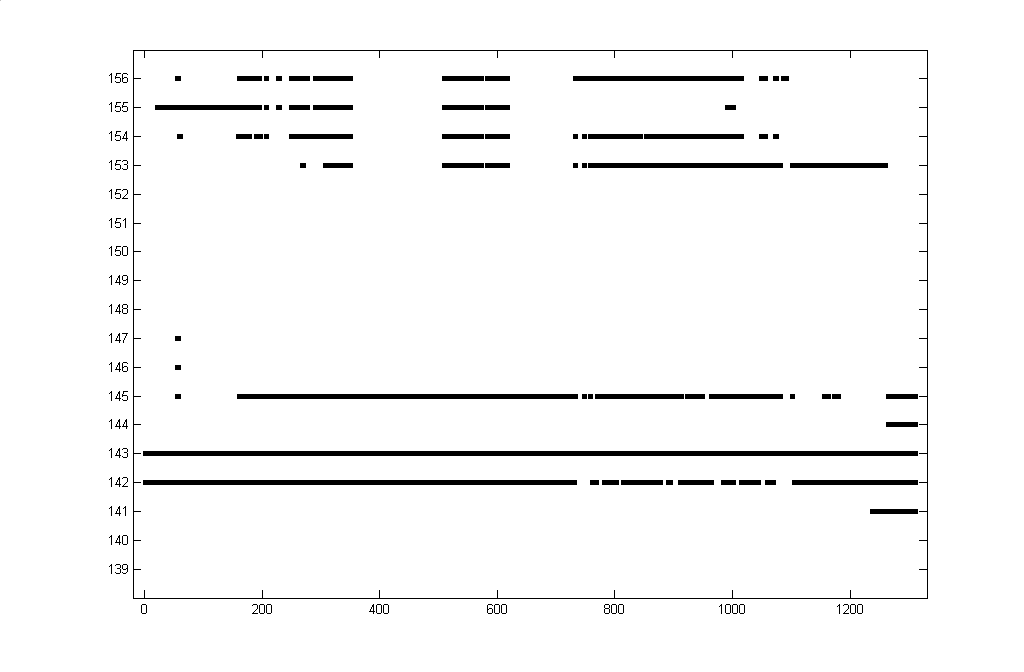 | h59AON2 (++)  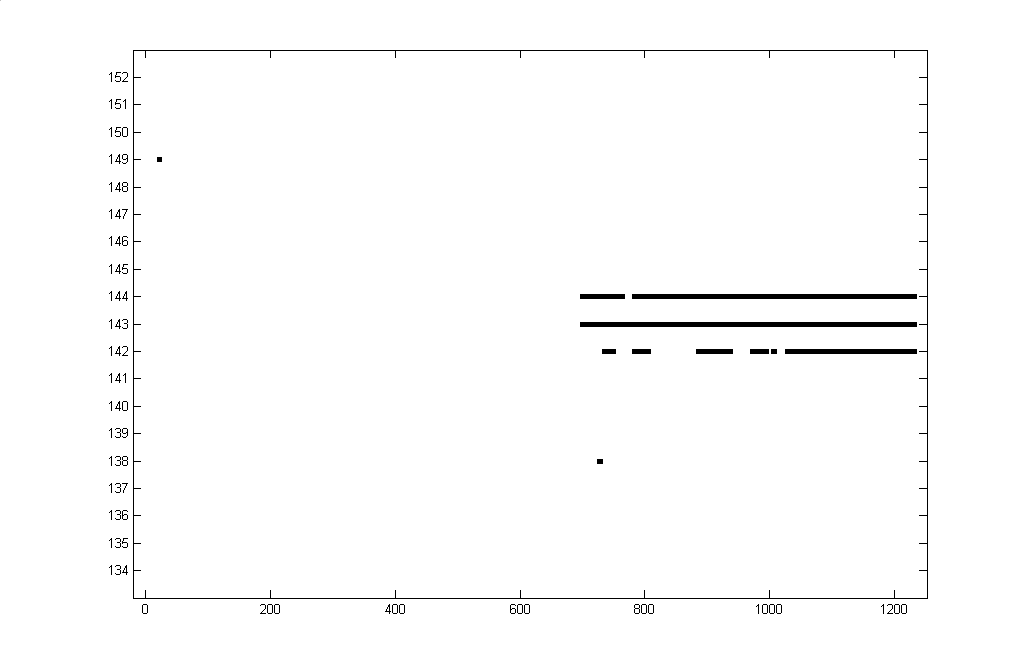 | h62AON1 (++)  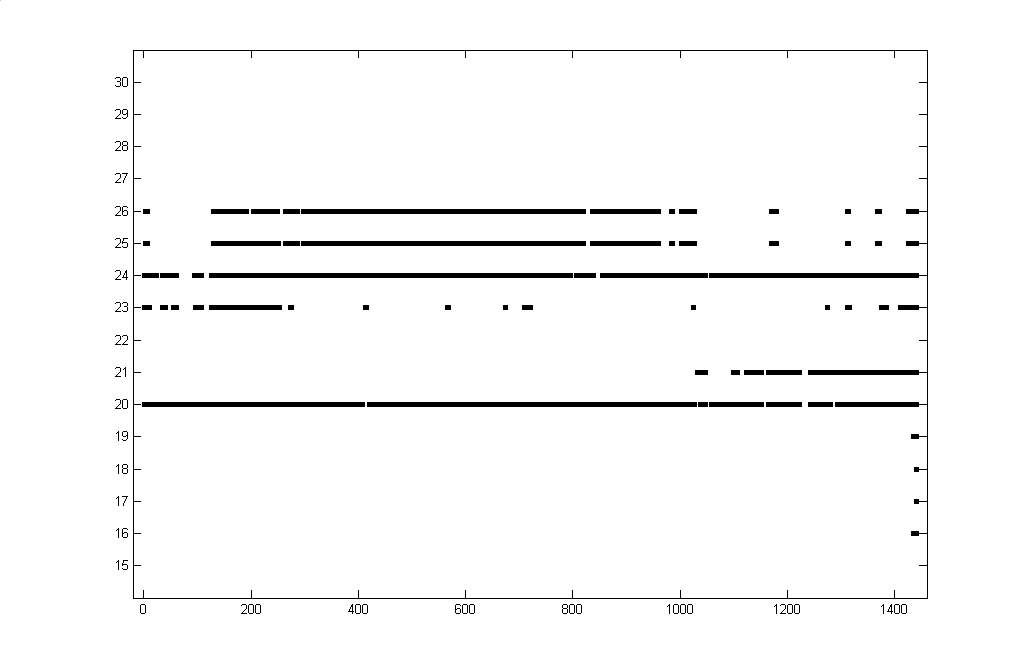 |
| h71AON1 (++)  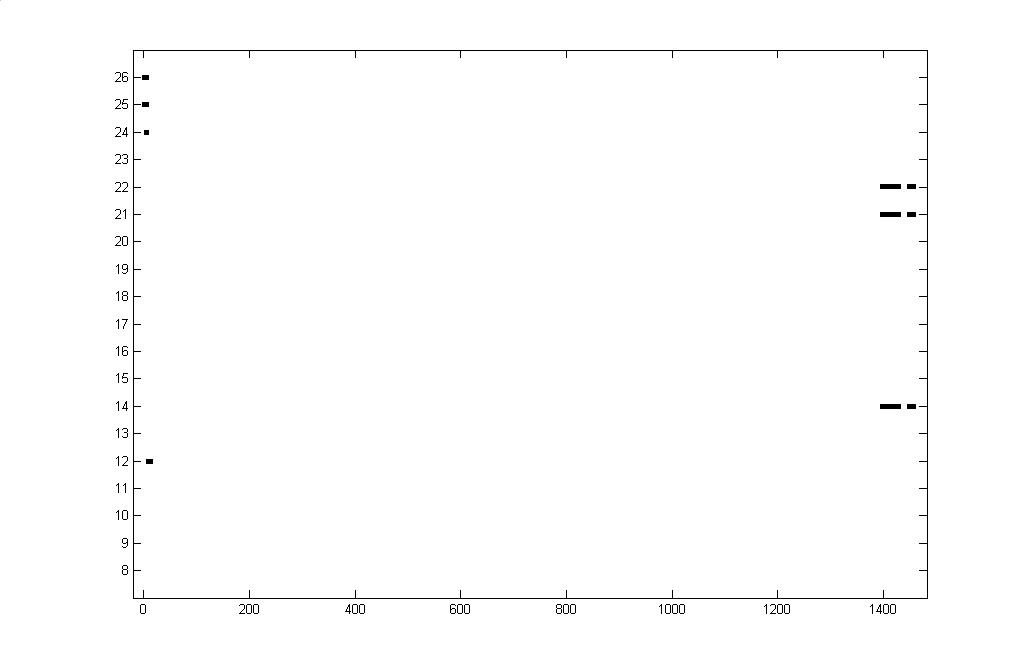 | h71AON2 (++)  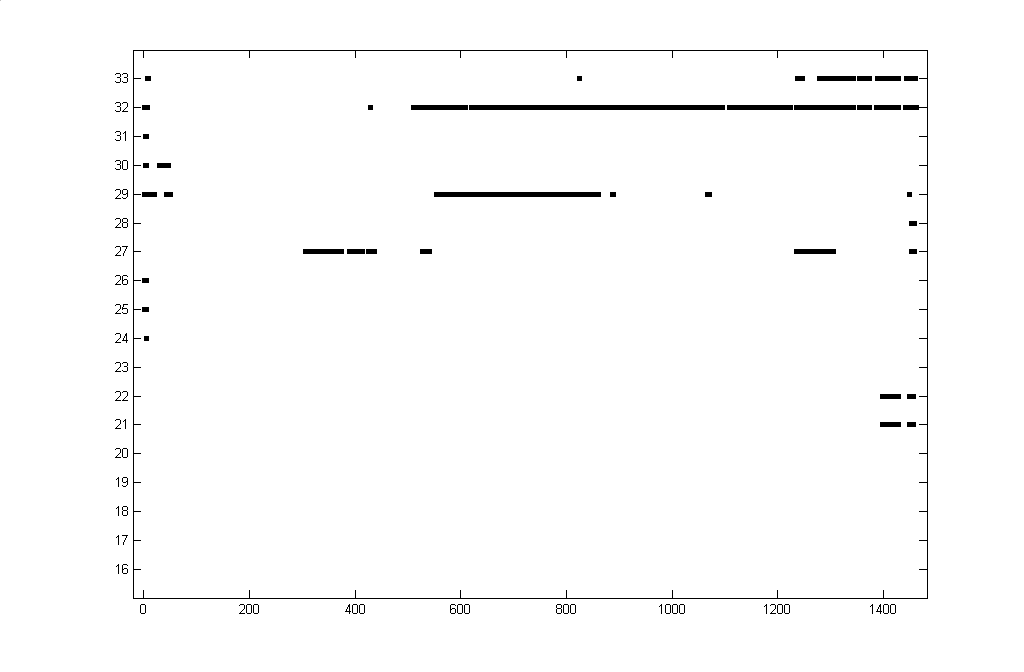 | h72AON1 (++)  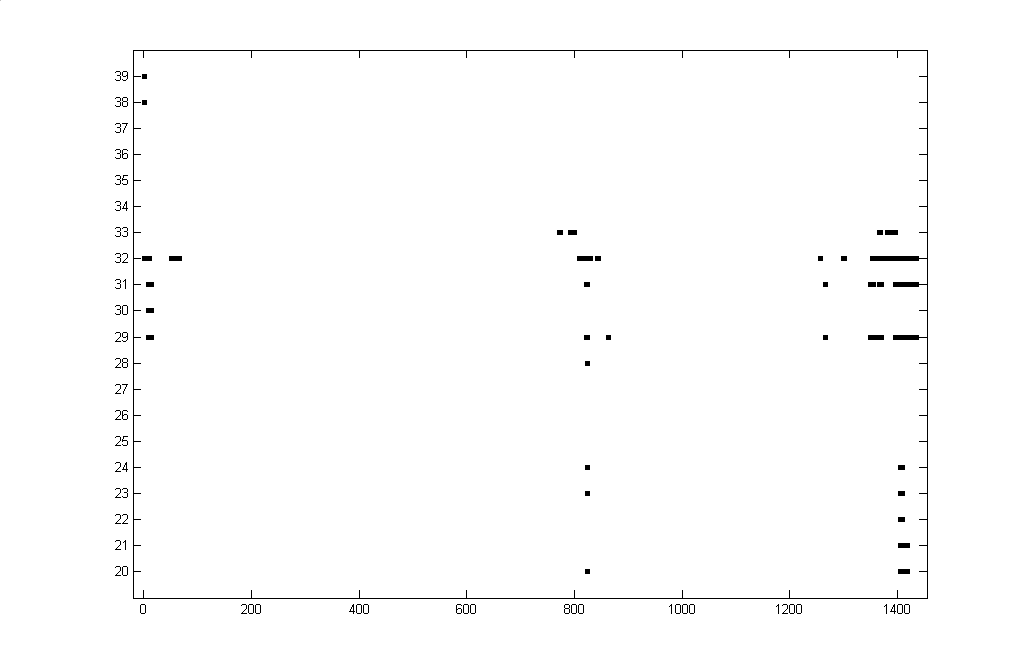 |
| h73AON1 (++)  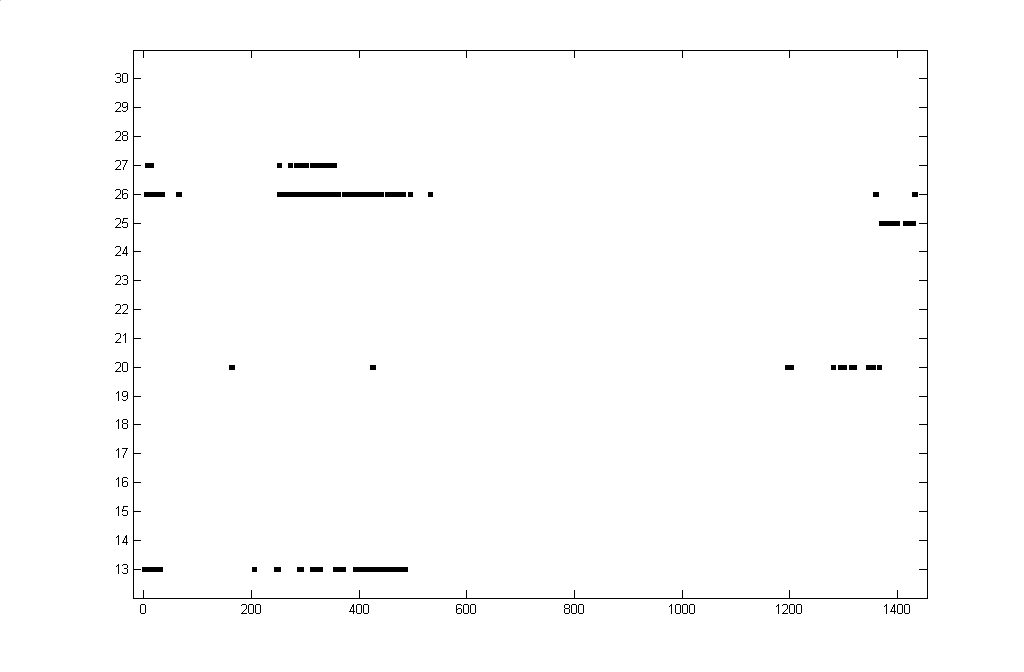 | h74AON1 (++)  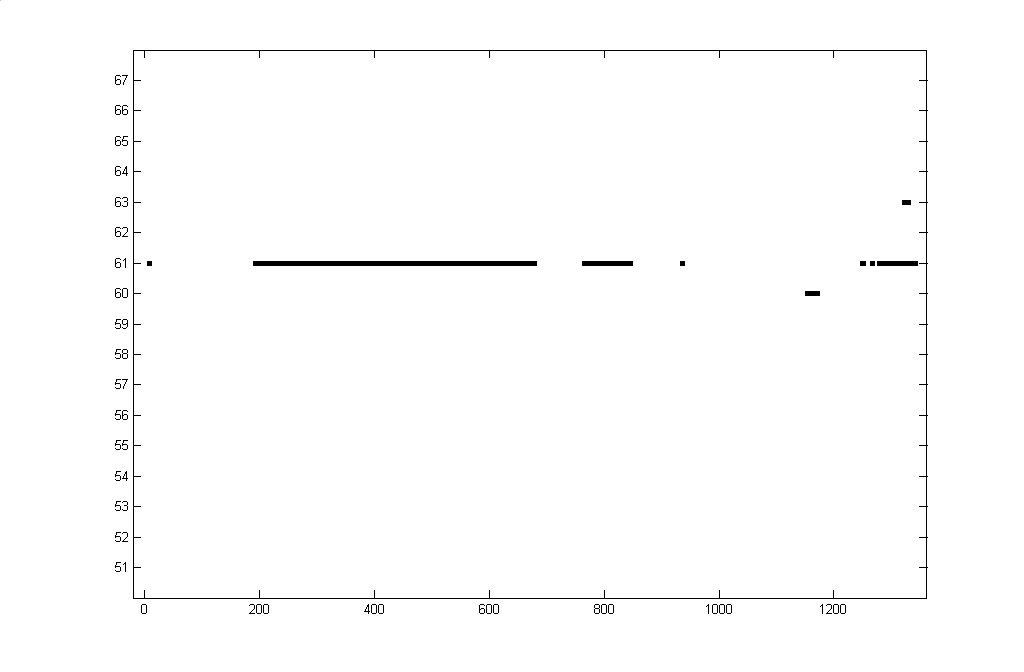 | h75AON1 (++)  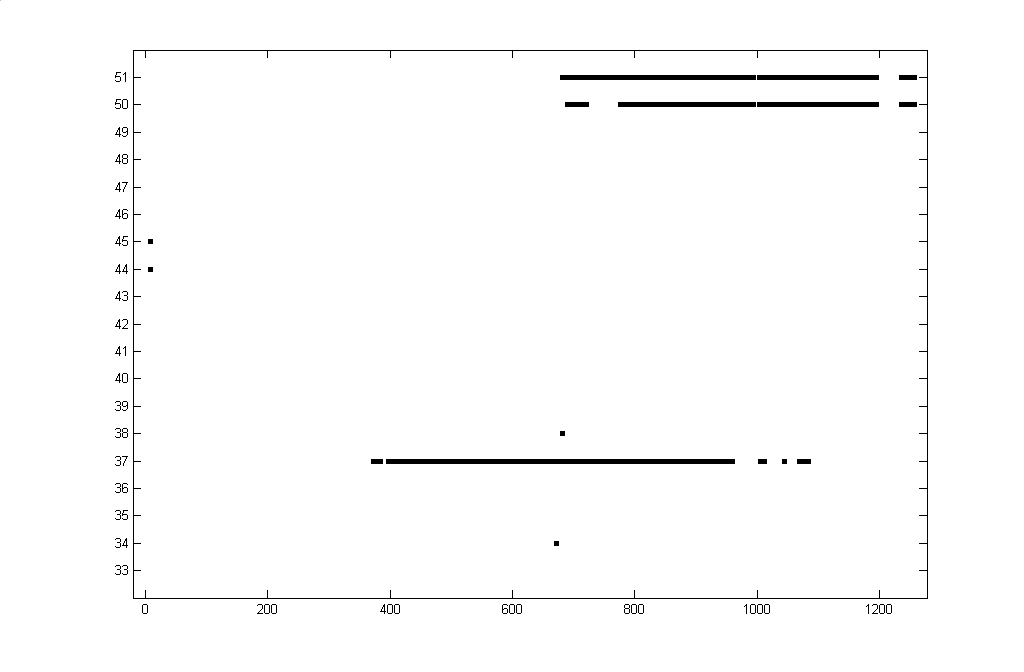 |
| h75AON2 (++)  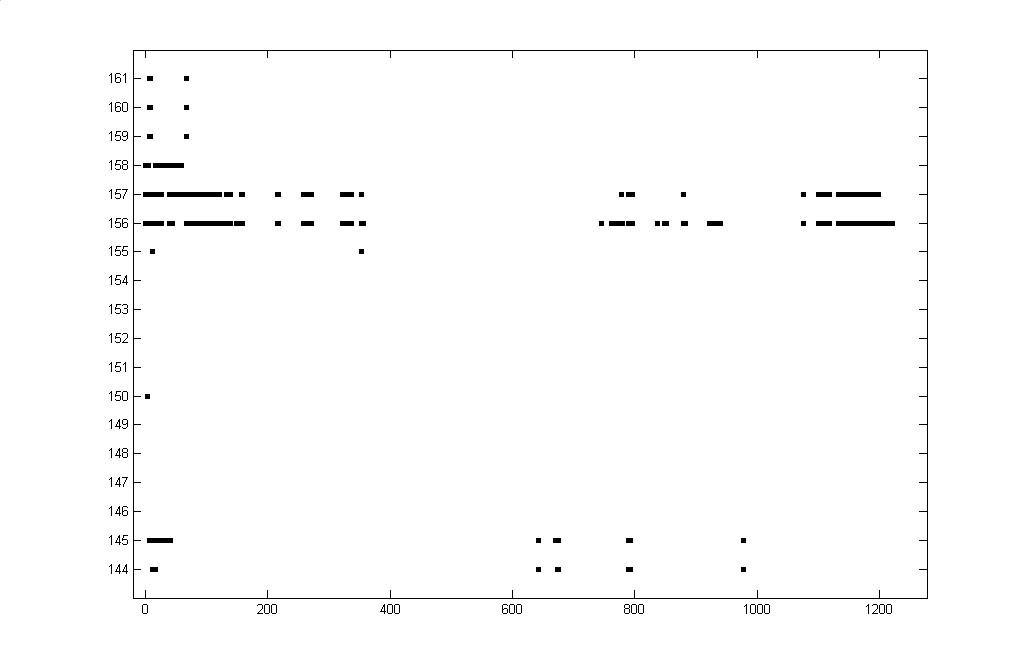 | h77AON1 (++)  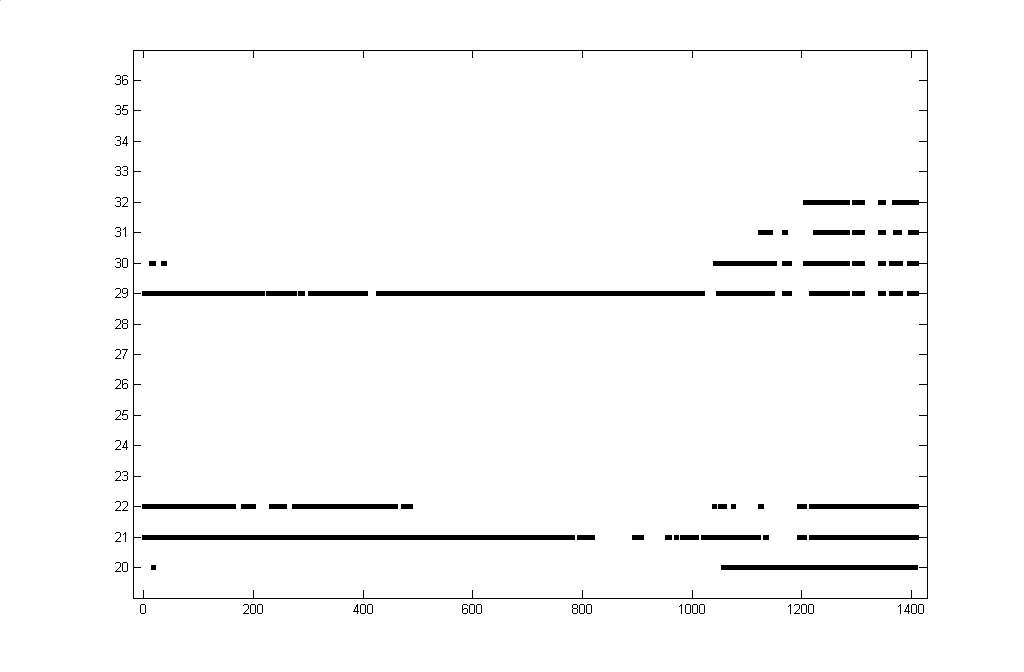 | h77AON2 (++)  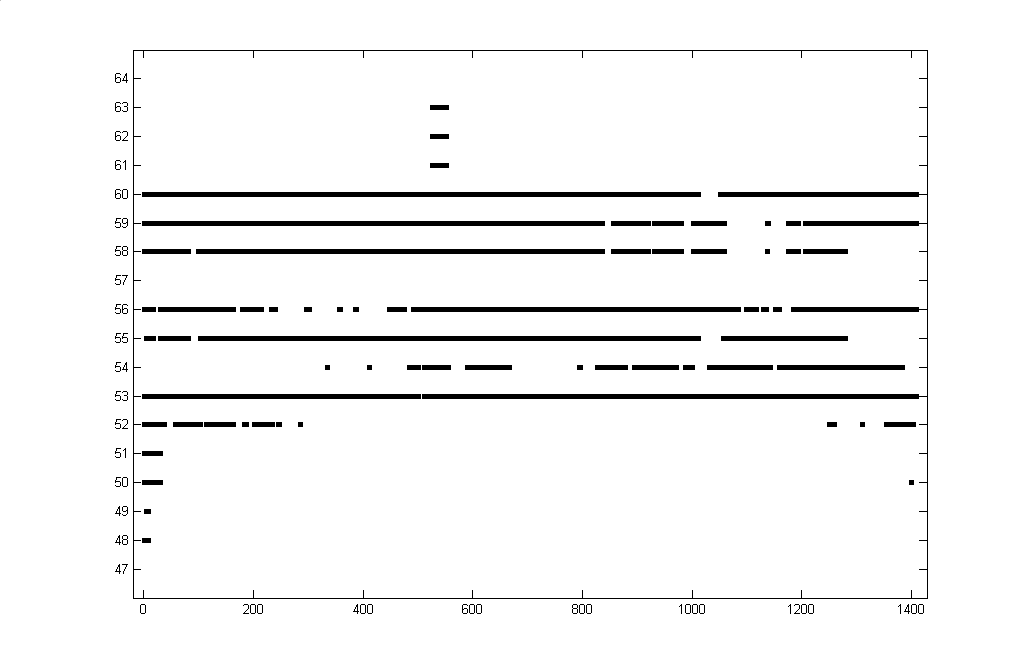 |
| h78AON1 (++)  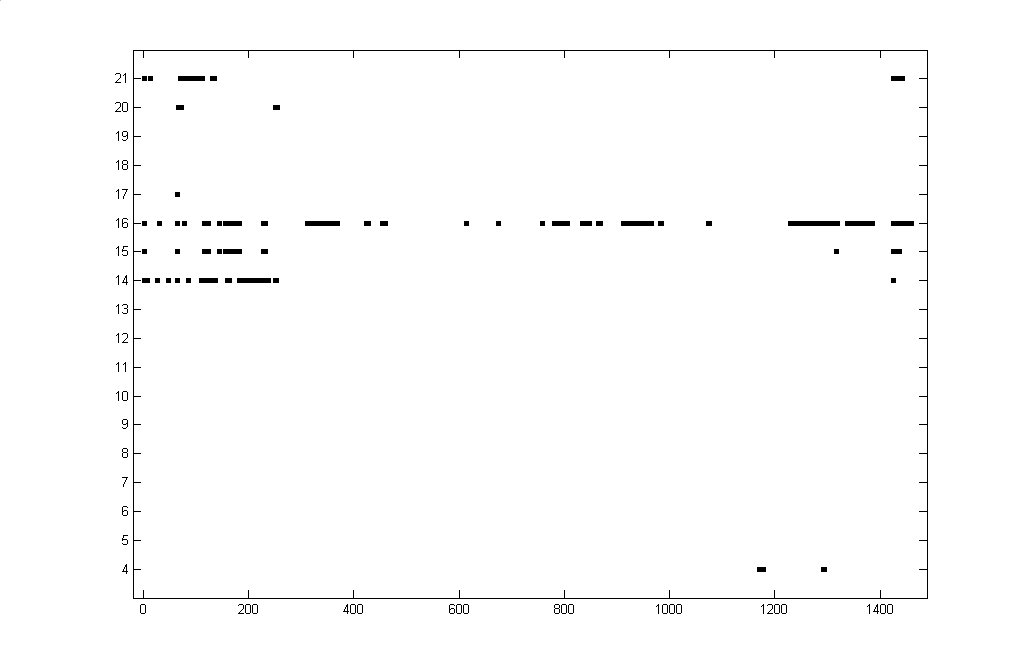 | h78AON2 (++)  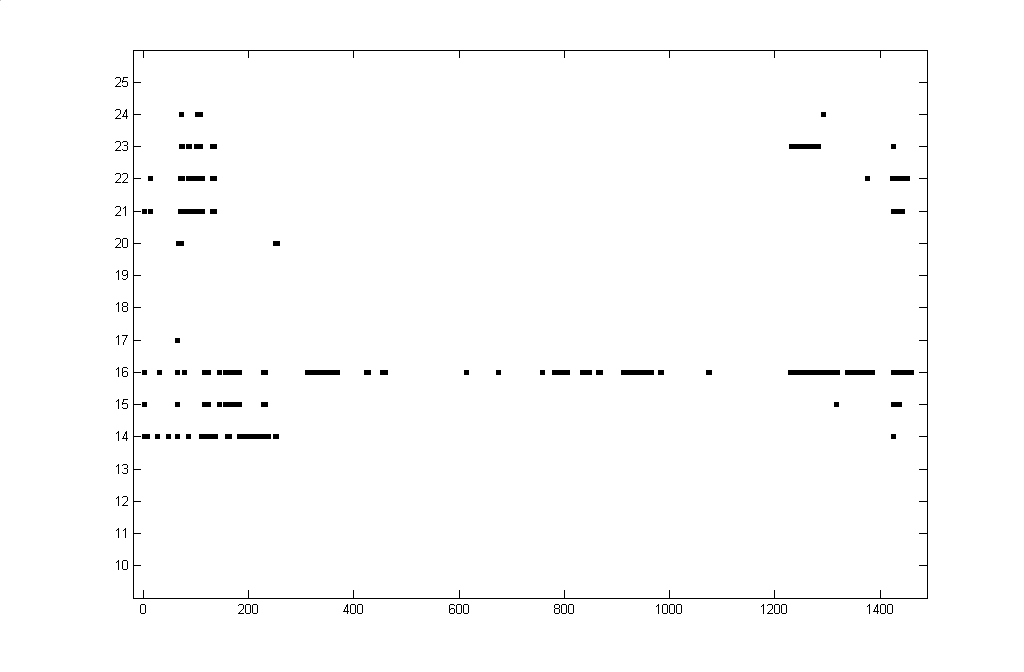 | h17AON2 (+)  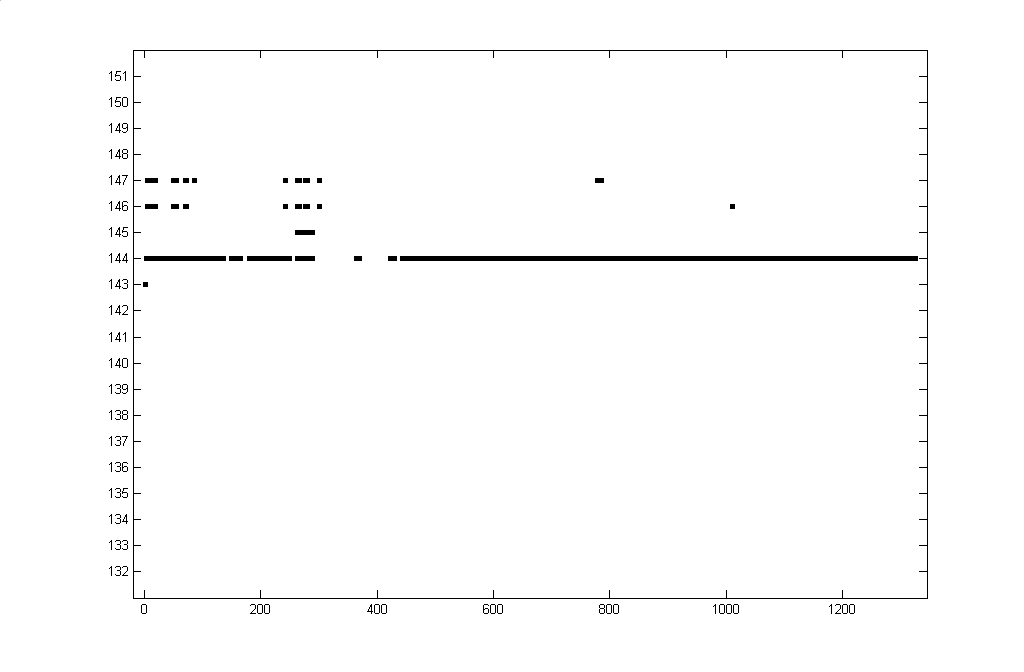 |
| h19AON (+)  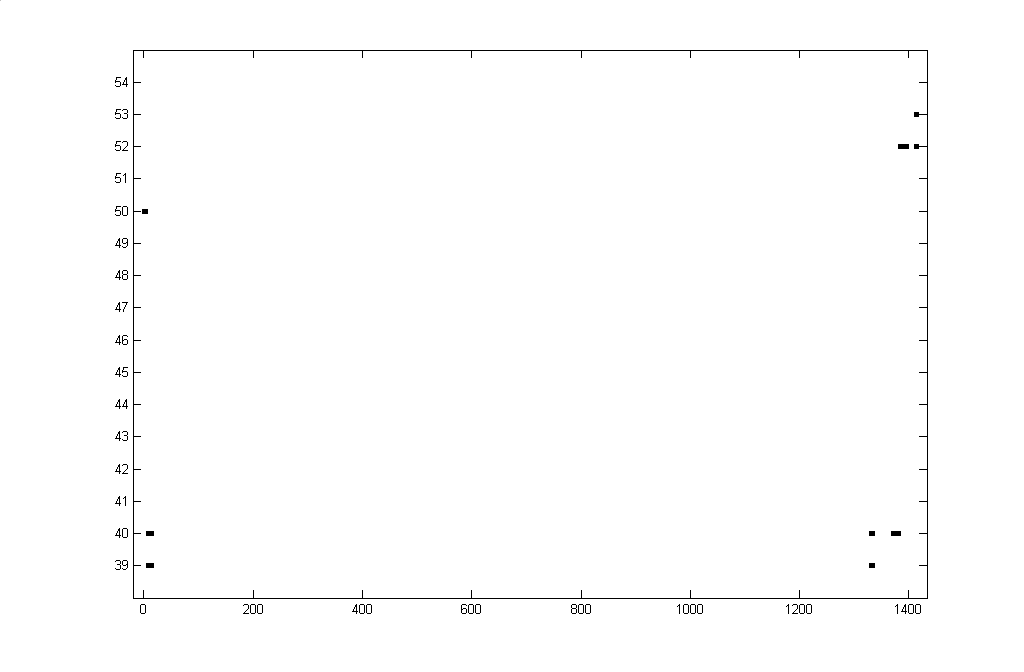 | h29AON9 (+)  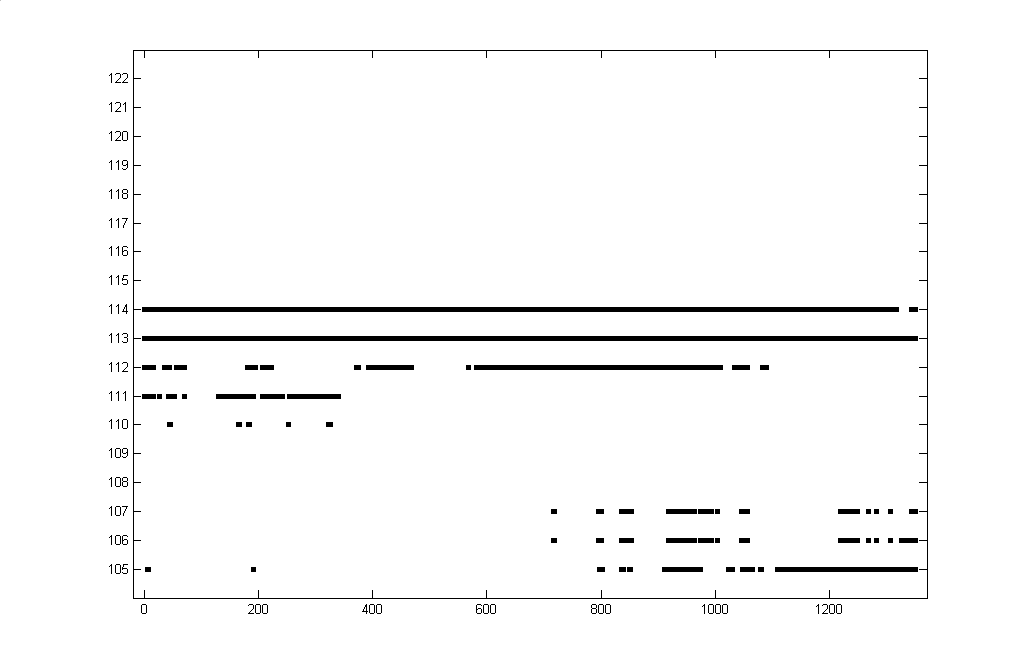 | h29AON11 (+)  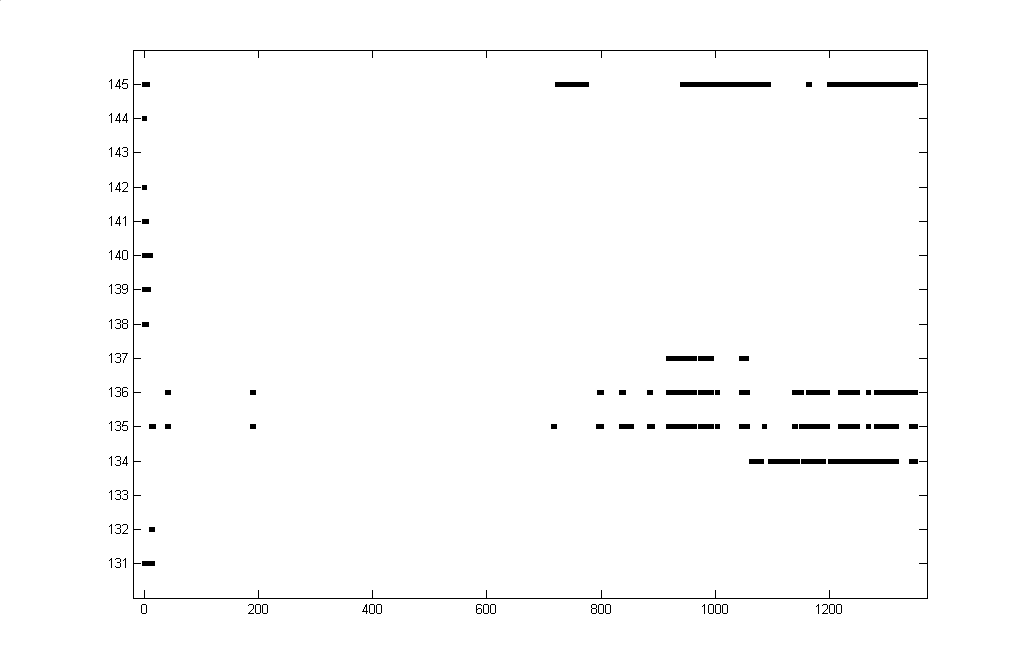 |
| h41AON2 (+)  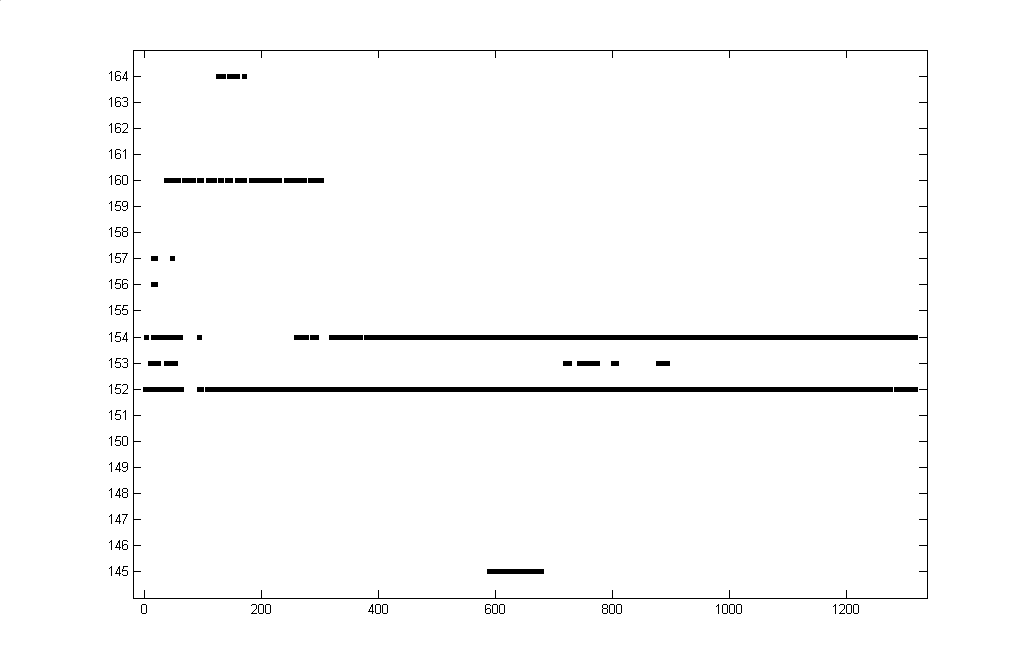 | h42AON1 (+)  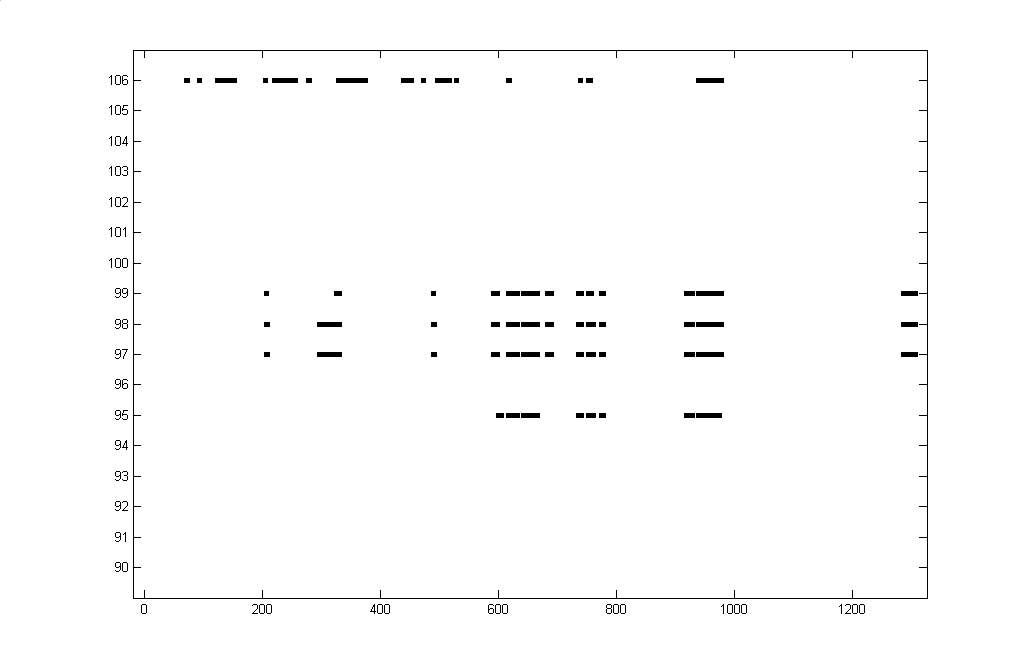 | h42AON2 (+)  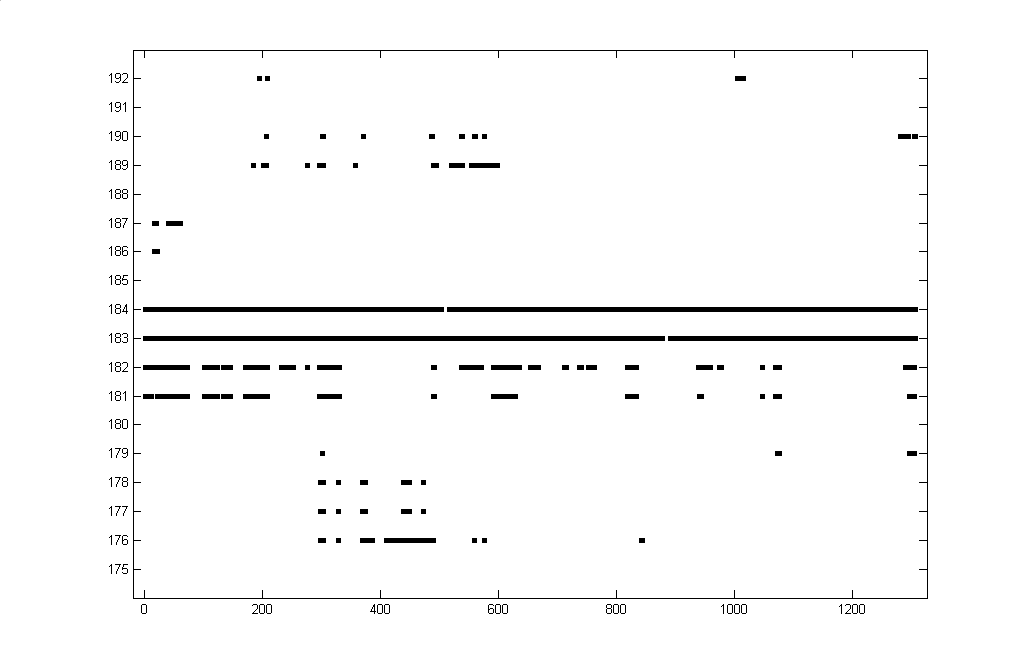 |
| h43AON2 (+)  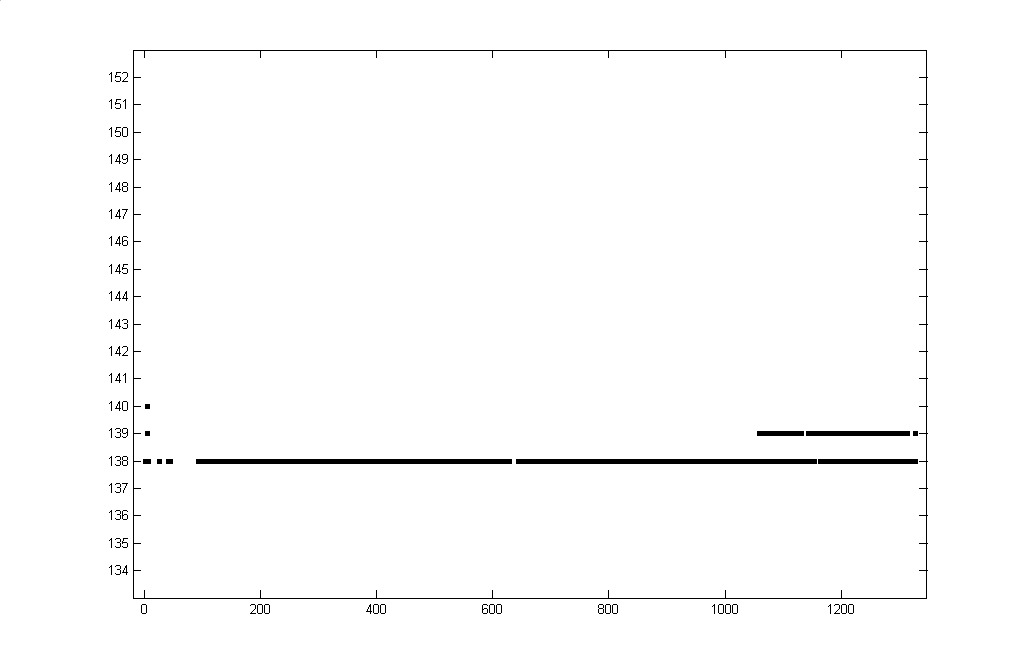 | h45AON5 (+)  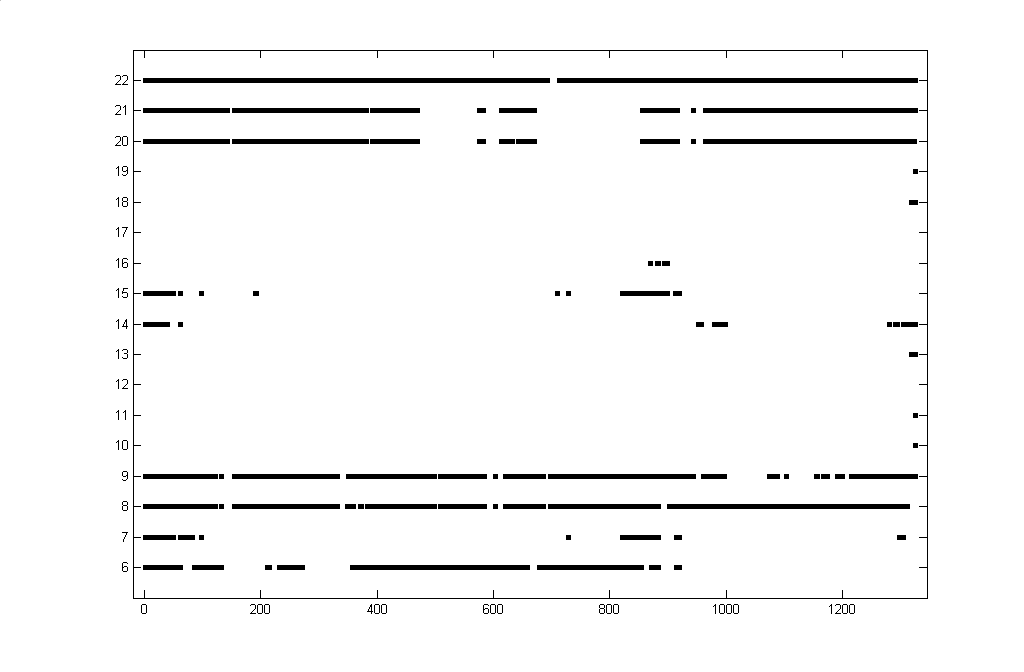 | h46AON4 (+)  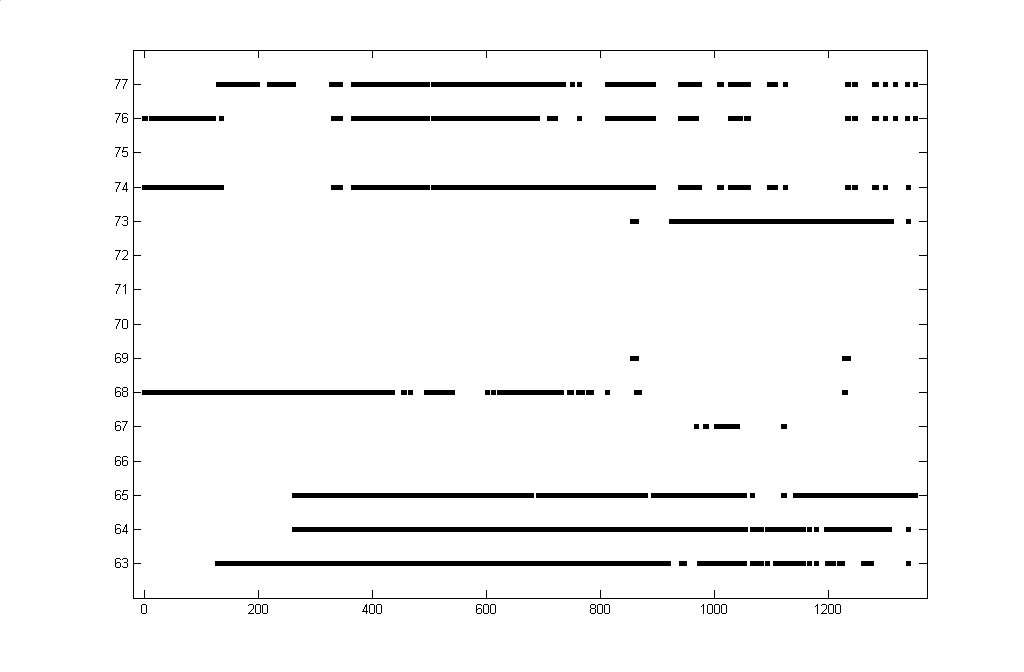 |
| h46AON6 (+)  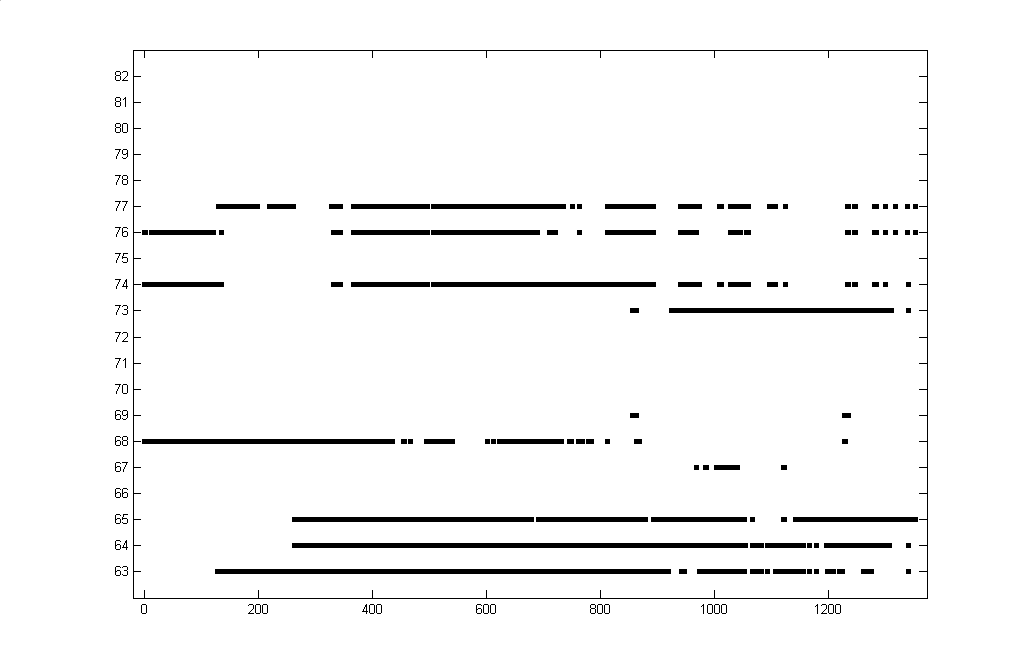 | h46AON20 (+)  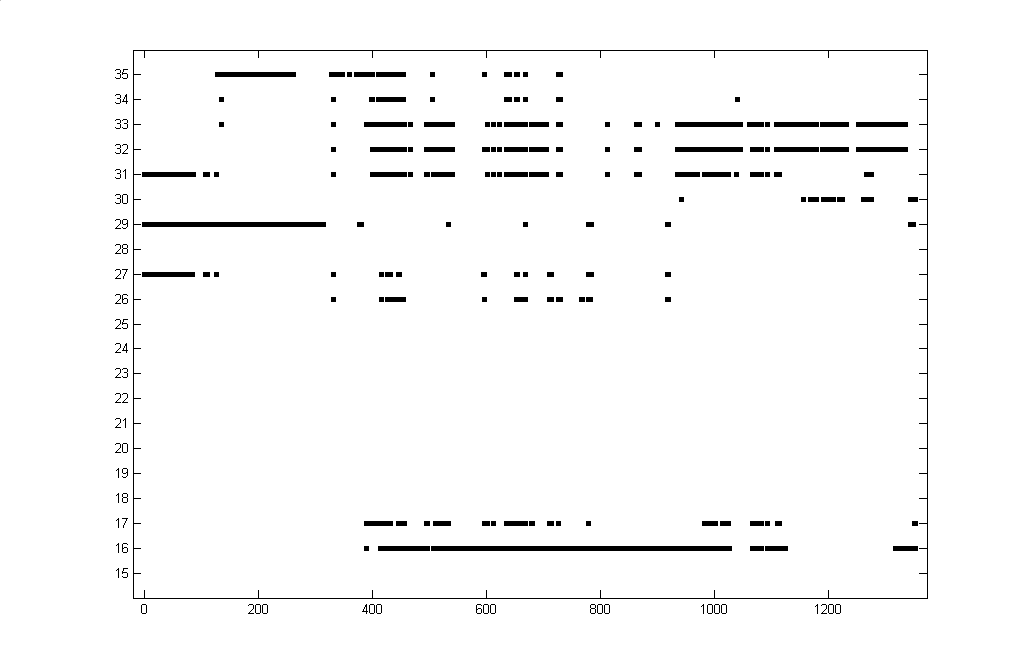 | h46AON24 (+)  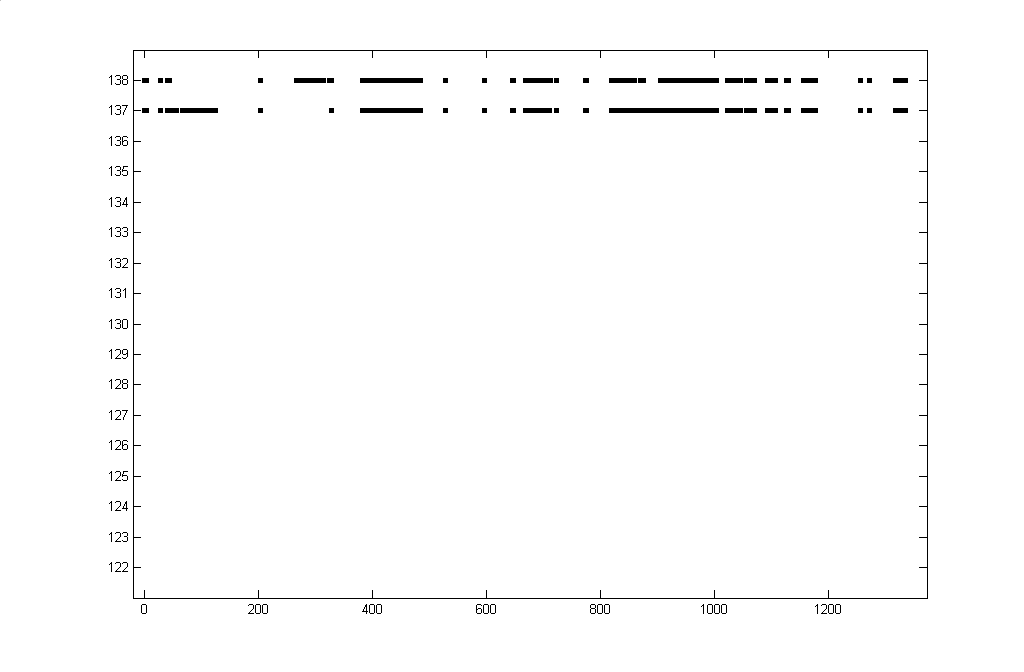 |
| h46AON25 (+)  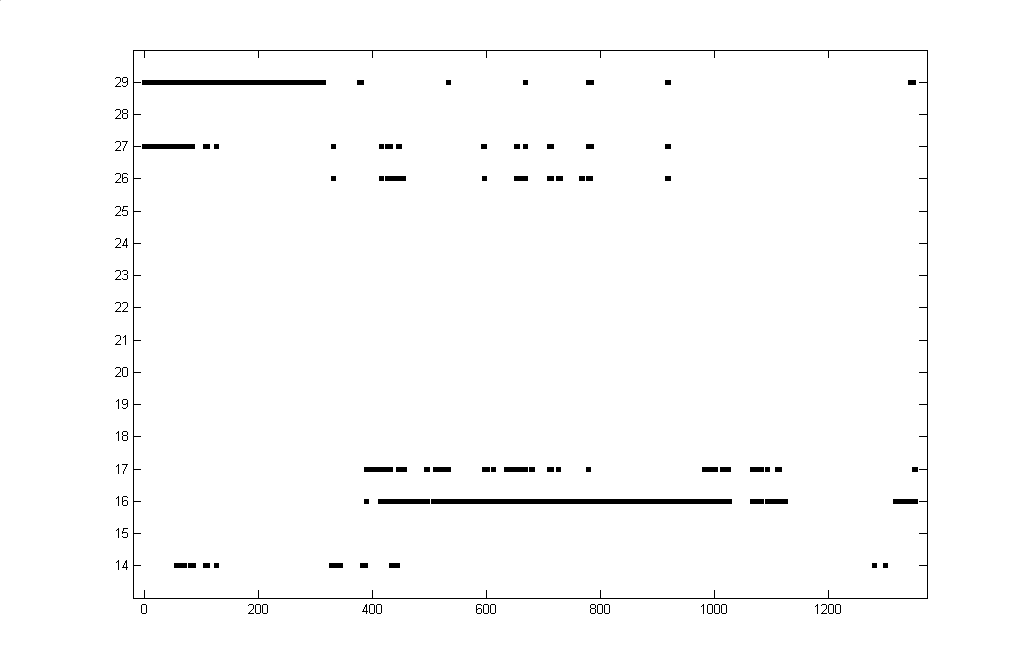 | h48AON6 (+)  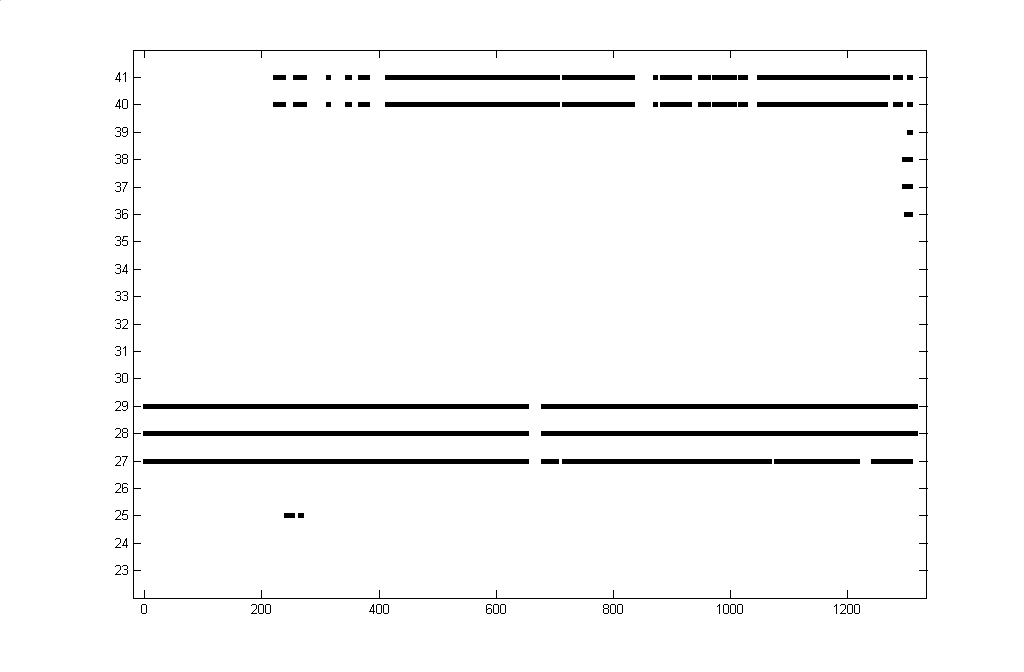 | h48AON7 (+)  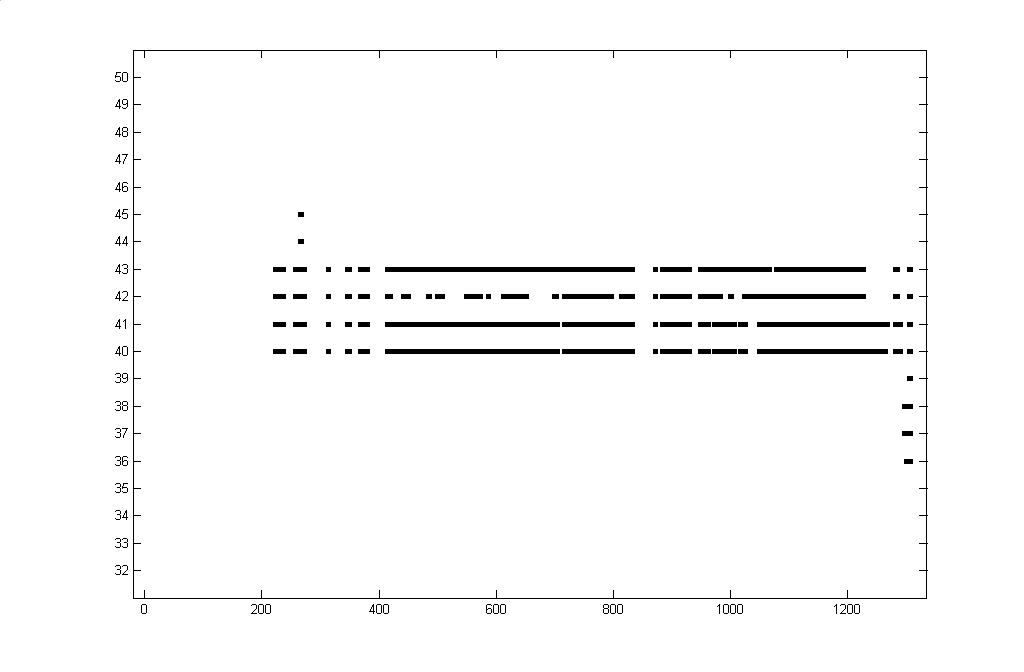 |
| h48AON9 (+)  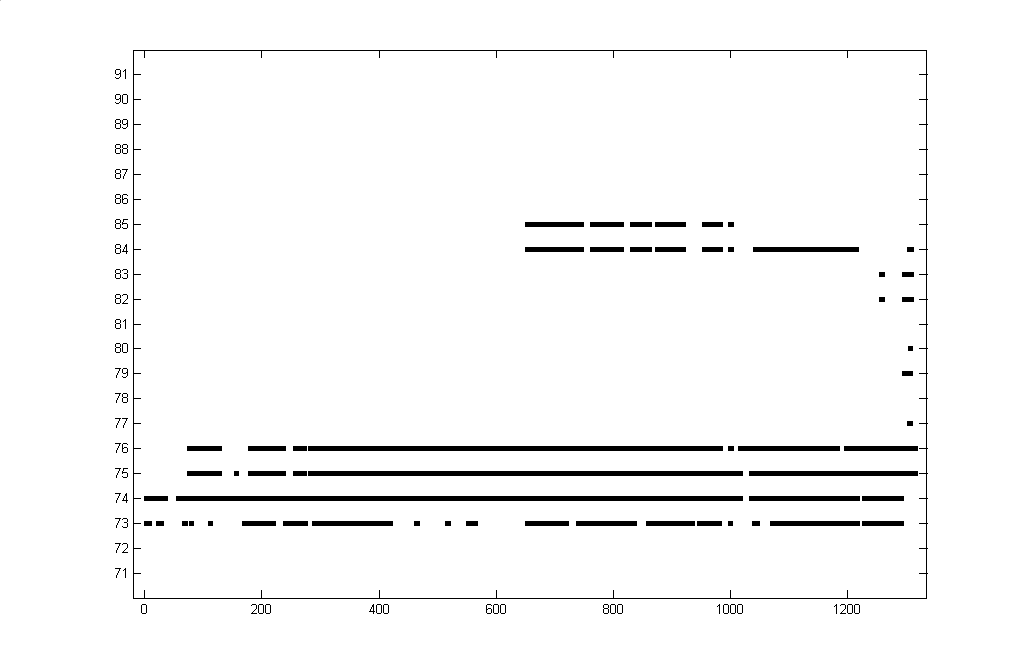 | h48AON10 (+)  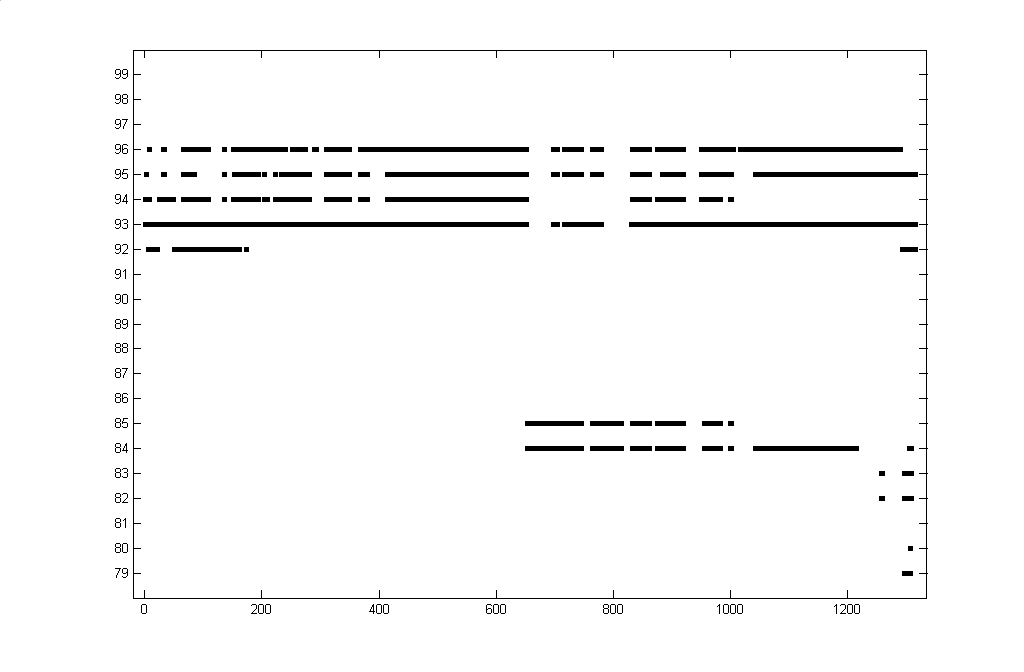 | h50AON2 (+)  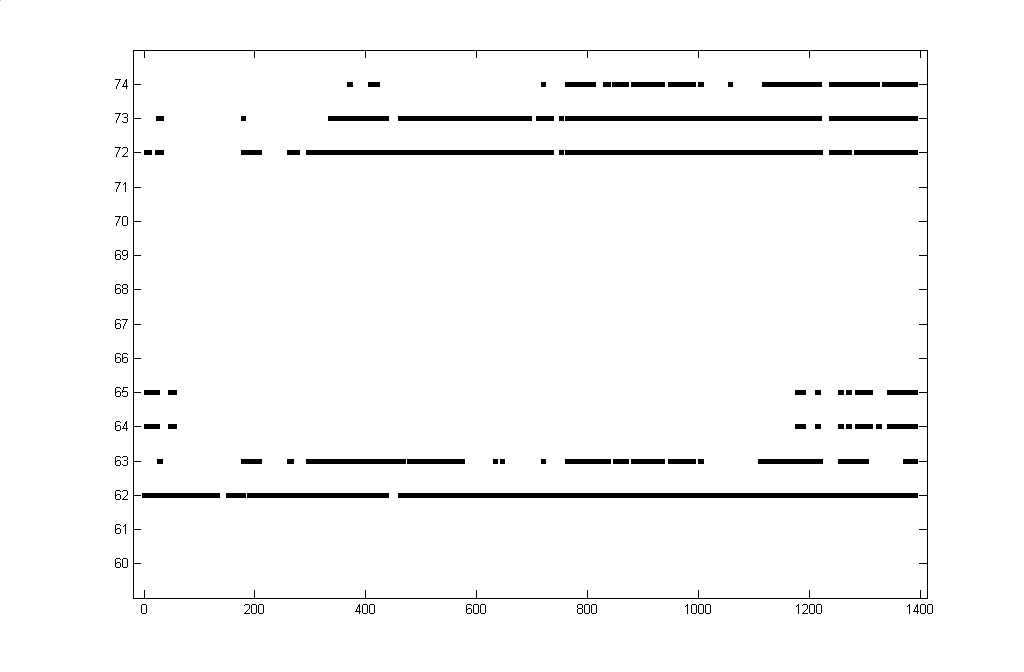 |
| h52AON1 (+)  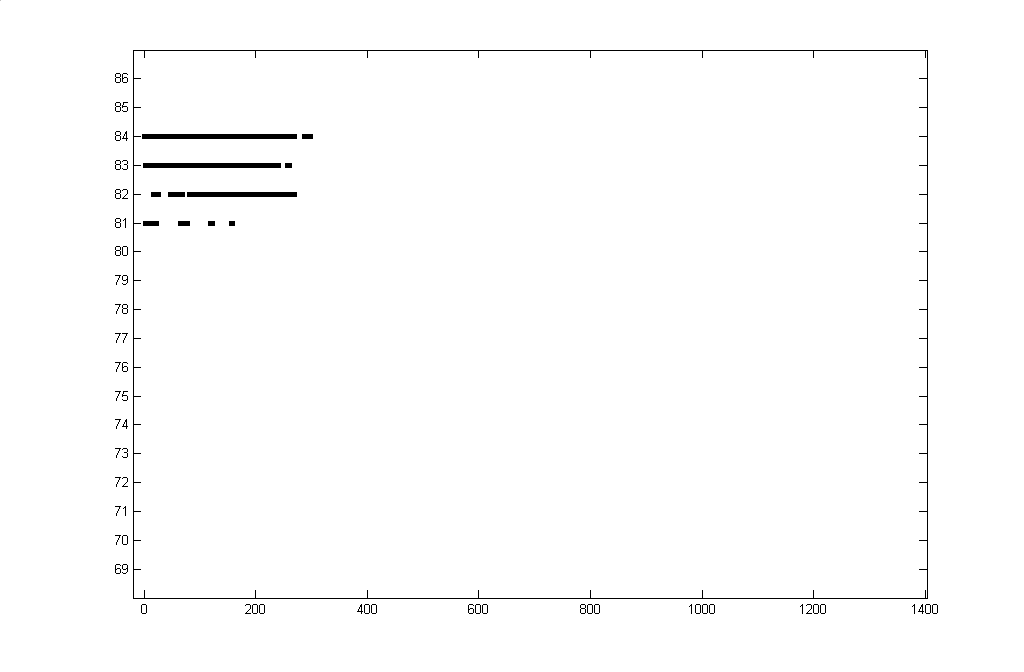 | h53AON1 (+)  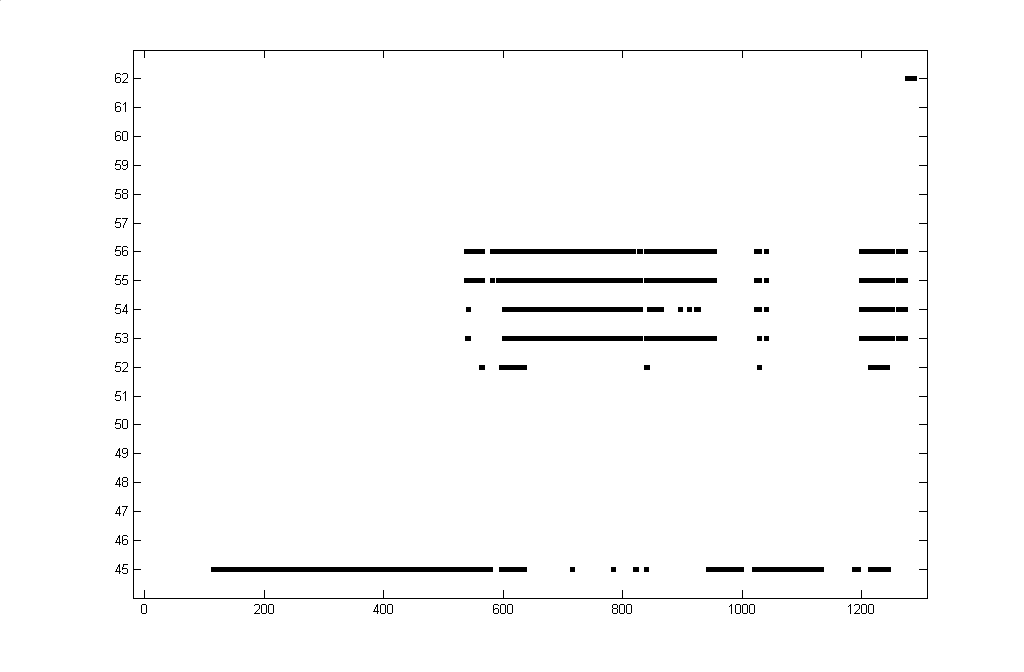 | h55AON1 (+)  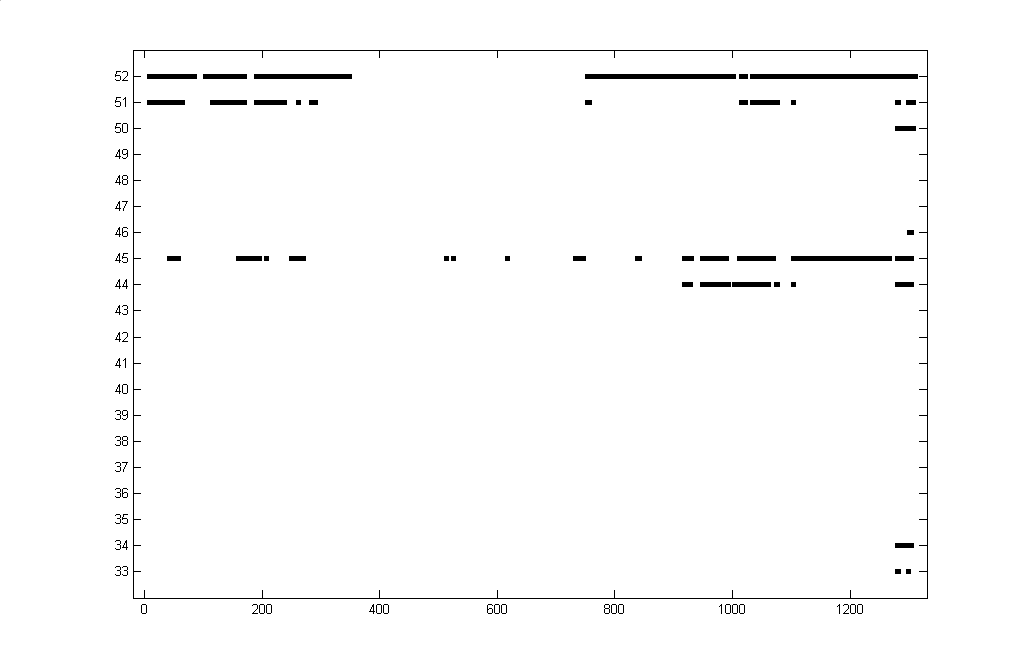 |
| h55AON2 (+)  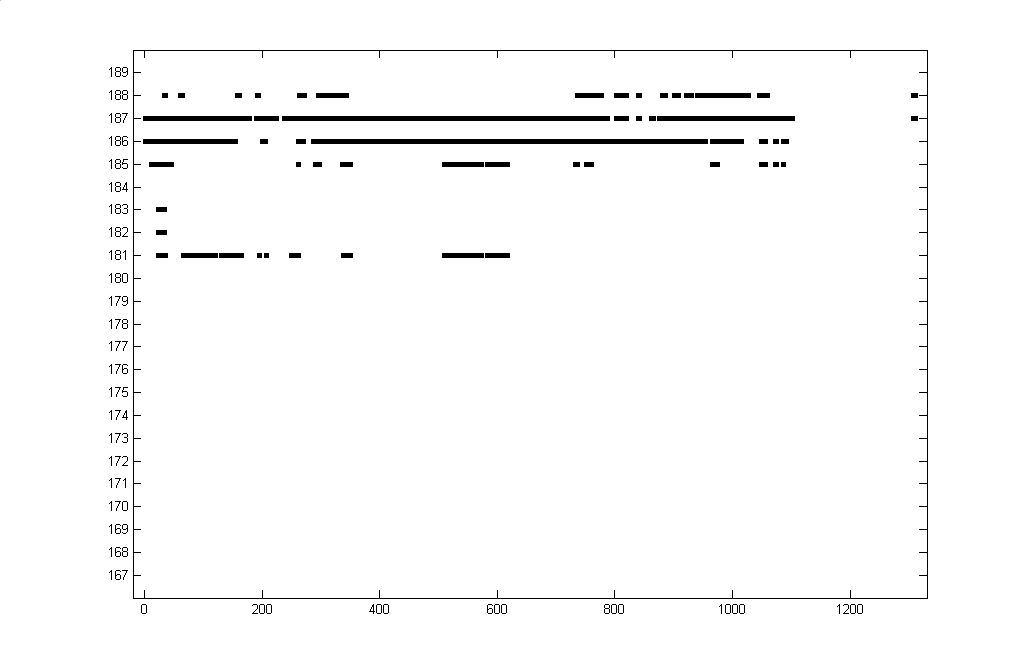 | h55AON3 (+)  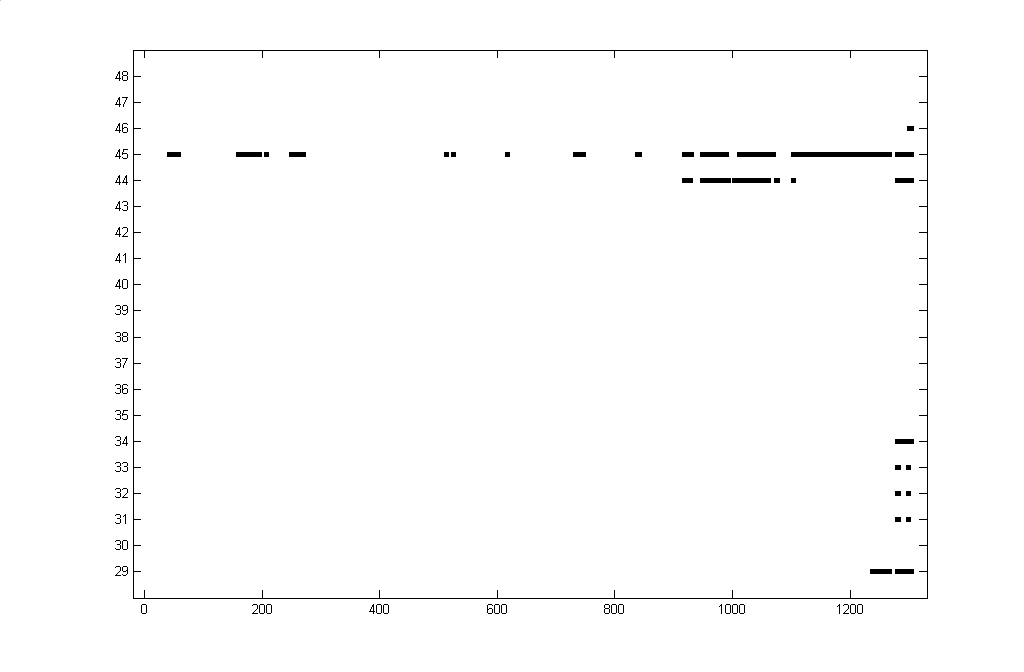 | h56AON1 (+)  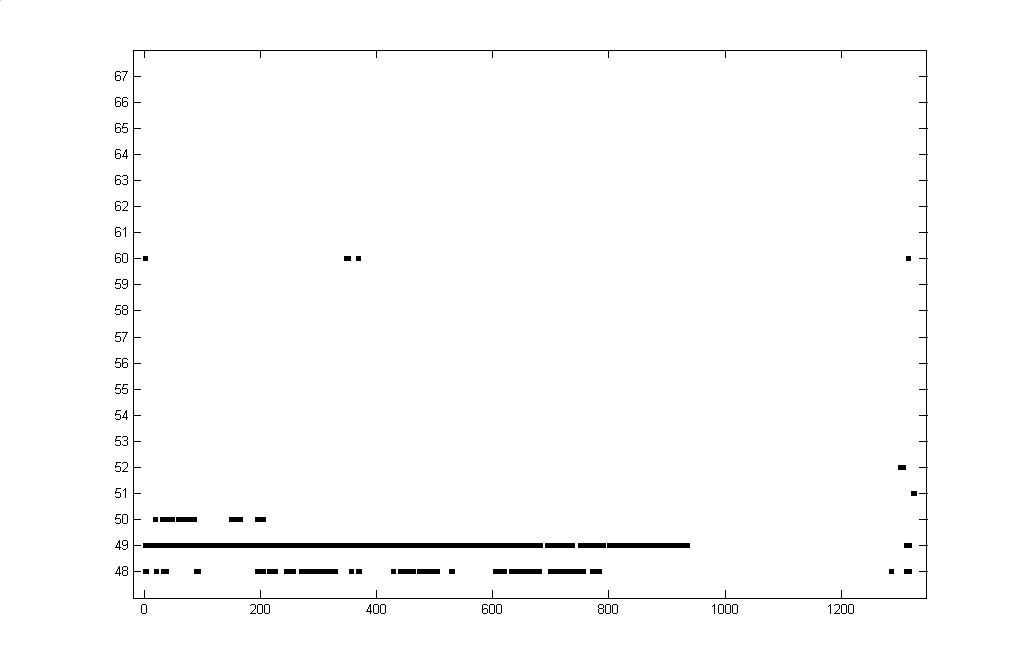 |
| h56AON3 (+)  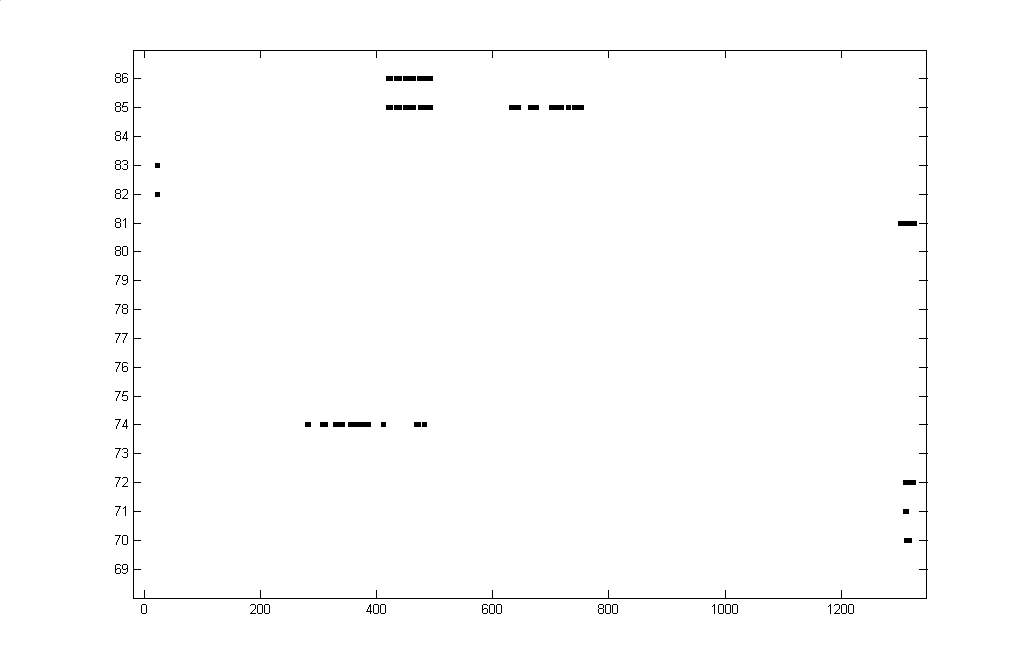 | h58AON2 (+)  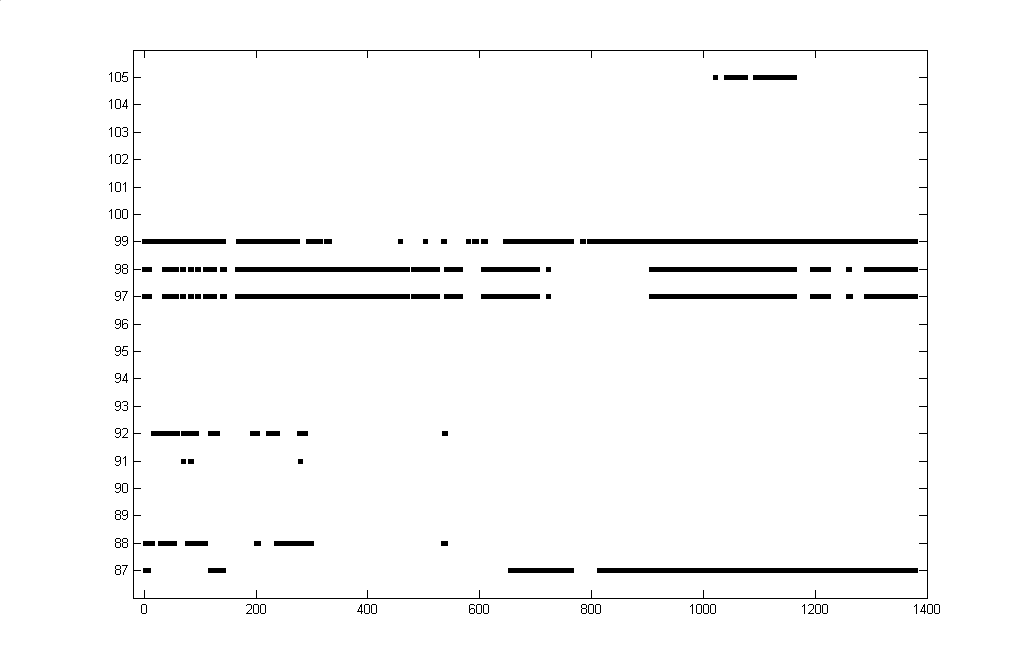 | h60AON1 (+)  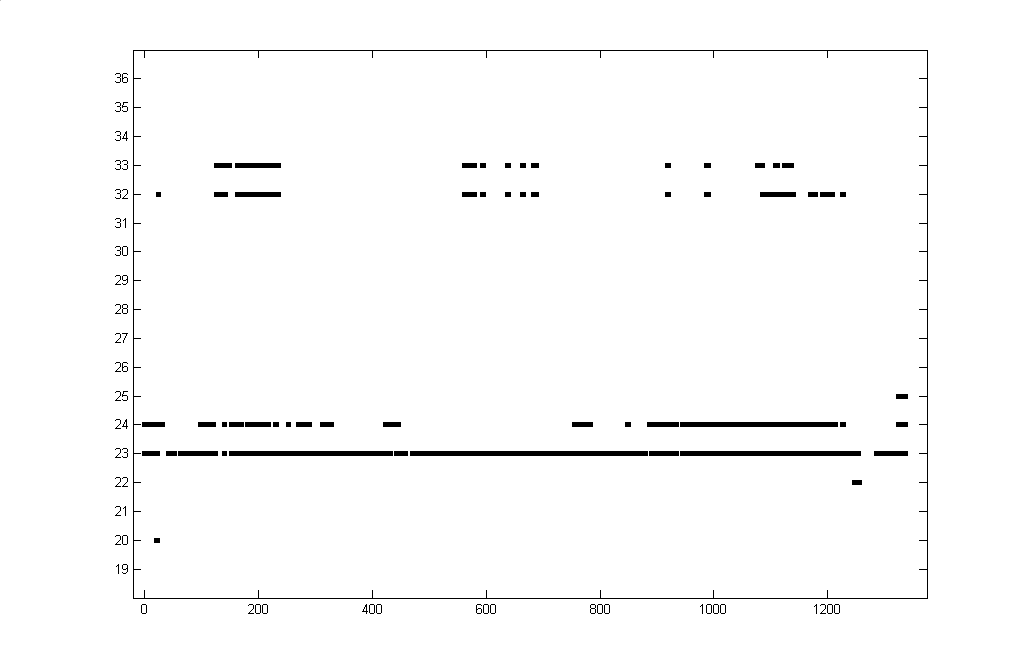 |
| h61AON2 (+)  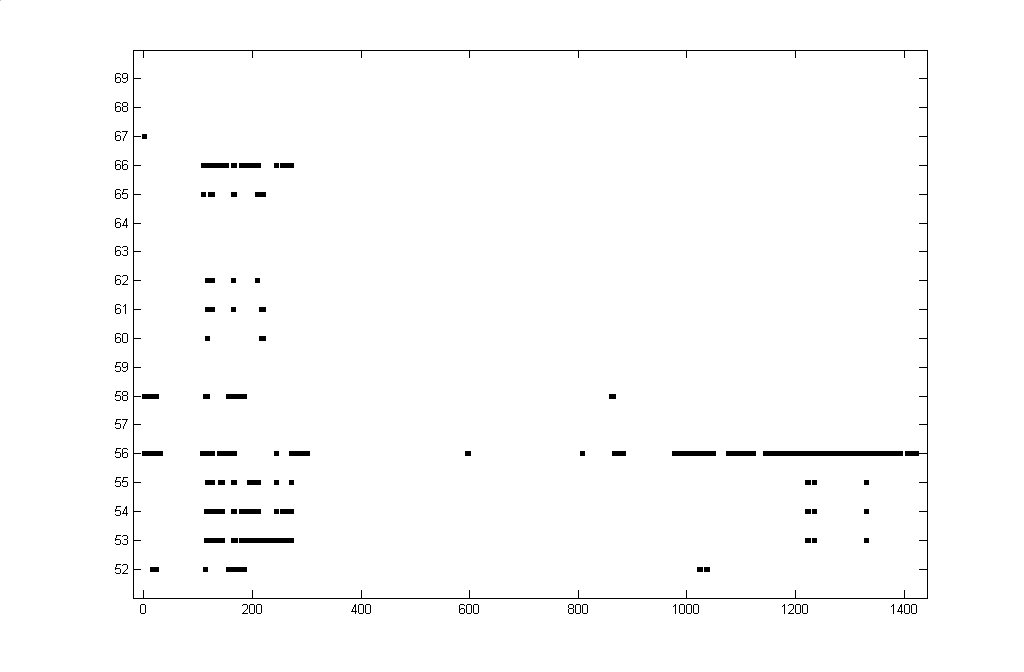 | h63AON1 (+)  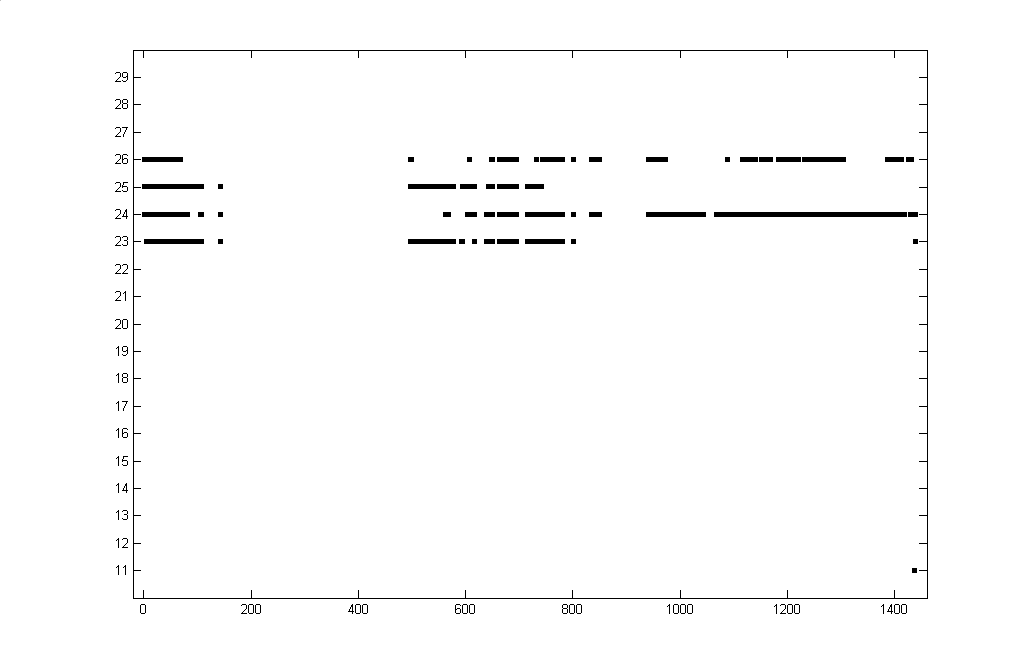 | h63AON2 (+)  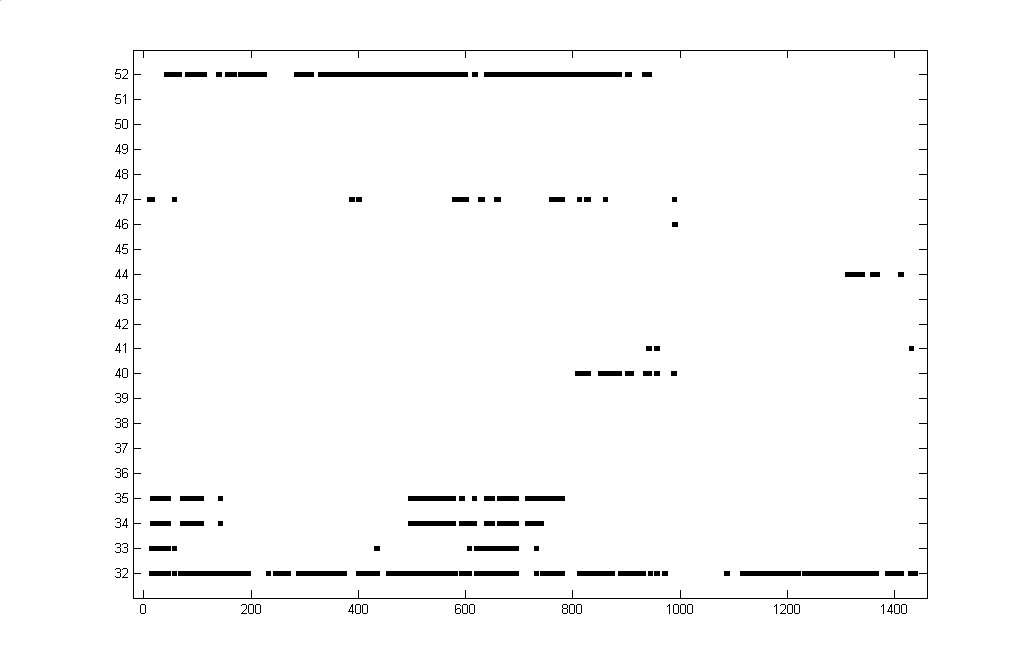 |
| h72AON2 (+)  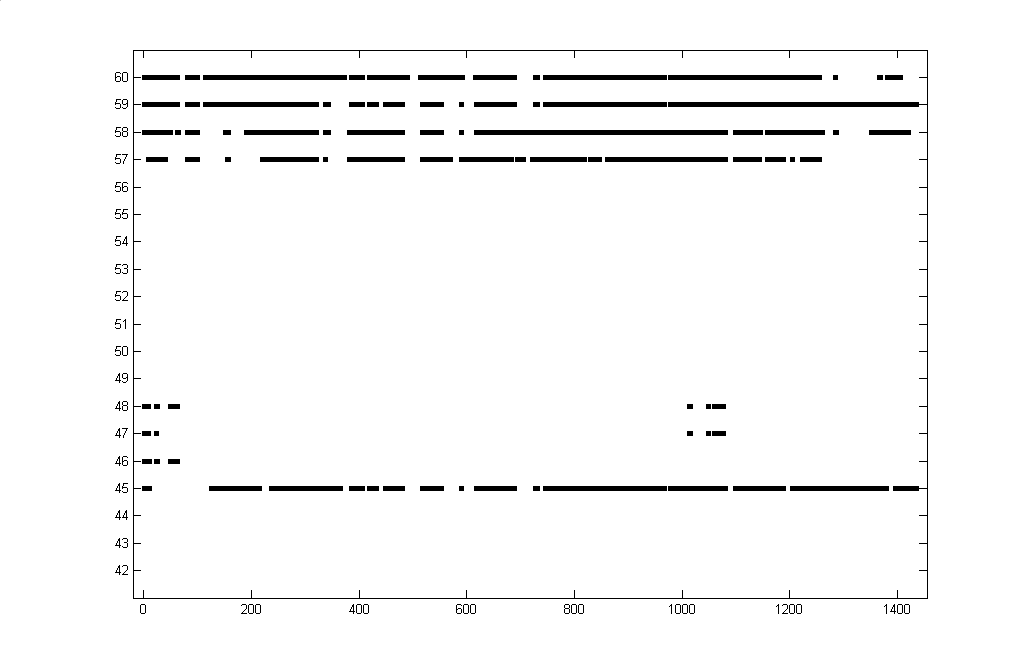 | h73AON2 (+)  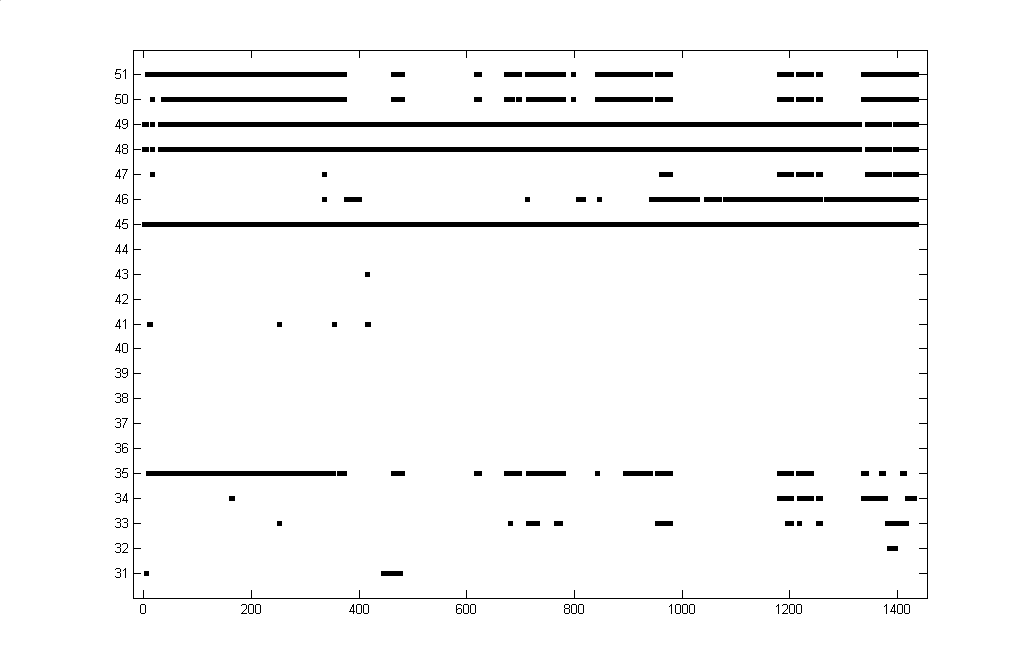 | h74AON2 (+)  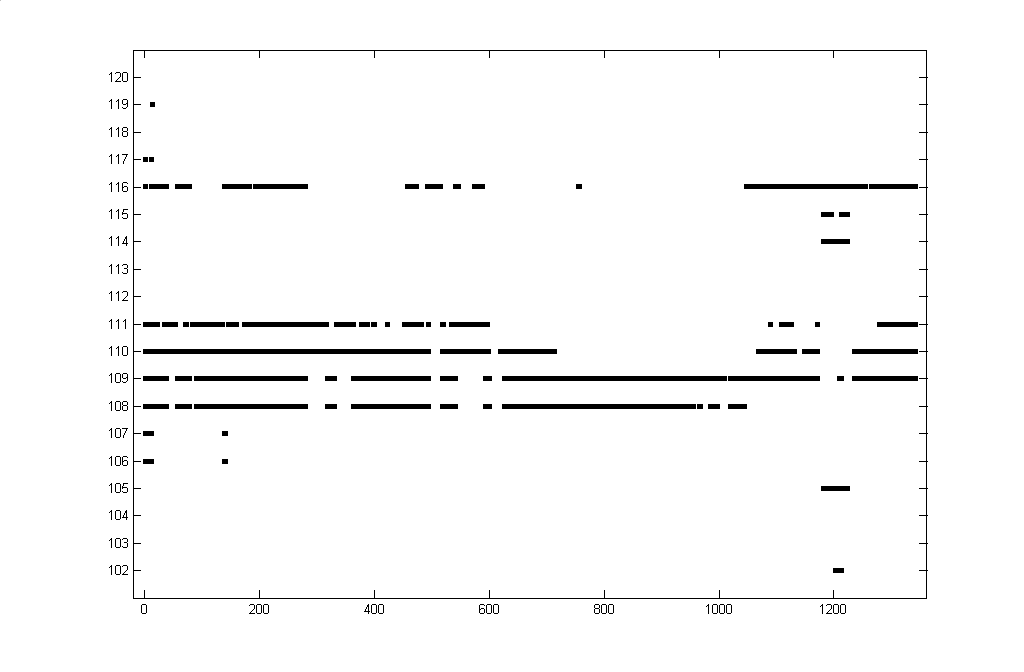 |
| h76AON2 (+)  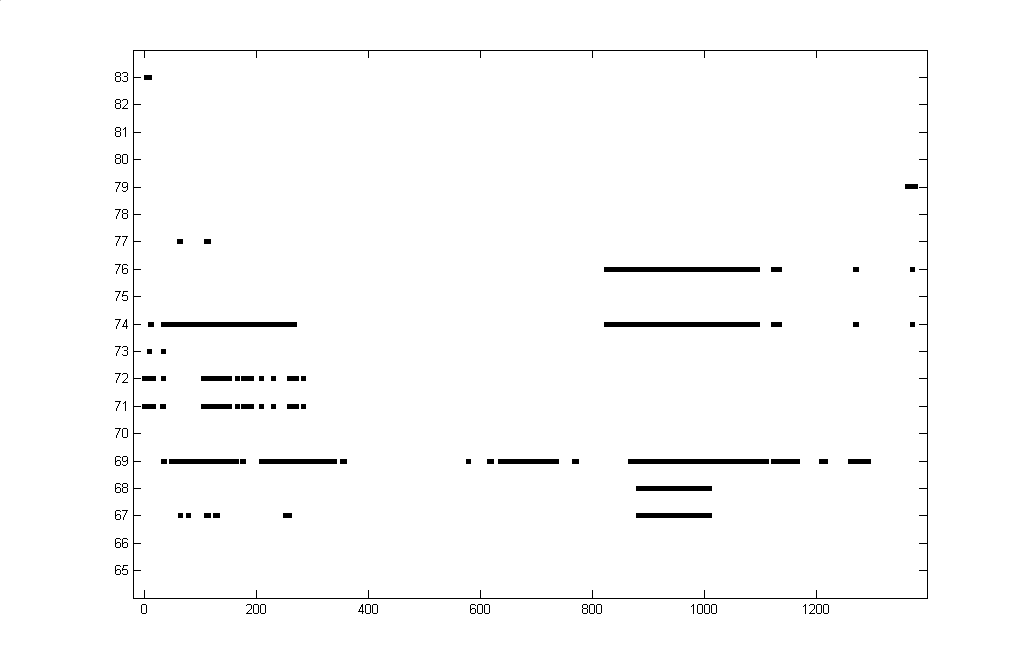 | h2AON2 (─)  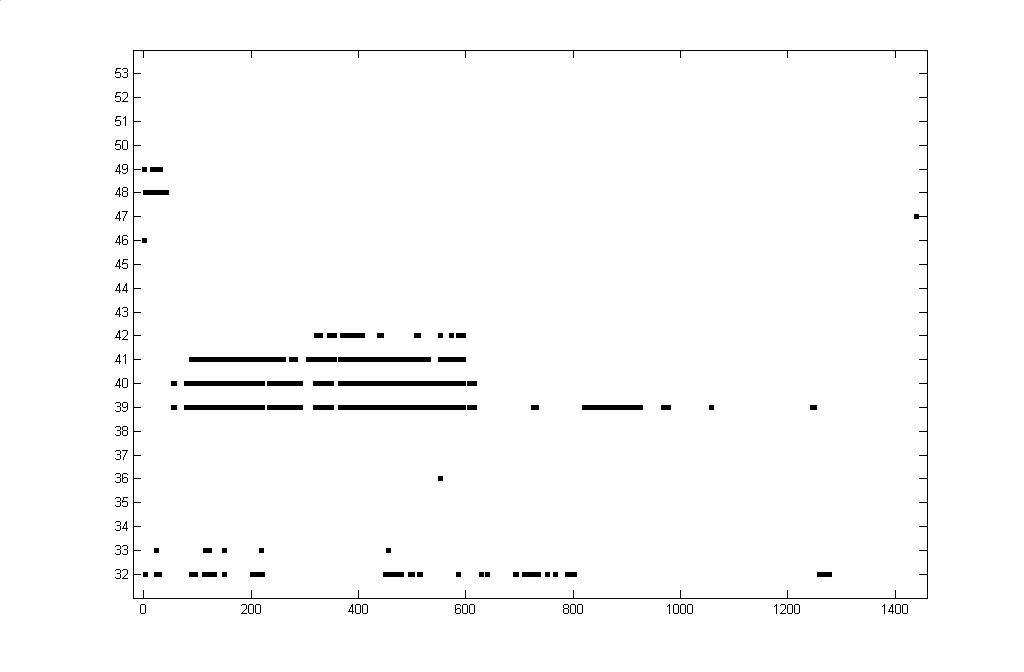 | h2AON3 (─)  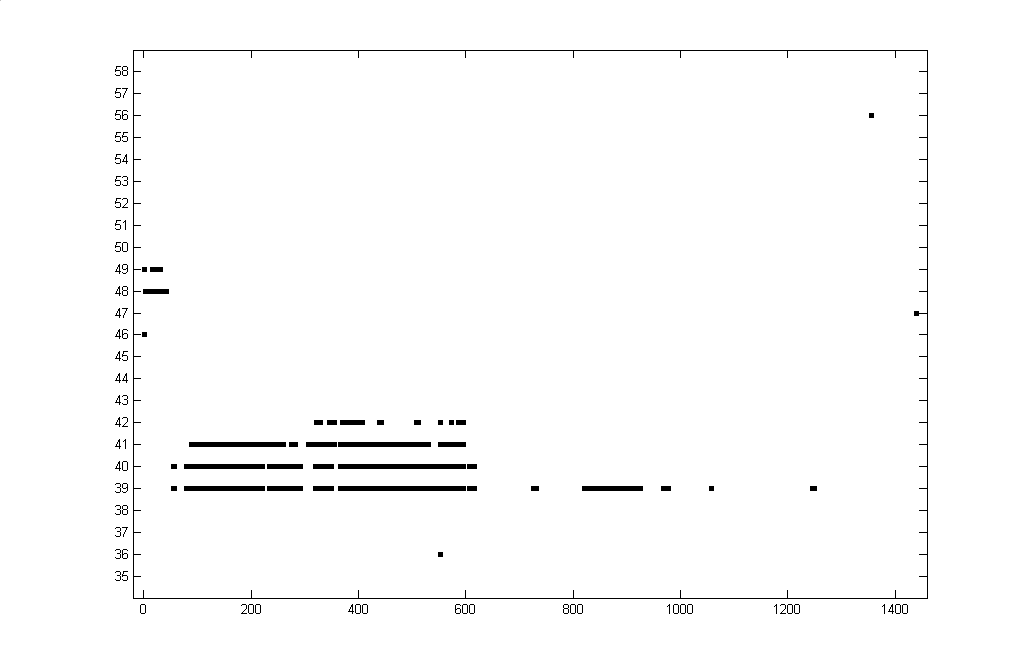 |
| h29AON10 (─)  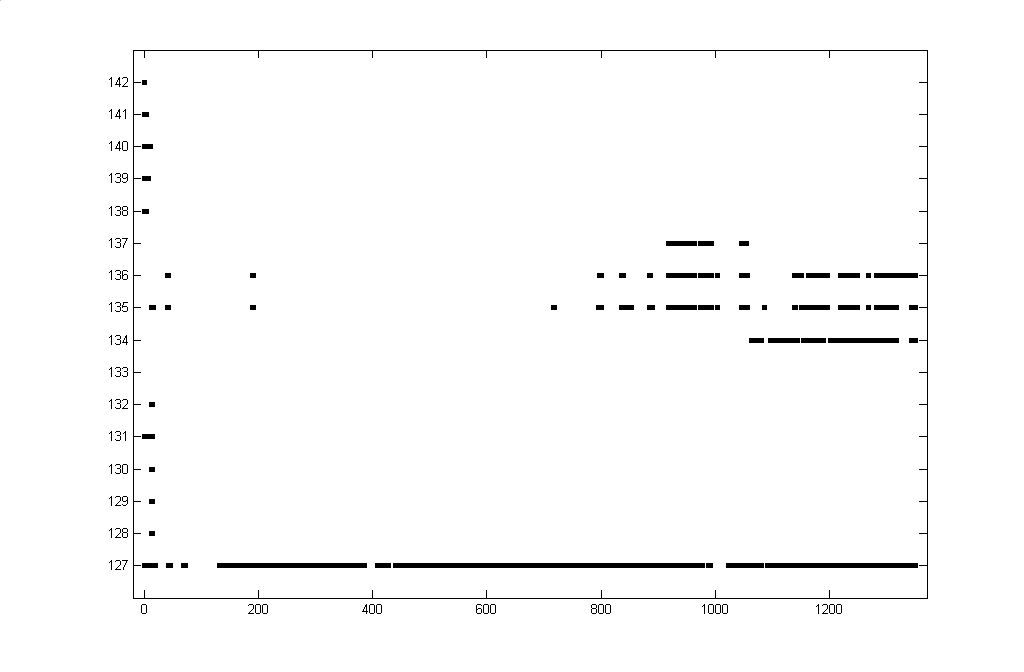 | h43AON1 (─)  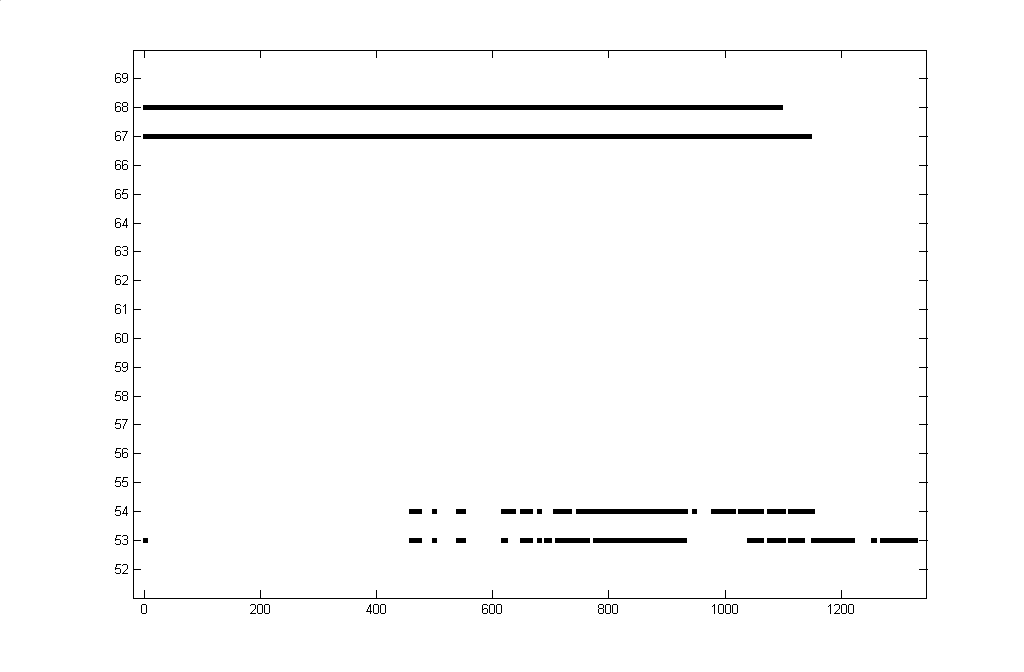 | h43AON3 (─)  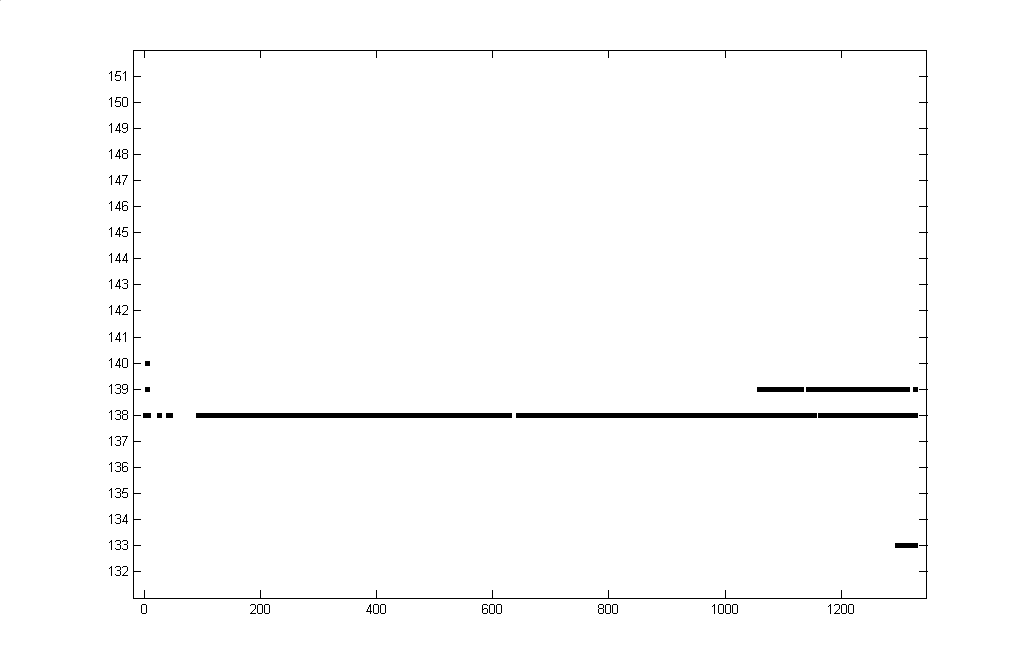 |
| h43AON4 (─)  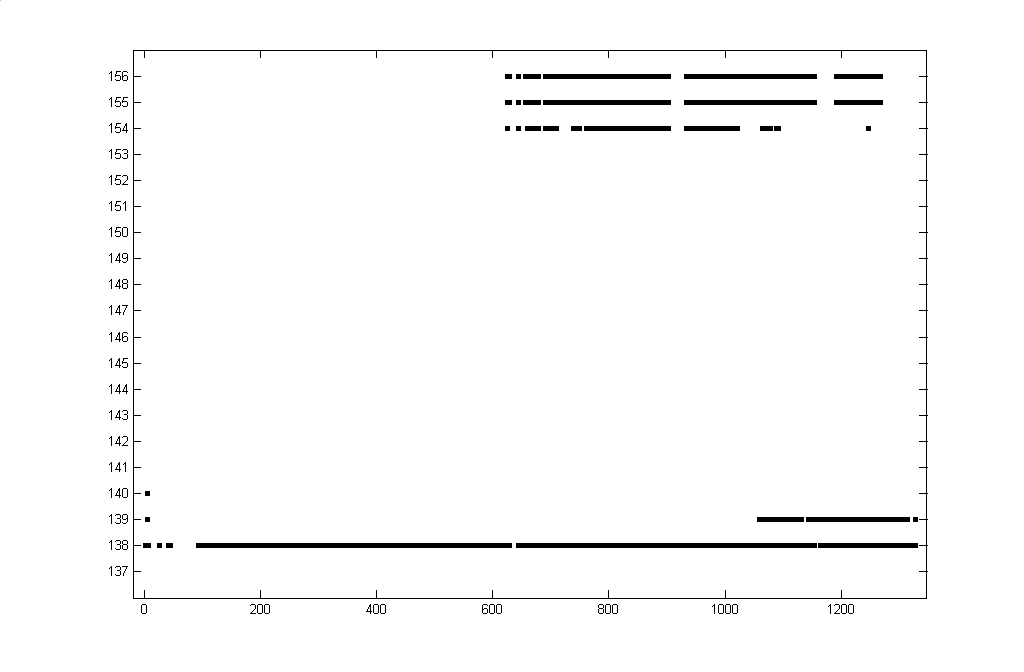 | h45AON1 (─)  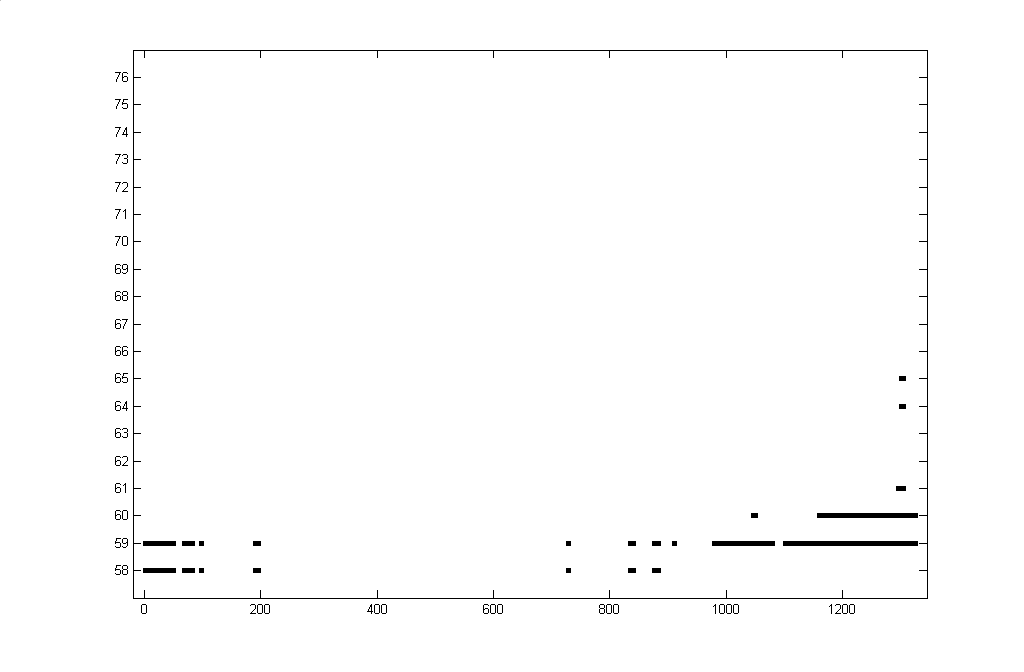 | h45AON2 (─)  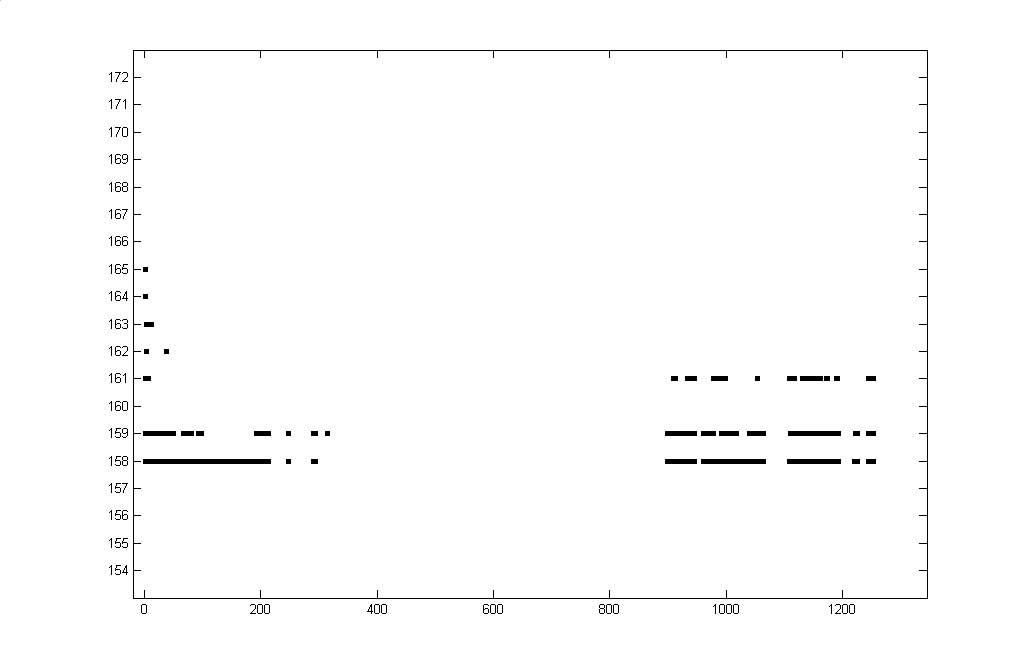 |
| h45AON3 (─)  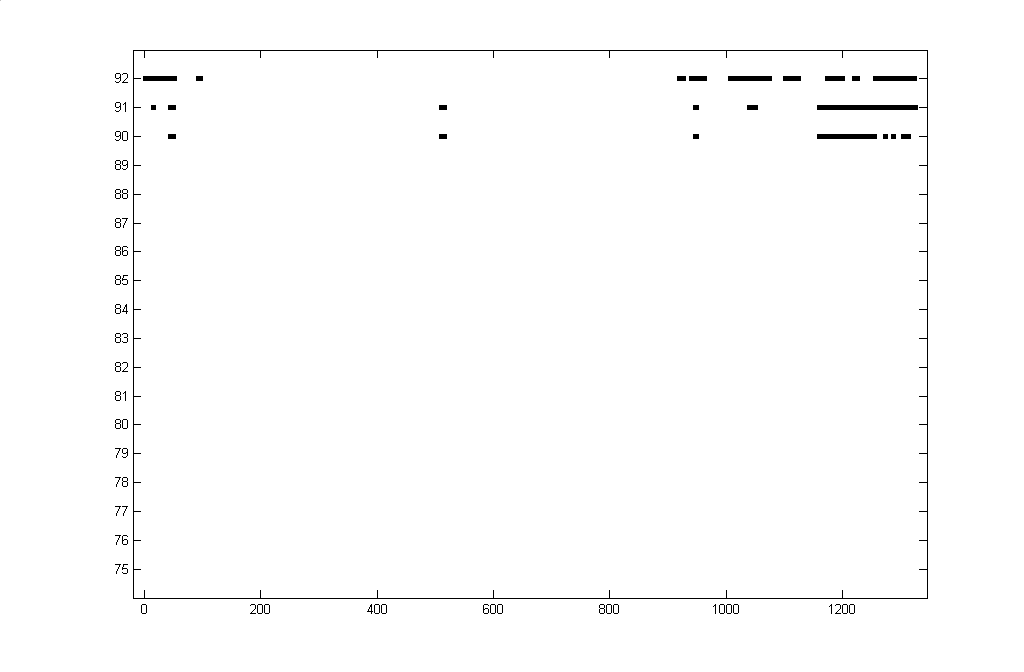 | h45AON4 (─)  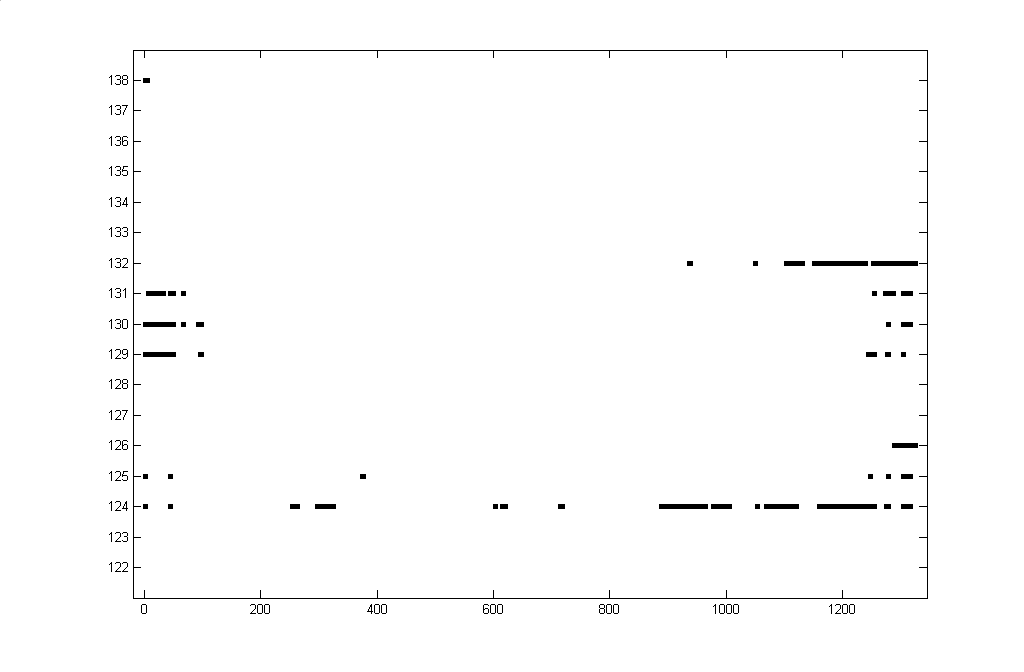 | h45AON9 (─)  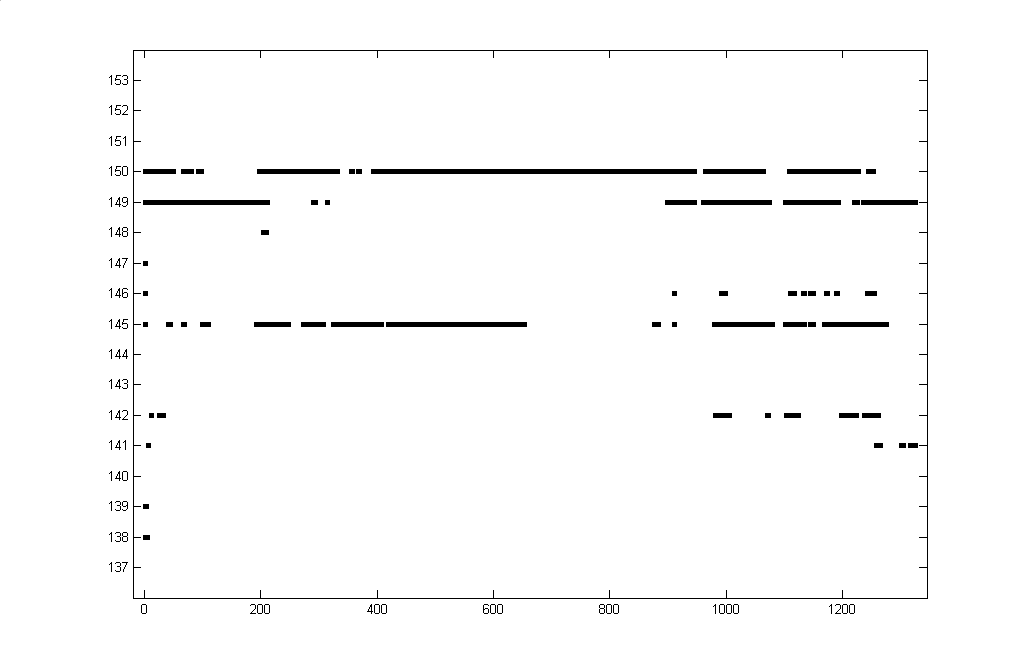 |
| h46AON9 (─)  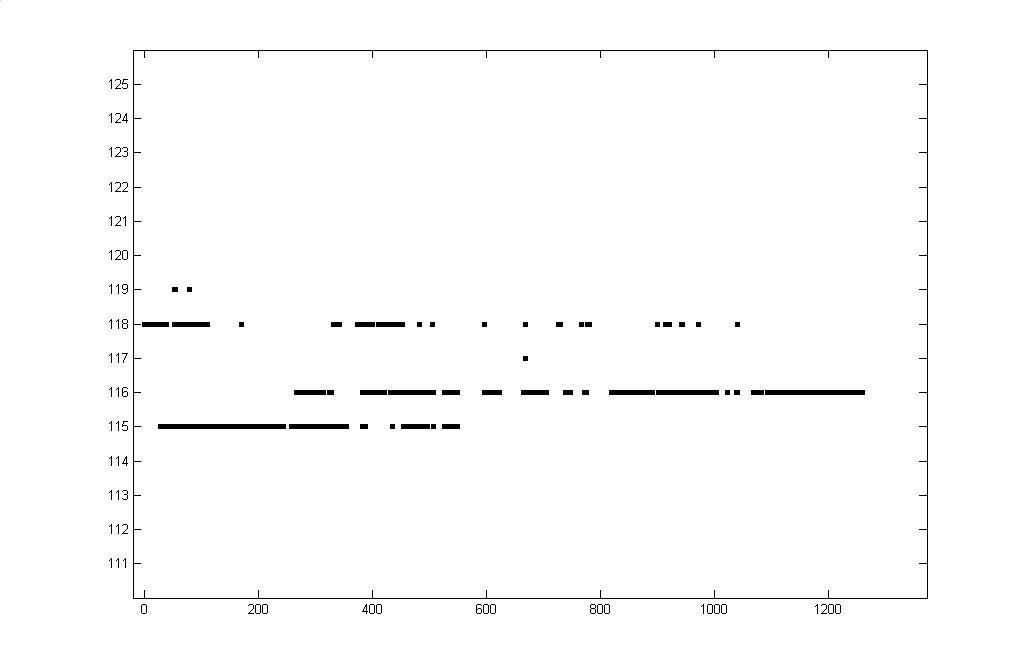 | h46AON21 (─)  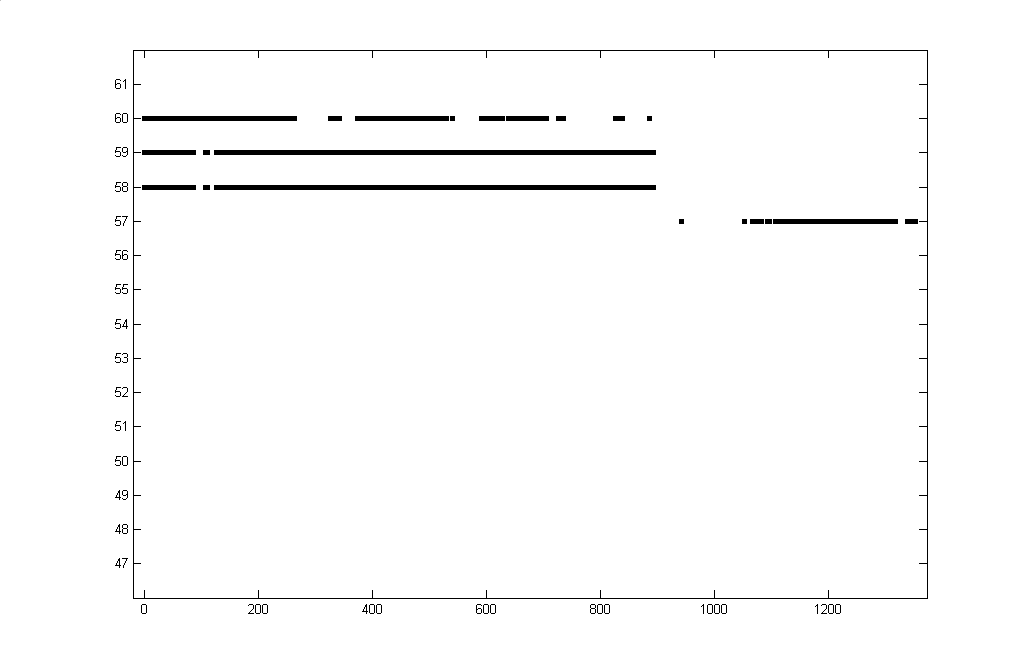 | h47AON1 (─)  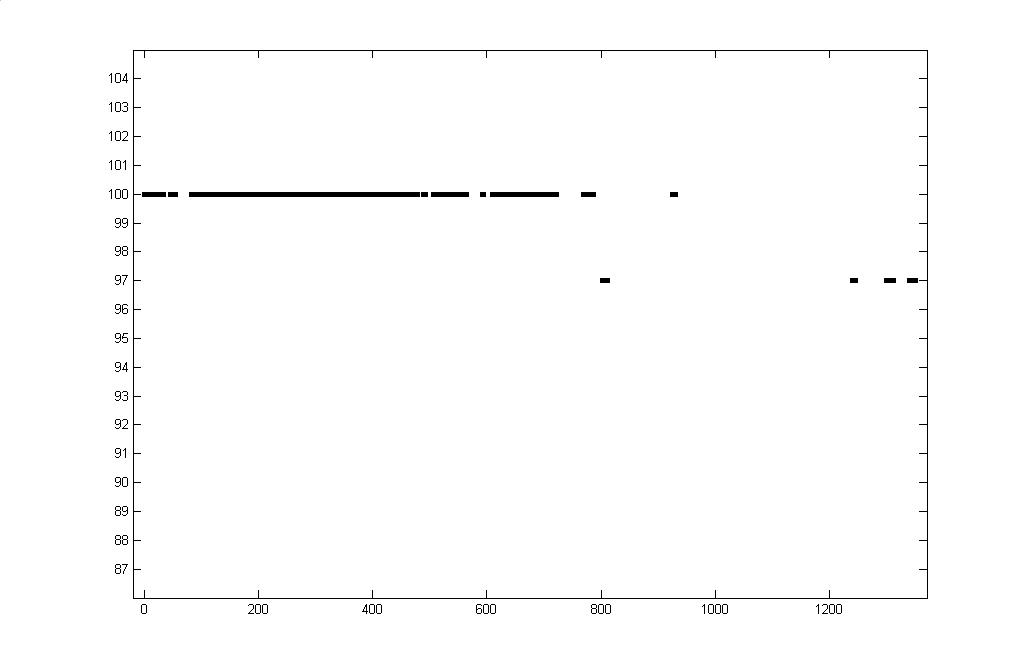 |
| h47AON2 (─)  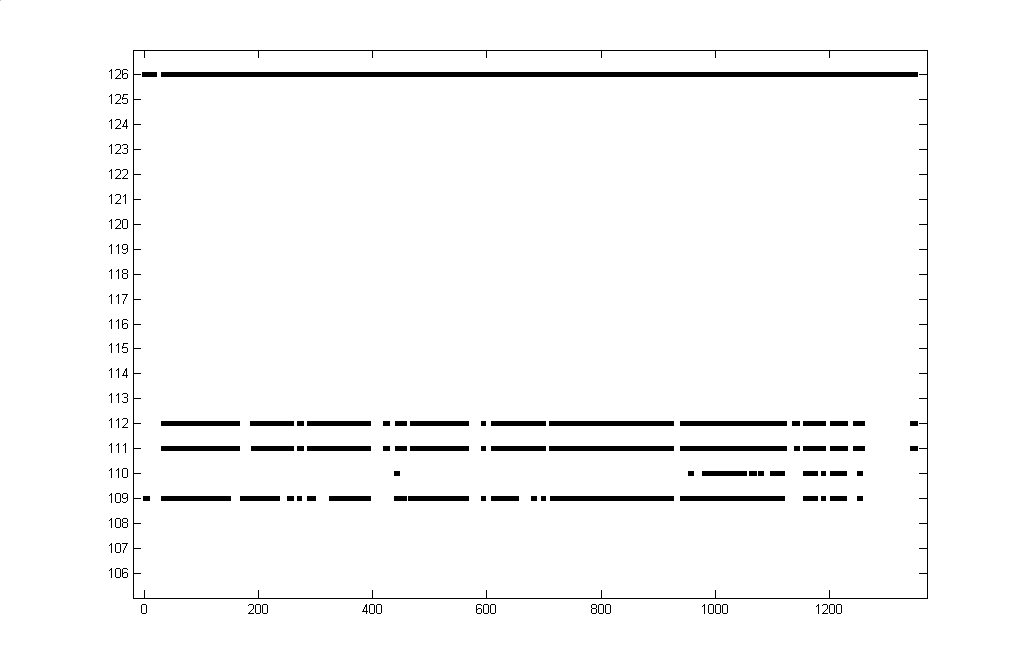 | h47AON3 (─)  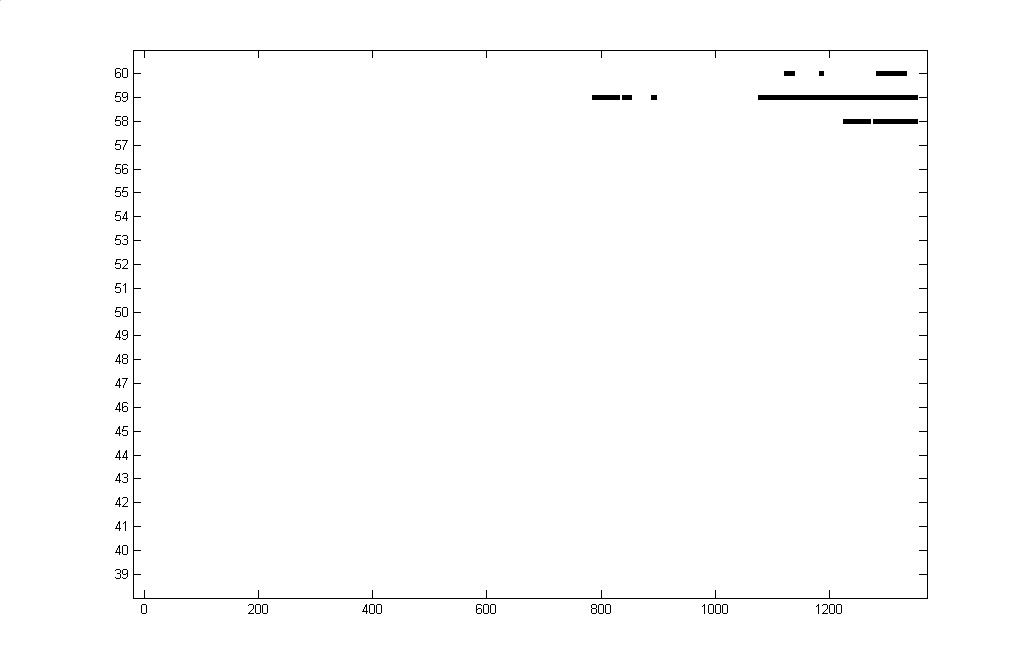 | h47AON4 (─)  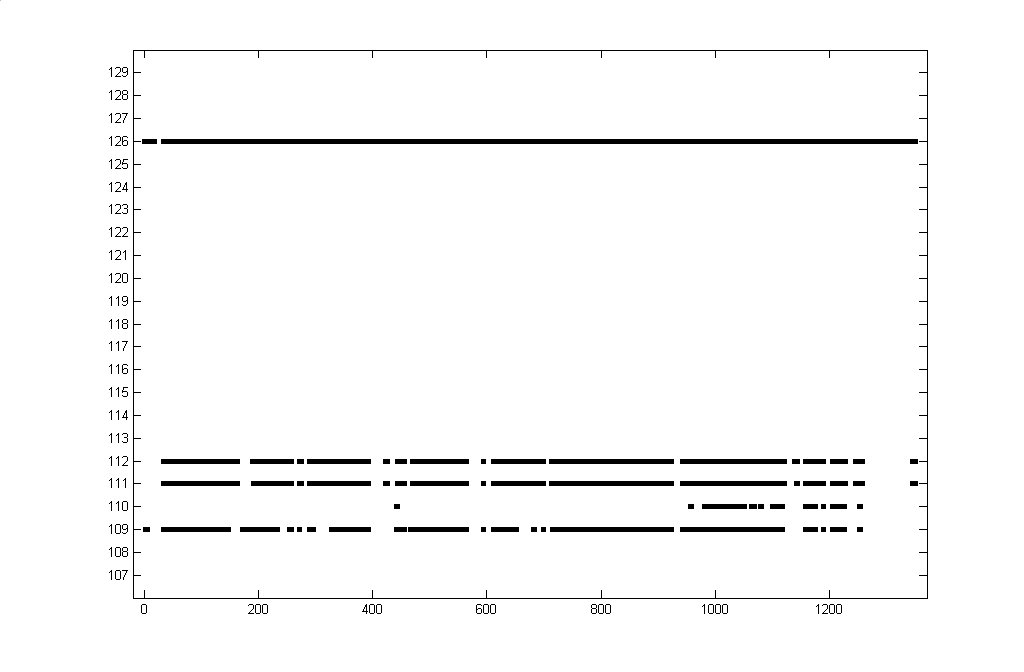 |
| h47AON5 (─)  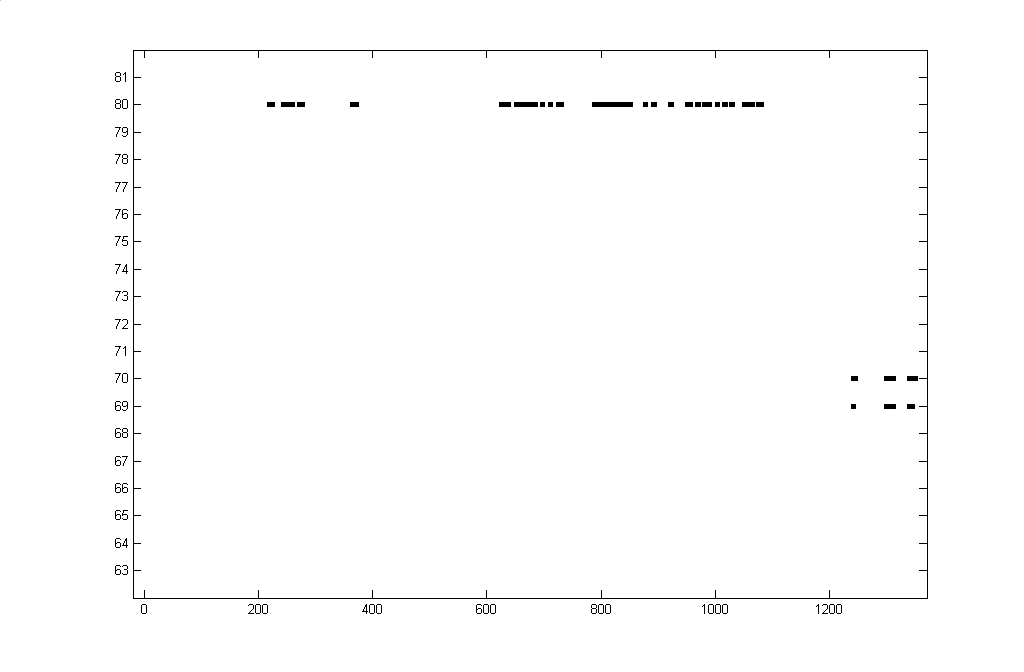 | h47AON6 (─)  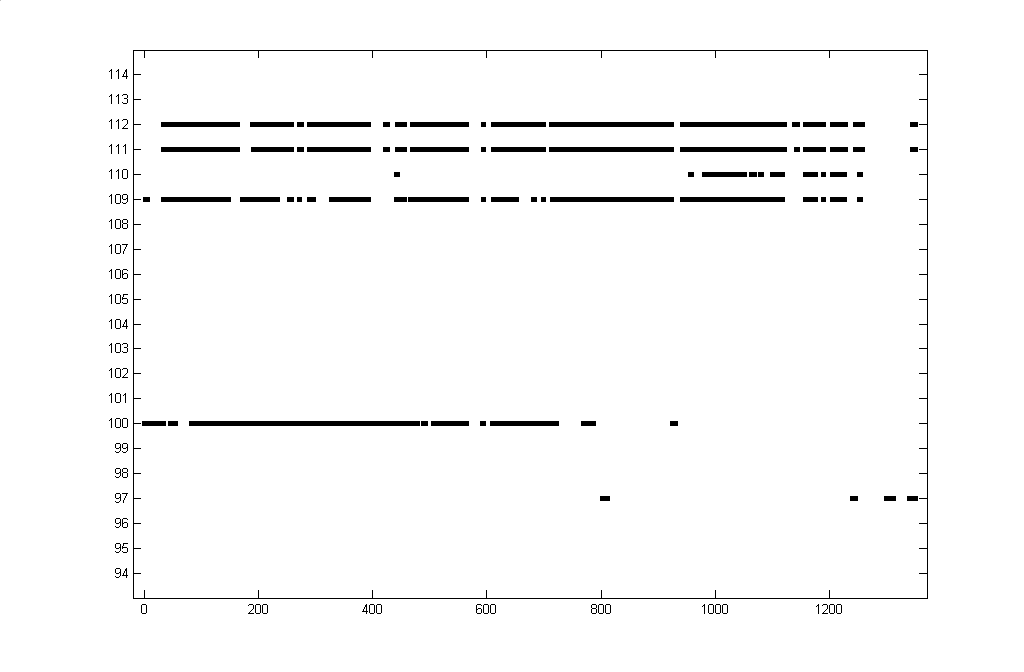 | h48AON1 (─)  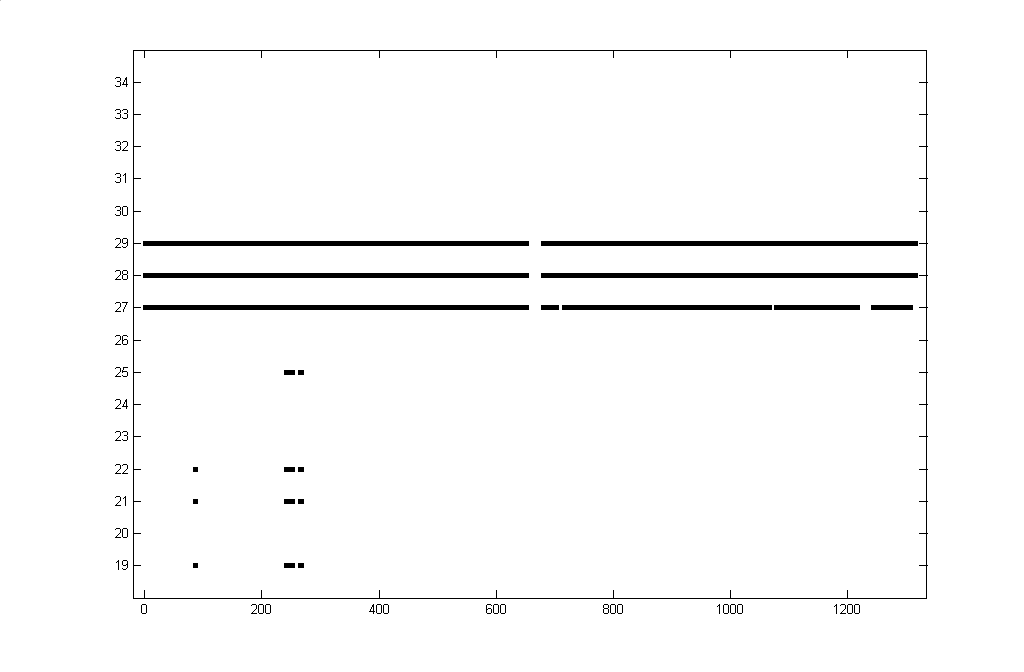 |
| h48AON2 (─)  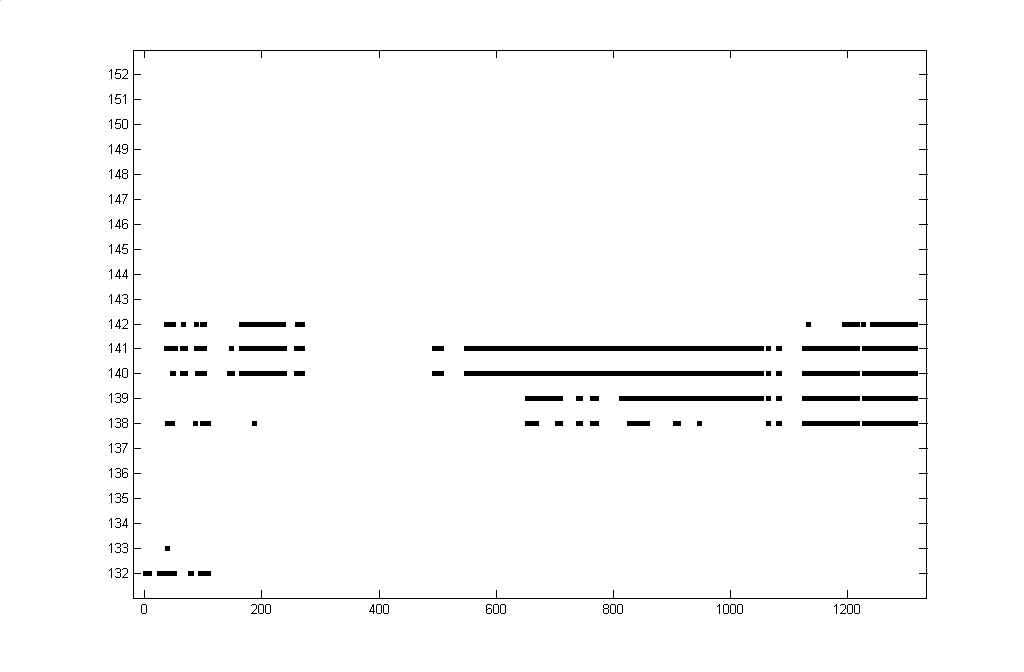 | h48AON3 (─)  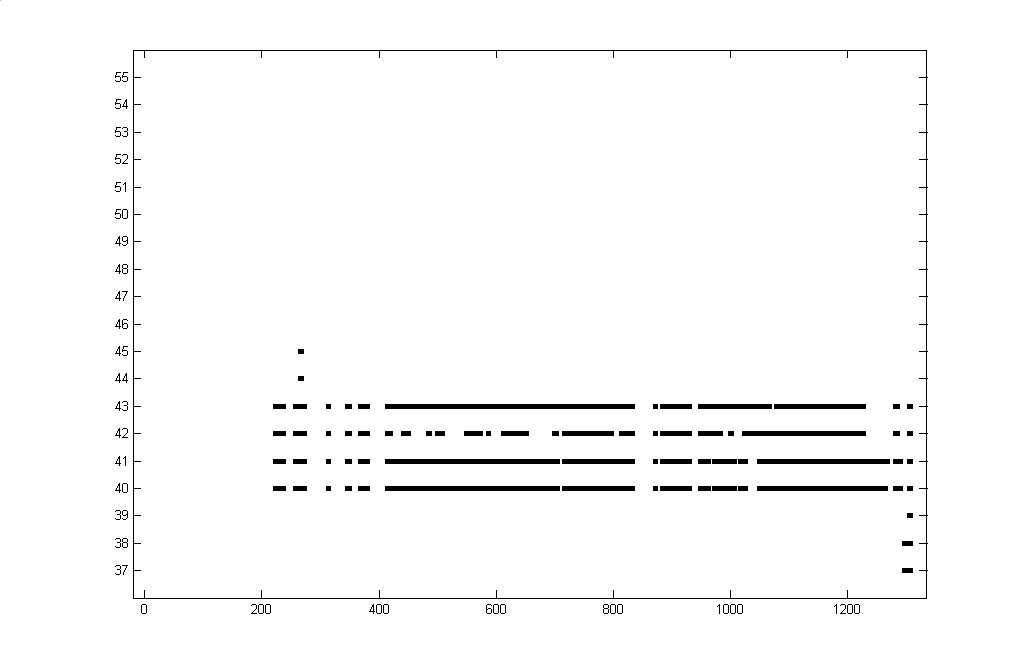 | h48AON4 (─)  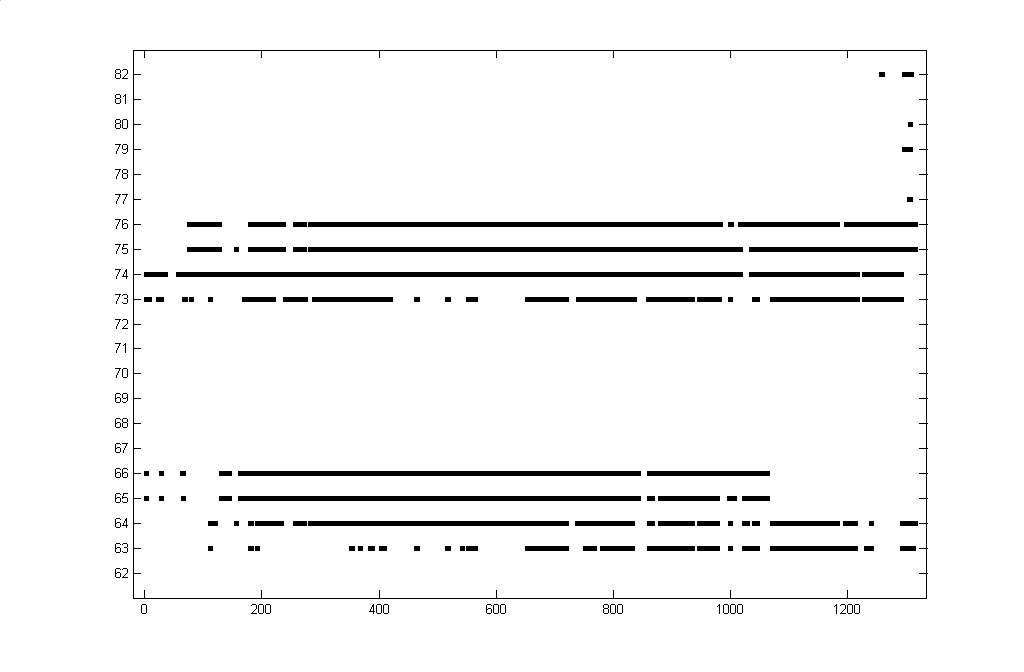 |
| h48AON8 (─)  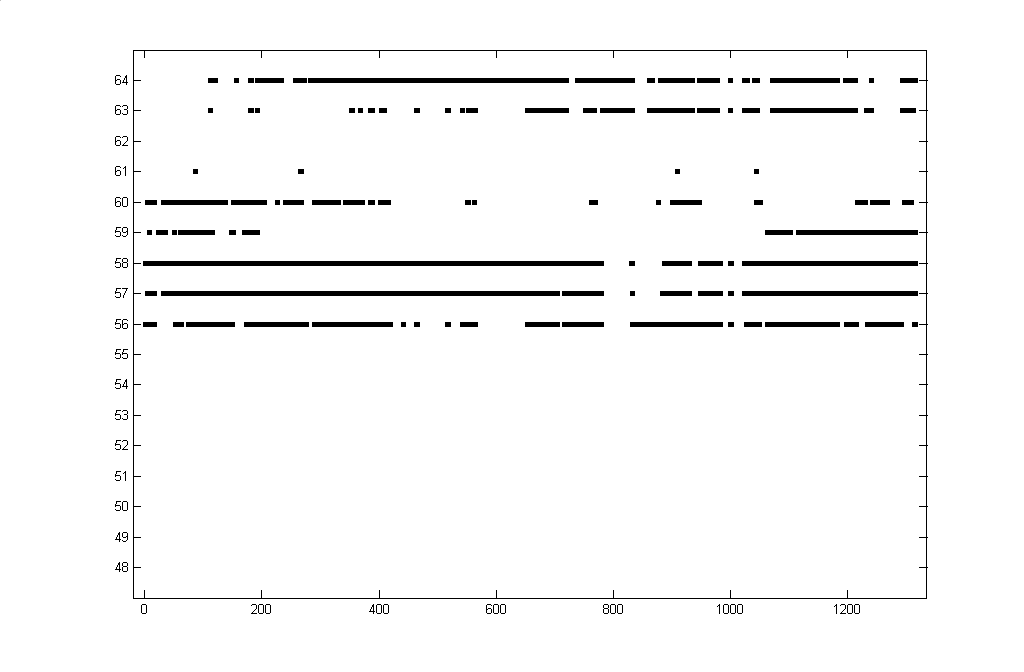 | h51AON24 (─) | h51AON27 (─) |
| h52AON2 (─) | h53AON2 (─) | h56AON2 (─) |
| h57AON1 (─) | h57AON2 (─) | h57AON3 (─) |
| h58AON1 (─) | h59AON1 (─) | h60AON2 (─) |
| h61AON1 (─) | h62AON2 (─) | h76AON1 (─) |
|  | | |
| H2A (++) | H3A (++) | H4A (++) |
| H5A (++) | H6A (++) | H7A (++) |
| H12A (++) | H13A (++) | H14A (++) |
| H15A (++) | H18A (++) | H19A (++) |
| H22A (++) | H23A (++) | H24A (++) |
| H25A (++) | H27A (++) | H28A (++) |
| H29A (++) | H30A (++) | H32A (++) |
| H33A (++) | H35A (++) | H37A (++) |
| H38A (++) | H39A (++) | H41A (++) |
| H51A (++) | H52A (++) | H53A (++) |
| H72A (++) | H74A (++) | H75A (++) |
| H77A (++) | H78A (++) | H11A (+ ^1^) |
| H21A (+ ^1^) | H36A (+ ^1^) | H43A (+ ^1^) |
| H44A (+ ^1^) | H46A (+ ^1^) | H60A (+ ^1^) |
| H61A (+ ^1^) | H68A (+ ^1^) | H70A (+ ^1^) |
| H73A (+ ^1^) | H50A (+ ^2^) | H55A (+ ^2^) |
| H56A (+ ^2^) | H58A (+ ^2^) | H62A (+ ^2^) |
| H63A (+ ^2^) | H64A (+ ^2^) | H67A (+ ^2^) |
| H76A (+ ^2^) | H10A2 (–) | H20A1 (–) |
| H20A2 (–) | H34A1 (–) | H34A2 (–) |
| H54A (–) | H65A2 (–) |  |
