# Supplementary material for: Dynamics of Co-Transcriptional Pre-mRNA Folding Influences the Induction of Dystrophin Exon Skipping by Antisense Oligonucleotides
Source: PLoS One. 2008 Mar 26;3(3):e1844. doi: 10.1371/journal.pone.0001844 (PMC2267000; doi:10.1371/journal.pone.0001844)
Supplement: Table S4 — p-values for K-S tests using the third level score (L3) and fourth level scores (L4_AVG, L4_AND and L4_OR) as test variables between Set A and Set B. (A) (++) and (+) AONs are tested between Set A and Set B for statistical difference using L3 as test variable. (B) to (E) Fourth level scores as test variables for engaged nucleotides localized at (B) both 3′ and 5′ ends, (C) at 3′ end, (D) at 5′ end and (E) away from the ends of the AON target sites Note: (-) AONs between the two sets cannot be tested because the sample size in Set B is too small to confer statistical confidence. (0.07 MB DOC) [file pone.0001844.s007.doc]

**Table S4. p-values for K-S tests using the third level score (*L3*) and fourth level scores (*L4_AVG*, *L4_AND* and *L4_OR*) as test variables between Set A and Set B. (A)** (++) and (+) AONs are tested between Set A and Set B for statistical difference using *L3* as test variable. **(B)** to **(E)** Fourth level scores as test variables for *engaged* nucleotides localized at **(B)** both 3’ and 5’ ends, **(C)** at 3’ end, **(D)** at 5’ end and **(E)** away from the ends of the AON target sites Note: (–) AONs between the two sets cannot be tested because the sample size in Set B is too small to confer statistical confidence.

|  | **H_o_:**  **Set A vs. Set B** | ***L3*** | |
| --- | --- | --- | --- |
|  |  | **1^st^ < 2^nd^** | **1^st^ > 2^nd^** |
| **A** | (++) | 0.051 | 0.91 |
|  | (+) | 0.37 | 0.99 |

|  | **H_o_:**  **Set A vs. Set B** | ***L4_AVG*** | | ***L4_AND*** | | ***L4_OR*** | |
| --- | --- | --- | --- | --- | --- | --- | --- |
|  |  | **1^st^ < 2^nd^** | **1^st^ > 2^nd^** | **1^st^ < 2^nd^** | **1^st^ > 2^nd^** | **1^st^ < 2^nd^** | **1^st^ > 2^nd^** |
| **B** | 3’ and 5’ ends (++) | 0.23 | 0.88 | 0.76 | 0.76 | 0.14 | 0.89 |
|  | 3’ and 5’ ends (+) | 0.24 | 0.92 | 0.40 | 0.94 | 0.24 | 0.98 |
| **C** | 3’ end (++) | 0.11 | 0.91 | 0.49 | 0.25 | 0.11 | 0.92 |
|  | 3’ end (+) | 0.36 | 0.95 | 0.50 | 0.72 | 0.22 | 0.98 |
| **D** | 5’ end (++) | 0.60 | 0.59 | 0.75 | 0.61 | 0.73 | 0.58 |
|  | 5’ end (+) | 0.23 | 0.71 | 0.23 | 0.98 | 0.41 | 0.87 |
| **E** | 2-nt group (++) | 0.97 | 0.16 | 0.97 | 0.25 | 0.80 | 0.30 |
|  | 2-nt group (+) | 0.75 | 0.90 | 0.48 | 0.53 | 0.80 | 0.75 |

*Set A vs. Set B*. The results in this work support the conclusion that the efficacy and efficiency of AONs that target shorter AON target sites (Set A) are more vulnerable to the presence of *engaged* nucleotides than AONs that target longer AON target sites (Set B). To ascertain that this dissimilarity in vulnerability is not caused by differences in the manifestation of *engaged* nucleotides between the two sets, the third and fourth level scores of AONs of the same grade are each compared between the two sets by K-S tests. TABLE S5A shows that there is no significant difference in the frequency of *engaged* nucleotides between Sets A and B for (++) and (+) AONs. Similarly, both Sets A and B have statistically similar localizations of consecutive *engaged* nucleotides in the sequence of steps of transcriptional analysis in their AON target sites (TABLES S4B – S4E). Therefore, AONs of the same grade have similar manifestation of *engaged* nucleotides between the two sets.
