# Supplementary material for: Dynamics of Co-Transcriptional Pre-mRNA Folding Influences the Induction of Dystrophin Exon Skipping by Antisense Oligonucleotides
Source: PLoS One. 2008 Mar 26;3(3):e1844. doi: 10.1371/journal.pone.0001844 (PMC2267000; doi:10.1371/journal.pone.0001844)
Supplement: Table S5 — Number of predicted secondary structures generated in each exon. For every exon in dystrophin gene, the total number of secondary structures predicted as well as the average number of predicted secondary structures per step of transcriptional analysis is tabulated. (0.12 MB DOC) [file pone.0001844.s008.doc]

**Table S5.** **Number of predicted secondary structures generated in each exon.** For every exon in dystrophin gene, the total number of secondary structures predicted as well as the average number of predicted secondary structures per step of transcriptional analysis is tabulated.

| **Exon** | **Total number of predicted secondary structures** | **Average number of predicted secondary structures per step of transcriptional analysis** | **Exon** | **Total number of predicted secondary structures** | **Average number of predicted secondary structures per step of transcriptional analysis** |
| --- | --- | --- | --- | --- | --- |
| **1** | 53,610 | 47 | **41** | 42,191 | 32 |
| **2** | 46,976 | 33 | **42** | 41,393 | 32 |
| **3** | 49,704 | 35 | **43** | 48,974 | 37 |
| **4** | 45,683 | 32 | **44** | 52,391 | 39 |
| **5** | 50,760 | 36 | **45** | 42,025 | 32 |
| **6** | 44,287 | 33 | **46** | 41,167 | 30 |
| **7** | 48,503 | 35 | **47** | 52,093 | 39 |
| **8** | 39,900 | 30 | **48** | 36,877 | 28 |
| **9** | 46,709 | 34 | **49** | 45,461 | 32 |
| **10** | 39,708 | 30 | **50** | 42,703 | 31 |
| **11** | 40,661 | 31 | **51** | 39,401 | 31 |
| **12** | 37,624 | 28 | **52** | 43,384 | 31 |
| **13** | 47,411 | 34 | **53** | 37,233 | 29 |
| **14** | 46,746 | 33 | **54** | 44,714 | 33 |
| **15** | 46,900 | 34 | **55** | 39,718 | 30 |
| **16** | 49,433 | 37 | **56** | 47,066 | 35 |
| **17** | 41,410 | 31 | **57** | 41,701 | 31 |
| **18** | 53,801 | 39 | **58** | 43,278 | 31 |
| **19** | 51,808 | 37 | **59** | 35,323 | 29 |
| **20** | 32,577 | 26 | **60** | 41,692 | 31 |
| **21** | 43,575 | 33 | **61** | 45,002 | 32 |
| **22** | 41,130 | 30 | **62** | 48,955 | 34 |
| **23** | 46,009 | 36 | **63** | 58,279 | 40 |
| **24** | 50,268 | 36 | **64** | 49,903 | 35 |
| **25** | 39,331 | 29 | **65** | 46,491 | 36 |
| **26** | 41,578 | 31 | **66** | 49,970 | 35 |
| **27** | 42,147 | 32 | **67** | 42,516 | 32 |
| **28** | 45,928 | 34 | **68** | 42,470 | 32 |
| **29** | 41,464 | 31 | **69** | 45,088 | 32 |
| **30** | 46,507 | 35 | **70** | 40,765 | 30 |
| **31** | 33,995 | 24 | **71** | 52,467 | 36 |
| **32** | 42,935 | 32 | **72** | 46,199 | 32 |
| **33** | 39,985 | 30 | **73** | 53,428 | 37 |
| **34** | 49,348 | 37 | **74** | 49,686 | 37 |
| **35** | 40,462 | 31 | **75** | 36,130 | 29 |
| **36** | 39,705 | 29 | **76** | 37,509 | 27 |
| **37** | 44,209 | 33 | **77** | 36,726 | 26 |
| **38** | 44,387 | 32 | **78** | 47,513 | 32 |
| **39** | 46,117 | 34 | **79** | 49,713 | 33 |
| **40** | 51,086 | 38 |  |  |  |
